# Proteome of larval metamorphosis induced by epinephrine in the Fujian oyster *Crassostrea angulata*

**DOI:** 10.1186/s12864-020-07066-z

**Published:** 2020-09-29

**Authors:** Guilan Di, Xiaohuo Xiao, Ming Him Tong, Xinhua Chen, Li Li, Miaoqin Huang, Long Zhou, Caihuan Ke

**Affiliations:** 1 State Key Laboratory of Marine Environmental Science, College of Ocean and Earth Sciences, Xiamen University, Xiamen, 361005, China; 2 College of Fisheries, Henan Normal University, Xinxiang, 453007 China; 3 Key Laboratory of Marine Biotechnology of Fujian Province, Institute of Oceanology, Fujian Agriculture and Forestry University, Fuzhou, 350002, Fujian Province China; 4 Department of Chemistry, Marine Biodiscovery Centre, the University of Aberdeen, Aberdeen, Scotland AB24 3UE UK

**Keywords:** *Crassostrea angulata*, Settlement, Metamorphosis, Proteomic, Epinephrine

## Abstract

**Background:**

The Fujian oyster *Crassostrea angulata* is an economically important species that has typical settlement and metamorphosis stages. The development of the oyster involves complex morphological and physiological changes, the molecular mechanisms of which are as yet unclear.

**Results:**

In this study, changes in proteins were investigated during larval settlement and metamorphosis of *Crassostrea angulata* using epinephrine induction. Protein abundance and identity were characterized using label-free quantitative proteomics, tandem mass spectrometry (MS/ MS), and Mascot methods. The results showed that more than 50% (764 out of 1471) of the quantified proteins were characterized as differentially expressed. Notably, more than two-thirds of the differentially expressed proteins were down-regulated in epinephrine-induced larvae. The results showed that “metabolic process” was closely related to the development of settlement and metamorphosis; 5 × 10^− 4^ M epinephrine induced direct metamorphosis of larvae and was non-toxic. Calmodulin and MAPK pathways were involved in the regulation of settlement of the oyster. Expression levels of immune-related proteins increased during metamorphosis. Hepatic lectin-like proteins, cadherins, calmodulin, calreticulin, and cytoskeletal proteins were involved in metamorphosis. The nervous system may be remodeled in larval metamorphosis induced by epinephrine. Expression levels of proteins that were enriched in the epinephrine signaling pathway may reflect the developmental stage of the larvae, that may reflect whether or not larvae were directly involved in metamorphosis when the larvae were treated with epinephrine.

**Conclusion:**

The study provides insight into proteins that function in energy metabolism, immune responses, settlement and metamorphosis, and shell formation in *C. angulata*. The results contribute valuable information for further research on larval settlement and metamorphosis.

**Graphical abstract:**

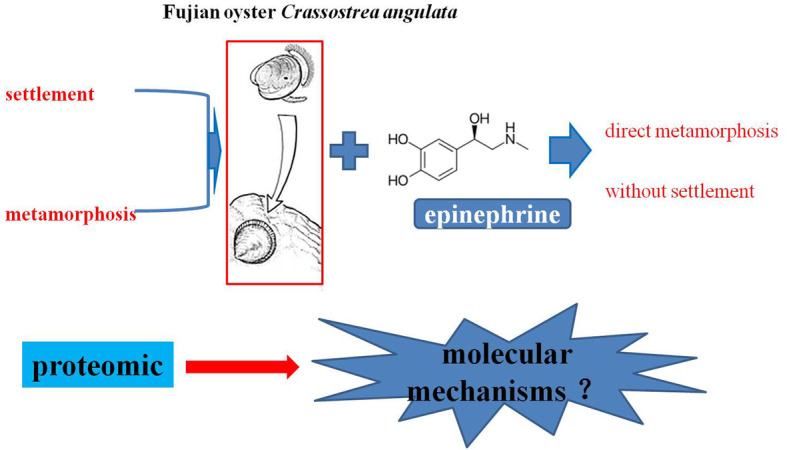

## Highlights

Seven hundred sixt-four of 1471 quantified proteins are differentially expressed in *Crassostrea angulata.*

10^− 5^ M epinephrine can induce direct metamorphosis of larvae.

Proteins of adrenaline signaling pathway may reflect the development stage of larvae.

Transcriptional regulation is involved in the process of settlement and metamorphosis.

Proteins were involved in immune responses, metamorphosis and shell formation.

## Background

Oysters are a group of commercially important species cultured along the coast of China. The annual production of oysters was about 4.88 million tonnes in 2017 (China Fishery Statistical Yearbook for 2018) [1]. The Fujian oyster *Crassostrea angulata* accounts for about 50% of the total oyster production in China [2, 3].

Embryonic and larval development are key phylogenetic events [4], and embryological research contributes to the development of environmental pollution monitoring and the aquaculture industry [5]. Size and age at reproduction of the offspring may have important consequences for population dynamics and demography [6]; larval developmental plasticity is a crucial source of variation and can directly influence the evolution of populations and species in adult phenotypes [7, 8]. Oysters are a typical two-phase life cycle species that have a pelagic phase and a benthonic phase; the transition between the two forms usually occurs rapidly [9]. Larval settlement and metamorphosis are importmant transition periods associated with the evolution of mollusks, and these factors also impact population distribution, phenotypic differentiation, and speciation [10, 11]. Even in cultured oysters, metamorphosis is a crucial step for the aquaculture facility, because oysters that have successfully settled usually show increased survival.

Metamorphosis of oysters includes complex morphological and behavioral changes that are irreversible and that are essential for survival. Pre-settlement or post-metamorphosis larvae are proposed to be regulated by endogenous chemical signals and endocrine proteins. However, the molecular mechanisms underlying metamorphosis of model species, such as frogs and fruit flies, have been studied in much greater depth than those in marine mollusks. In the Fujian oyster, Cacaspase-2, Cacaspase-3 mRNA, and settlement and metamorphosis-related protein (SMRP1) are highly expressed in larvae during settlement and metamorphosis [12, 13].

Cranfield et al. described the behaviour of *Ostrea edulis* before attachment [14–16]. Ke and Feng [17] described the settlement process of *Crassostrea angulata*. The choice of settlement substratum is critical because an inappropriate settlement substratum is often fatal to juveniles [18]. Scallop or oyster shells are often used as substrata in oyster farms; the shells are placed into cages for raft- or long-line culture [19]. In addition, the traditional farming methods are laborous and costly; food acquisition and growth rate of the oysters are often affected by the high-density farming that causes large individual differences and reduced economic value. Thus, it is important to understand the breeding biology of oysters.

Oyster hatcheries have been developing multiple methods for clutchless oyster spat production for many years [20]. The most widely used inducer for oysters is epinephrine (EPI, also known as adrenaline) [21]. Epinephrine induction has shown the capability to generate non-attached spat (juveniles) in various oyster species [20, 22, 23]. Coon et al. [20] report that EPI and norepinephrine are able to induce cultchless spats, demonstrating that EPI is able to induce oyster metamorphosis without settlement or negative effects on survival and development [20]. In comparison with oysters attached to the substratum, clutchless oysters are superior in shape and are more uniform, traits that are economically favorable [24]. Thus, the use of EPI has become a common practice in many bivalve hatcheries [25, 26]. EPI has been widely used to induce the settlement and metamorphosis of bivalve mollusks [25, 27–30]. The positive effects of EPI on metamorphosis in a wide range of bivalve species have been summarised recently [31].

Nonetheless, the molecular mechanisms of EPI induction are unclear. Coon et al. proposed that adrenergic receptors are involved in the regulation of settlement and metamorphosis in oysters [20, 32]. In 2012, molecular characterization and functional analysis of adrenergic-like receptors during larval metamorphosis in *Crassostrea angulata* was studied by our research group [33].

Recently, many studies of the molecular mechanisms of metamorphosis have used sequencing techniques, transcriptome, and genomic data analysis [34]. However, genetic data are unable to reveal the complete molecular mechanisms [35], because there is no direct correlation between gene expression intensity and protein abundance [36]. Proteomic analysis is a crucial approach for studies of bivalve oyster metamorphosis [35].

Proteomics analysis has been carried out on multiple marine genera during their settlement and metamorphosis stages [37–42]. As such, proteomic analysis has become a powerful tool to investigate the molecular mechanisms involved during different stages of development of marine invertebrates, and the method has been employed in embryonic developmental stages of several mollusks, including larvae [35, 36, 43, 44] reproduction [45], attachment, and metamorphosis [46–50]. These studies provide comprehensive information on the molecular mechanisms involved. Proteomic analysis is an appropriate approach to study larval settlement and metamorphosis of *C. angulata*.

Label-free quantification proteomics involves the application of a label-free quantification algorithm used in Maxquant for proteomics data. This technique is rapid, clean, and simple. The method can directly and precisely quantify protein expression without using labeling, and it has been broadly applied to biomarker discovery and proteomic profiling [51]. In this study, label-free proteomic analysis was carried out on larvae of the oyster *C. angulata* that had been induced by epinephrine during settlement and metamorphosis. The collected data were analysed using Scaffold Proteomics Software with database [52]. In addition, gene function annotation, GO enrichment, KEGG enrichment, and transcriptome and proteome combined analysis and qPCR verification were carried out. The results provide insights into the different protein groupings active during energy metabolism, shell formation, attachment and metamorphosis, and immune-related activities in *C. angulata*.

## Results

### Identification and quantitative results

In this study, a total of 412,946 spectra were identified (Supplementary Fig. 1), with 137,372 unique specta, 102,696 peptide fragments, 60,245 unique peptide fragments, and 1471 proteins. The relative molecular weights of the identified proteins were mainly distributed in the range of 10–60 kDa, of which 20–30 kDa was the largest group (Supplementary Fig. 2A), accounting for 17.3%; Supplementary Fig. 2B showed that the length of the identified peptide segments was mainly distributed in 10–18 kDa, of which 11–14 kDa was the largest group, accounting for 35.0%. Supplementary Fig. 2C illustrates the coverage of the identified peptide segments, in which 49.4% coverage was more than 20%. Supplementary Fig. 2D shows that a number of identified proteins contained only one or two peptide segments, and three peptide segments accounted for 48.7%.

### Identification and analysis of differentially expressed proteins (DEPs)

**Fig. 1:**
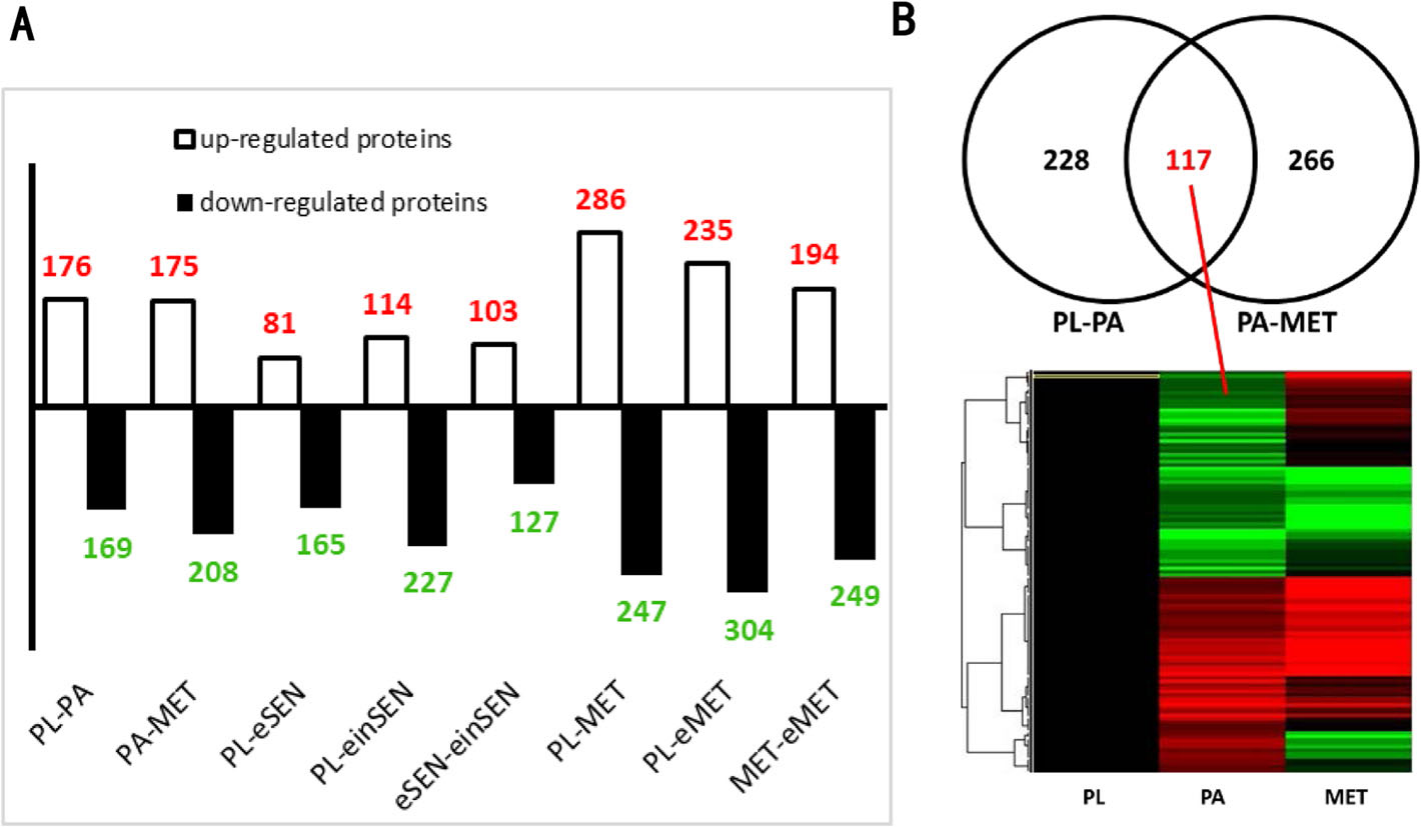
Basic information concerning different groups and the analysis of the PL-PA-MET groups regarding DEPs

Protein abundances with at least 1.5-fold change among the groups were compared and considered as up-regulated or down-regulated (Fig. 1a). A total of 611 proteins were differentially expressed in at least one larval stage among the PL-PA-MET process. There were 117 co-expressed proteins in the PL-PA and PA-MET groups (Fig. 1 b). The results of a cluster analysis showed that expression levels of most proteins had a consistent trend in the PL-PA-MET developmental process (Fig. 1 b).

**Fig. 2:**
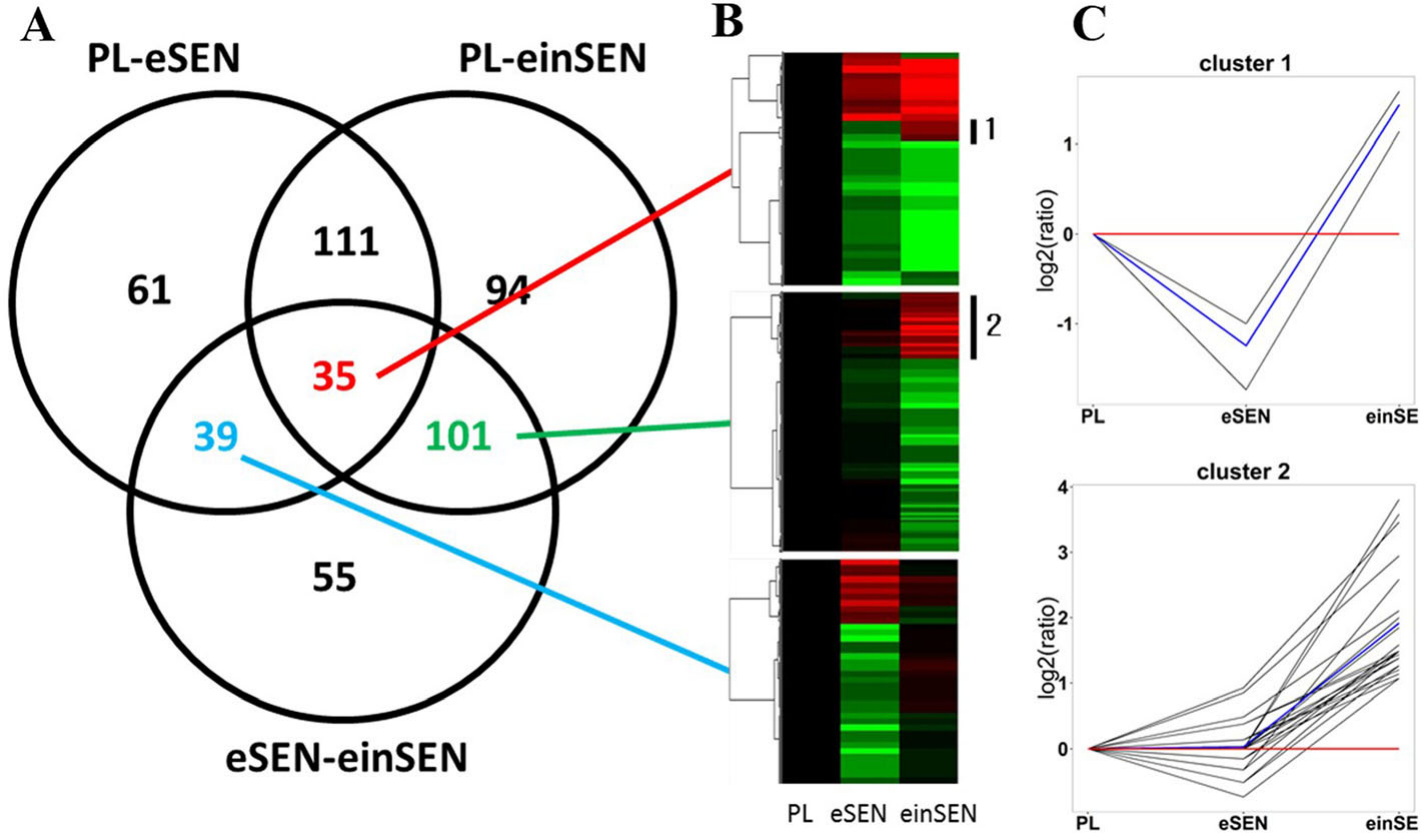
Summary analysis of differentially expressed proteins in the PL-eSEN-einSEN groups

There were 246, 341, and 230 DEPs in the PL-eSEN group, the PL-einSEN group, and the eSEN-einSEN groups, respectively (Fig. 1a). There were 35 DEPs present in all 3 groups, of which 22 were down-regulated in the PL-eSEN and PL-einSEN groups, and 3 proteins were down-regulated in the eSEN group (Fig. 2a) and up-regulated in the einSEN group (Cluster 1 of Fig. 2c). A total of 101 proteins were differentially expressed only in the PL-einSEN group and eSEN-einSEN group (Fig. 2a-b); there were 26 proteins that were down-regulated in the eSEN group but up-regulated in the einSEN group. The trend of expression is shown in cluster 2 of Fig. 2c.

**Fig. 3:**
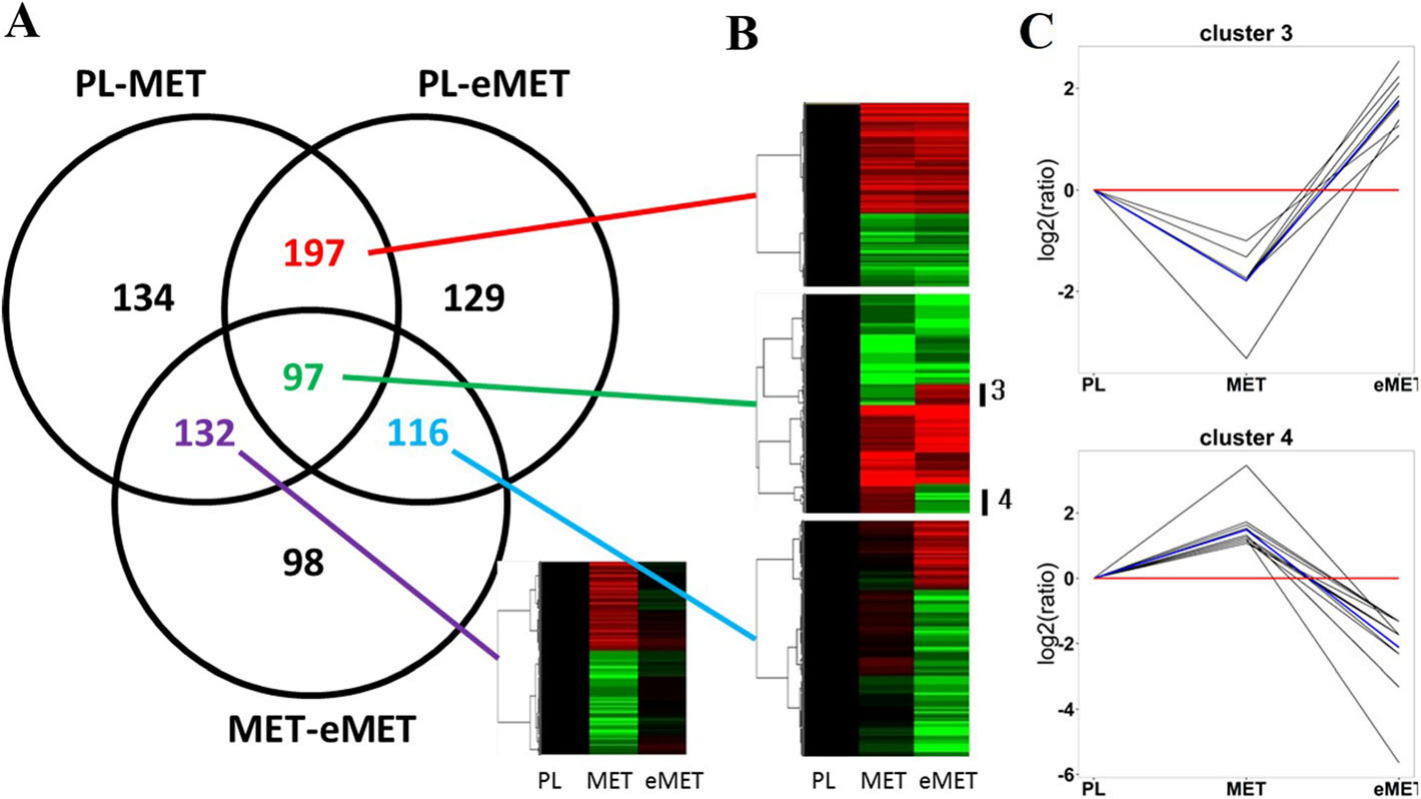
The summary analysis of differentially expressed proteins in the PL-MET-eMET groups

There were 560, 539, and 443 DEPs in the PL-MET, PL-eMET and MET-eMET groups, respectively, of which 286, 235, and 194 proteins were up-regulated and 274, 304, and 249 proteins were down-regulated, respectively (Fig. 1a). There were 97 DEPs common to all three groups (Fig. 3a). Among these, nine proteins were down-regulated in MET and up-regulated in eMET. The trend of expression is shown in cluster 3 of Fig. 3c, while 13 proteins were up-regulated in MET and down-regulated in eMET, as shown in cluster 4 of Fig. 3c. There were 132 DEPs observed only in the PL-MET and MET-eMET groups, and 116 DEPs found only in the PL-eMET and MET-eMET group (Fig. 3a). In addition, 197 proteins were differentially expressed in the PL-MET and PL-eMET groups but not in the MET-eMET group. Among these, 118 of the up-regulated proteins may be related to metamorphosis.

**Fig. 4:**
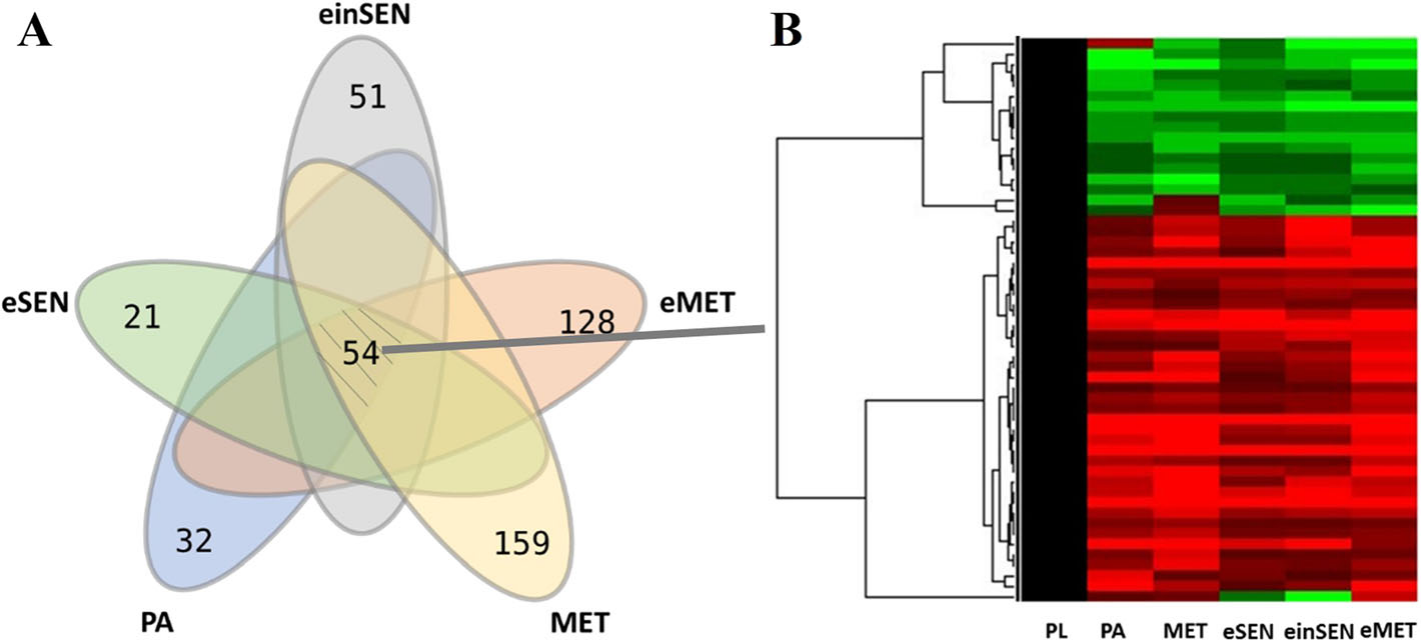
The summary analysis of differentially expressed proteins among all groups

Figure 4a displays the Venn diagram of DEPs in five groups, with the PL group as a control group. A total of 954 proteins were differentially expressed in at least one group. Interestingly, 54 proteins were differentially expressed in all five of the groups. The hierarchical cluster analysis (Fig. 4b) showed that the expression trends of most proteins were basically similar. In addition, the PL-MET and PL-eMET groups had the highest numbers of specific differentially expressed proteins, 159 and 126 proteins, respectively, and the PL-eSEN group had the least number, only 21 differentially expressed proteins.

Table 1 shows some DEPs, including long chain fatty acid CoA ligase (ACSBG), calnexin, and glutathione S-transferase.

**Table 1:** Some differentially expressed proteins of proteome

Gene no.	protein name	Swissprot ID	Groups
c96149_g1	Long chain fatty acid CoA ligase ACSBG	Q7ZYC4	1,2,3,4,6
c97263_g1	calnexin	P24643	1,2,6
c83840_g1	glutathione S-transferase	P30568	1,3,4
c96209_g1	ryanodine receptor 2	Q24498	1,3,4
c98073_g1	RAC serine/threonine-protein kinase	Q8INB9	1,4,5,6
c89544_g1	protein kinase A	P12370	1,4,5
c95819_g1	long-chain-acyl-CoA dehydrogenase	P51174	1,6
c92890_g1	Ras-related protein Rap-1A	Q640R7	1
c86623_g1	growth factor receptor-binding protein 2	Q60631	2,3,4,5
c86876_g1	20S proteasome subunit beta 7	P28024	2,6
c91059_g3	chitinase	Q9BZP6	2,6
c99122_g1	aldehyde dehydrogenase (NADP^+^)	Q5XI42	3,4,5
c97912_g1	mitogen-activated protein kinase 1/3	P26696	4
c96333_g1	isocitrate dehydrogenase	Q9Z2K9	6
c87236_g1	maleylacetoacetate isomerase	Q9WVL0	6
c96614_g1	solute carrier family 8	P70549	5
c90267_g1	phosphoenolpyruvate carboxykinase	P05153	1,3,4
c84601_g1	guanine nucleotide-binding protein Gsubunit alpha	P30683	1,2,3,4
c90479_g1	Ca^2+^ transporting ATPase, sarcoplasmic/endoplasmic reticulum	Q7PPA5	1,2,4,5,6
c98992_g1	tyrosine-protein phosphatase non-receptor type 11	Q90687	2
c100493_g1	guanine nucleotide-binding protein G subunit alpha	P30682	2

Note: The number 1 represented PL-PA group, the number of PA-MET group, PL-eSEN group, PL-einSEN group, eSEN-einSEN group and MET-eMET group were 2, 3, 4, 5 and 6, respectively

### Analysis of the significant differences in protein abundance

Compared with PL and PA groups (the larvae before or after settlement), cadherin-23(GI 405960423, GI 762110068), cadherin-87(GI 762110074), calreticulin (GI 150404776), cathepsin L (GI 405966500, GI 762107740, GI762167480), mucin-like protein (GI 762100460), kyphoscoliosis peptidase (GI 405972462), radial spoke head 1 homolog (GI 762085934), SCO-spondin (GI 405966926, GI 675373238), V-type proton ATPase subunit S1-like (GI 762145704) were higher expression in PA group. Meanwhile, calcium/calmodulin-dependent protein kinase type II delta chain (GI 405964165), calcium-binding mitochondrial carrier protein Aralar1 (GI405969211), calcium-transporting ATPase (GI 405968450, GI 405968450), calmodulin (GI20137620, GI762161385), calnexin (GI 405967580), calumenin-like isoform X1 (GI 762104881), ryanodine receptor 44F(GI 307197748), cilia- and flagella-associated protein 20 (GI 762102752), hepatic lectin-like (GI 762109657), MAP kinase-activated protein kinase 2-like (GI762156704), peroxisomal multifunctional enzyme type 2 (GI405977917, GI 762084138), Ras-like GTP-binding protein RHO (GI333449487), Rab3 (GI 762144541), Rab-10 (GI 405976260), Rab-14(GI 762121318), Rab-35 (GI 762141921), Rho GTPase-activating protein 17(GI 405978849), superoxide dismutase [Mn] (GI 821595281), universal stress protein A (GI 762094049,762,091,580, 762,091,586, 762,091,592) were lower expression in PA group (Supplementary Table 1).

Compared with PA and MET groups (the larvae before or after metamorphosis), cadherin-23, calcium-binding mitochondrial carrier protein Aralar1, calcium-transporting ATPase (GI405968450,762,086,942), calmodulin (GI20137620,762,161,385), calnexin, caltractin-like, calumenin-like isoform X1(GI762104881,762,079,826), integrin alpha-6, integrin beta-1, collagen alpha (GI405954419, 405,961,982), HEAT repeat-containing protein 7A, heat shock 70 kDa protein 14-like, heat shock protein 27-like, cathepsin L1-like, IgGFc-binding protein-like, interferon-induced protein 44-like, mucin-5 AC-like (GI762099838, 762,076,941), neural-cadherin-like, neurexin-4, neuroglian (405,960,111, 405,958,312), ran-specific GTPase-activating protein-like, Ras-like GTP-binding protein RHO, Rho GTPase-activating protein 17 were higher expression in MET group. Meanwhile, apoptosis-inducing factor 3-like isoform X1, calcium/calmodulin-dependent protein kinase type II delta chain, calpain-7-like protein, catalase, chitinase-3-like protein 1 isoform X3, cilia-and flagella-associated protein (GI762102752, 762,156,177), kyphoscoliosis peptidase, radial spoke head protein (GI762085934, 762,119,151,762,133,006), ryanodine receptor 44F, mucin-19-like, mucin-like protein, mitogen-activated protein kinase 1-like, peroxisomal multifunctional enzyme type 2 (GI405977917, 762,084,138), superoxide dismutase [Mn], Rab-14, soma ferritin-like were lower expression in MET group (Supplementary Table 2).

Compared with PA and eSEN groups, calcium-binding mitochondrial carrier protein Aralar1, calcium-transporting ATPase (GI405968450,762,086,942), calmodulin-like, calumenin-like isoform X1, neurexin-4, neuroglian, ryanodine receptor 44F, MAP kinase-activated protein kinase 2-like, Ras-like GTP-binding protein RHO, Rab-35-like, Rho GTPase-activating protein 17, superoxide dismutase [Mn], universal stress protein A-like protein (GI762091580, 762,094,049) were higher expression in eSEN group. Meanwhile, apoptosis-inducing factor 3-like isoform X1, caltractin-like, integrin alpha-8, integrin beta pat-3-like, 14–3-3 protein zeta, SCO-spondin (GI405973590,405,966,926), radial spoke head protein 4 homolog A-like, neural-cadherin-like, neurogenic locus Notch protein, neuroglian, neutral ceramidase-like, kyphoscoliosis peptidase, mucin-2-like, mucin-5 AC-like (GI871278596, 762,076,941,762,099,838), mucin-like protein, vinculin-like isoform X7, peroxisomal multifunctional enzyme type 2-like, cathepsin L, cathepsin L1-like (GI762167480, 762,099,884, 762,107,740), heat shock protein 27-like, Ran GTPase-activating protein 1, soma ferritin-like, V-type proton ATPase subunit S1-like were lower expression in eSEN group (Supplementary Table 3).

Compared with MET and eMET groups, apoptosis-inducing factor 3-like isoform X1, calcium/calmodulin-dependent protein kinase type II delta chain, calpain-7-like protein, EF-hand calcium-binding domain-containing protein (GI762115410, 762,133,698, 405,963,739), chitinase-3-like protein 1 isoform X3, cilia- and flagella-associated protein 61-like, mucin-17-like, mucin-19-like, mucin-5 AC-like, mucin-like protein, 14–3-3 protein zeta, SCO-spondin (GI405973590, 405,973,589, 675,373,238), radial spoke head protein (GI762085934, 762,084,810,762,119,151), neural-cadherin-like, neurogenic locus Notch protein, peroxiredoxin-5, peroxisomal multifunctional enzyme type 2 (GI405977917, 762,084,138), catalase, superoxide dismutase [Mn], mitogen-activated protein kinase 1-like, soma ferritin-like, universal stress protein A-like protein were higher expression in eMET group. Meanwhile, calcium uniporter protein, calcium-binding mitochondrial carrier protein Aralar1, calcium-transporting ATPase, calmodulin, calnexin, calumenin-like isoform X1, cathepsin L1-like, collagen alpha, drebrin-like protein B isoform X2, laminin subunit alpha (GI405969732,762,109,423), laminin subunit gamma-1, vinculin-like isoform X7, lethal (2) giant larvae protein (GI 762141840, 405,952,027), neurexin-4, neuroglian, neuroglian-like isoform X1, neutral ceramidase-like, HEAT repeat-containing protein 7A, heat shock 70 kDa protein 12B, heat shock 70 kDa protein 14-like, heat shock protein 27-like, Ras-like GTP-binding protein RHO, ras-like protein 3 isoform X2, Rab-7a, Rho GTPase-activating protein 17, V-type proton ATPase subunit H-like isoform X3 were lower expression in eMETgroup (Supplementary Table 4).

The differentially expressed proteins with important physiological functions among six groups along with calculated protein volumes are shown in Supplementary Table 5. For statistical analysis, the expression levels of proteins with the same name were merged. The results are shown in Supplementary Table 6.

#### Energy metabolism proteins

In present study, we identified several energy metabolism proteins. Among them, ATP synthase, D-3-phosphoglycerate dehydrogenase-like, enolase and malate dehydrogenase were not statistically different among the six groups. 6-phosphogluconate dehydrogenase, decarboxylating, glucose-6-phosphate 1-dehydrogenase, glucose-6-phosphate isomerase-like, succinate dehydrogenase, pyruvate dehydrogenase E1 component subunit alpha type II, mannose-6-phosphate isomerase-like, citrate synthase and mitochondrial-like isoform X1 were up-regulated in PL group and down-regulated in MET. Pyridoxal-dependent decarboxylase domain-containing protein 1 was up-regulated in MET. Most proteins involved in carbohydrate metabolic processes were up-regulated in PL group and down-regulated in MET group. (Supplementary Table 6).

### Immune proteins

In this study, a variety of immune proteins were identified. Among them, cathepsin Z, cathepsin L, Hsp60, Hsp70, Hsp27 and IgGFc-binding protein-like were up-regulated in MET, these proteins were also highly expressed in eMET. Hepatic lectin-like and Cu/Zn-SOD -like isoform X1 were up-regulated in eMET, these proteins were also highly expressed in MET. On the contrary, galectin-6, glutathione-S-transferase, universal stress protein A-like protein, galectin-9-like isoform X2 and Mn–SOD were down-regulated in eMET; universal stress protein A-like protein, galectin-9-like isoform X2 and Mn–SOD also were down-regulated in MET (Supplementary Table 6).

#### Shell formation proteins

In this study, cadherin, calmodulin, calreticulin, caltractin-like and calumenin-like isoform X2 were up-regulated in MET and eMET groups; CaM kinase was down-regulated in MET and eMET groups; calcium uniporter protein was down-regulated in eMET group; Calnexin was up-regulated in MET, down-regulated in eMET group; EF-hand calcium-binding domain-containing protein was down-regulated in MET group, up-regulated in eMET group; Troponin T was up-regulated in eMET group. The diversity of expression patterns also indicates the complexity of shell formation regulation.

Adrenergic signaling in cardiomyocyte: Several adrenergic signaling proteins were identified including 14–3-3 protein zeta, calcium/calmodulin-dependent protein kinase type II, calmodulin, cAMP-dependent protein kinase, guanine nucleotide-binding protein G(i) subunit, G(q) subunit and G(s) subunit alpha, mitogen-activated protein kinase 1-like, myosin heavy chain, paramyosin-like isoform X3, RAC-gamma serine/threonine-protein kinase-like, ryanodine receptor 44F, serine/threonine-protein phosphatase, sodium/potassium-transporting ATPase, tropomyosin isoform X2 (Supplementary Table 5, 6). In this study, 14–3-3 protein zeta was up-regulated in MET and eMET groups; calmodulin and calumenin-like isoform X2 were up-regulated in MET and eMET groups (Supplementary Table 6); mitogen-activated protein kinase 1-like and ryanodine receptor 44F were down-regulated in MET and eMETgroups; serine/threonine-protein phosphatase 2A 65 kDa regulatory subunit A and catalytic subunit beta were down-regulated in MET group (Supplementary Table 5).

### GO annotation results

All of the identified proteins were annotated by GO. A total of 11,028 proteins were annotated, accounting for 55.1% of the total identified proteins. Among these, 55 secondary GO terms were classified (Supplementary Fig. 3). From a total of 51,618 nodes, 18,814 were involved in biological processes, mainly in cell processes and metabolic processes, with 5405 and 5247 nodes accounting for 28.7 and 27.9% of the total GO term nodes, respectively. For cellular components, there were 20,849 nodes, mainly distributed in cell and cell part classifications. In the molecular function classification, there were 11,955 nodes, mainly involving two types of GO terms: binding and catalytic activity, with 5718 and 4547 nodes, accounting for 47.8 and 38.0%, respectively.

### KEGG annotation results

The identified proteins were annotated to KO according to NR Gene ID. A total of 5742 proteins were annotated, accounting for 28.7%. In primary pathway classification, the number of proteins participating in metabolism, genetic information processing, environmental information processing, cellular processes and organismal system were 1258, 630, 996, 660, and 1529 proteins, respectively (Supplementary Fig. 4), accounting for 24.8, 12.4, 19.6, 13, and 30.1%, respectively. There were 32 secondary KO classifications (Supplementary Fig. 4). The pathway annotated to the signal transduction was the largest group, with 877 proteins accounting for 17.3%, followed by endocrine system with 435 protein annotations, accounting for 8.6%. The numbers of proteins participating in immune system, carbohydrate metabolism, nervous system and amino acid metabolism were 289, 279, 278, and 269 proteins, accounting for 57.0, 55.0, 54.8, and 53.0% of the total annotated proteins, respectively.

### GO enrichment analysis of DEPs

For GO significance analysis of DEPs, a *P-*value < 0.05 and FDR < 0.05 were used as a threshold to select significant GO terms. Table 2 shows the GO enrichment in all of the groups; Supplementary Table 7 shows the extremely significant GO enrichment in the PL-PA and PA-MET groups. In PL-PA and PA-MET groups, 21 GO terms were extremely significantly enriched, including 14 GO terms in biological processes, 4 GO terms in cell components, and 3 GO terms in molecular functions (Supplementary Table 7).

**Table 2:** Summary of GO enrichment of DEPs

GO term	PL-PA group	PA-MET	PL-eSEN	PL-einSEN	eSEN-einSEN	PL-MET	PL-eMET	MET-eMET
**Total**	44/200 (95)	64/232 (109)	43/198 (76)	47/211 (106)	44/95 (64)	70/157 (106)	55/165 (111)	38/164 (83)
**Biological process**	23/111 (49)	33/136 (62)	19/127 (44)	24/117 (50)	18/40 (29)	37/81 (60)	28/84 (62)	20/99 (48)
**Cellular component**	6/30 (22)	13/37 (32)	7/21 (18)	7/32 (28)	6/20 (18)	10/22 (22)	9/27 (26)	6/24 (22)
**Molecular function**	15/59 (55)	18/59 (55)	17/50 (39)	16/62 (54)	20/35 (34)	23/54 (49)	18/54 (52)	12/41 (39)

Note: A/B, A represented an extremely significant enrichment of GO term; B represented a significant enrichment of GO term; “()” represented the number of DEPs

The statistical analysis of GO enrichment in the PL-eSEN, PL-einSEN, and eSEN-einSEN groups is showed in Table 2. The number of DEPs with significantly enriched GO terms in the PL-einSEN group was the largest, while the eSEN-einSEN group had the least number of DEPs and the least significantly enriched GO terms. Supplementary Table 8 shows that seven extremely significant GO terms were only expressed in the PL-eSEN and eSEN-einSEN groups; these were regulation of cell adhesion, histone lysine methylation, F-actin capping protein complex, neuropeptide hormone activity, dimethylallyltranstransferase activity, chaperone binding, and peptide-methionine (R)-S-oxide reductase activity. GO entries that were extremely expressed only in the PL-einSEN and eSEN-einSEN groups are shown in Supplementary Table 8.

The statistical analysis of GO enrichment in PL-MET, PL-eMET, and MET-eMET groups is shown in Table 2. The significantly enriched and extremely significantly enriched GO terms were involved in the molecular process among three groups; the most extremely significant GO terms were in the PL-MET group, which had 37 terms. Supplementary Table 9 shows that GO terms that were extremely significant only in the PL-MET and MET-eMET groups, only in the PL-eMET and MET-eMET groups, and only in the PL-MET and PL-eMET groups.

In addition, with PL as a control group, precorrin-2 dehydrogenase activity was the only extremely significantly enriched GO term for molecular functions in all five of the groups.

### KEGG enrichment analysis of DEPs

**Fig. 5:**
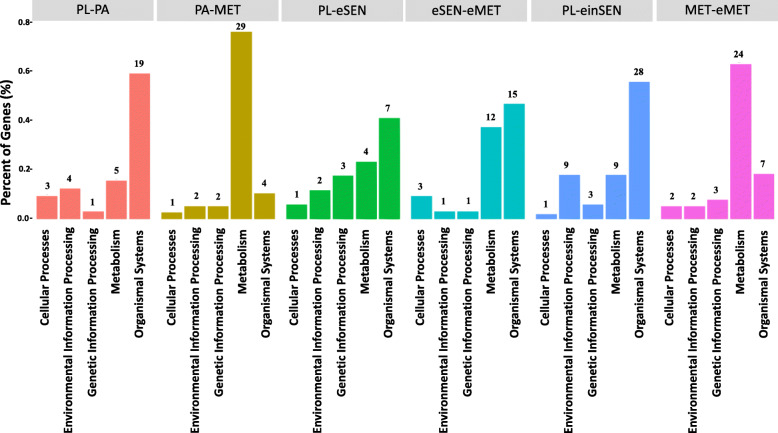
KEGG function classification among different groups

*P* < 0.05 was used as a determiner of significant enrichment in the KEGG pathway analysis. Pathways were classified in the PL-PA group, the PA-MET group, the PL-eSEN group, the PL-einSEN group, the eSEN-eMET group, and the MET-eMET group. The results are shown in Fig. 5.

During the process of oyster pediveliger settlement and metamorphosis, 22 DEPs were significantly enriched in 32 pathways in the PL-PA group, and these were mainly involved in organismal systems pathways (Fig. 5); 23 DEPs were significantly enriched in 38 Pathways in the PL-MET group, 29 of which were related to metabolism (Fig. 5) (Supplementary Table 10). The pathways related to carbohydrate metabolism included propanoate metabolism, citrate cycle (TCA cycle), butanoate metabolism, and pentose and glucuronate interconversion. The pathways related to lipid metabolism included fatty acid elongation, sphingolipid metabolism, biosynthesis of unsaturated fatty acids, fatty acid degradation, and primary bile acid biosynthesis (Supplementary Table 10). In addition, there were eight significantly enrichment pathways in both PL-PA and PL-MET groups.

From the pediveliger of the oyster to post-epinephrine-induced metamorphosis, in the PL-eSEN group, 14 DEPs were enriched in 17 pathways, of which 7 were related to organismal systems, and 4 were related to metabolism (Fig. 5). Compared with PL-eSEN group, 15 of 32 DEPs in the eSEN-eMET group were related to organismal systems, and 12 were related to metabolism. There were 12 unique pathways related to organismal systems in the eSEN-eMET group (Supplementary Table 11), including adrenergic signaling in cardiomyocytes, adipocytokine signaling pathway, and thyroid hormone synthesis; seven pathways were related to metabolism (Supplementary Table 11), including glycosphingolipid biosynthesis-ganglio series, alanine, and aspartate and glutamate metabolism. In addition, there were six pathways with significant enrichment in both the PL-eSEN and eSEN-eMET groups.

Twenty-three DEPs were significantly enriched in 50 pathways in the PL-einSEN group. Twenty-eight were related to organismal systems pathways (Fig. 5). Twenty-five were unique to the PL-einSEN group (Supplementary Table 12). Many of the DEPs were related to hormones or neurotransmitters, including insulin signaling pathway, adrenergic signaling in cardiomyocytes, GnRH signaling pathway, cholinergic synapse, dopaminergic synapse, and other pathways (Supplementary Table 12). The identified proteins involved in adrenergic signaling in cardiomyocytes are shown in Table 3. In the MET-eMET group, 26 DEPs were enriched in 38 pathways, 7 of which were related to organismal systems pathways. Twenty-four were related to metabolic pathways, of which 10 were related to carbohydrates and lipids, accounting for 41.7% (Fig. 5).

**Table 3:** The identified proteins involved in adrenergic signaling in cardiomyocytes

Protein	PL	eSEN	einSEN
Calmodulin	1	0.3	0.3
Ryanodine receptor	1	0.5	0.5
Calcium/calmodulin-dependent protein kinase	1	0.09	0.2
mitogen-activated protein kinase	1	0.8	0.5
cAMP-dependent protein kinase	1	0.8	0.3
serine/threonine-protein phosphatase	1	1.2	0.4
paramyosin-like isoform	1	1.3	2.7
serine/threonine-protein kinase	1	1.3	2.8

### mRNA expression of six differentially expressed genes

**Fig. 6:**
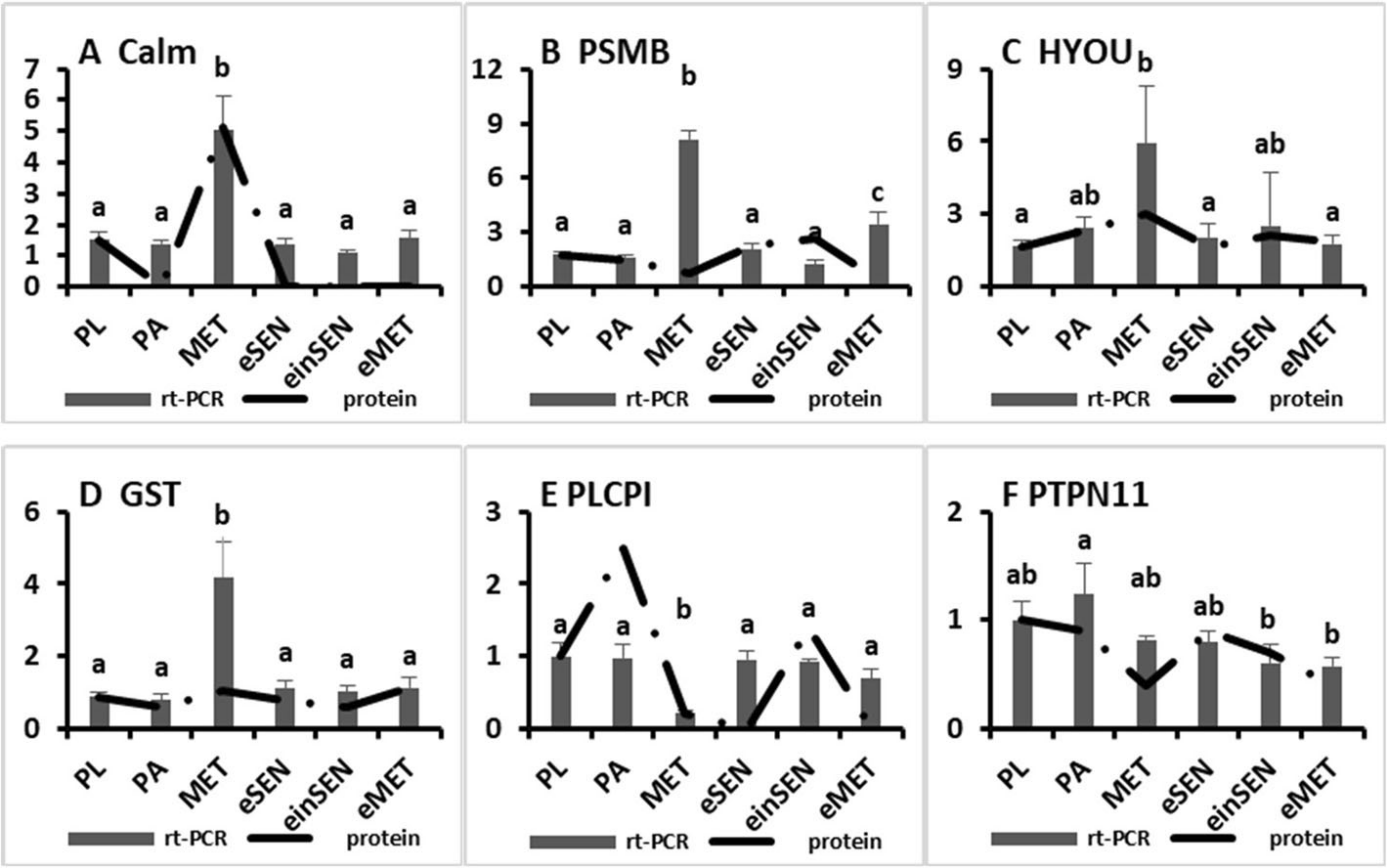
Comparison of mRNA and protein expression levels for six genes

To investigate the transcriptional levels of identified proteins, six DEPs were chosen for qPCR verification (Fig. 6) (Calm-calmodulin-like; PSMB-proteasome subunit beta; HYOU-hypoxia up-regulated 1; GST-glutathione S-transferase omega; PLCPI-Leukocyte cysteine proteinase inhibitor 1; and PTPN11-tyrosine-protein phosphatase non-receptor type 11). Compared with the protein expression patterns, the mRNA expression of two of the six genes (Calm and PTPN11) exhibited similar patterns; three genes (PSMB, HYOU, and GST) were differentially expressed at the transcriptional level but showed no significant changes in the protein levels.

### Predicted interactions of identified DEPs

**Fig. 7:**
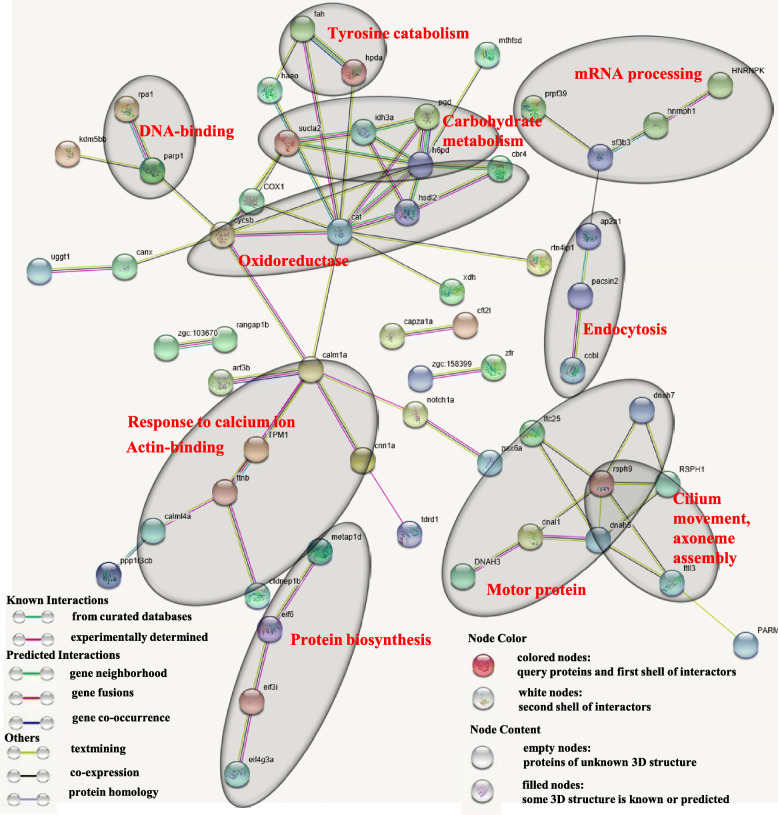
Predicted interactions of identified DEPs. Different line colours represent types of evidence for association. Major clusters associated with protein biosynthesis, motor protein, cilium movement, axoneme assembly, response to calcium ion, actin-binding, endocytosis, oxidoreductase, carbohydrate metabolism, mRNA processing, DNA-binding. Proteins without interactions have been removed from the graph. Protein abbreviations and corresponding full name are shown in supplementary Table 13

Figure 7 was obtained from the http://string.embl.de/ website and shows the predicted interactions of the identified DEPs. Major clusters were associated with protein biosynthesis, motor protein, cilium movement, axoneme assembly, response to calcium ion, actin-binding, endocytosis, oxidoreductase, carbohydrate metabolism, mRNA processing, and DNA-binding (Supplementary Table 13).

## Discussion

Studying the biological process of metamorphosis is the key to understanding the origin and evolution of mollusk development. The oyster transforms from a pelagic larva to a benthic adult, during which settlement and metamorphosis occur. Larvae undergo dramatic tissue transition [32], and the transition usually happens rapidly [9]. Some larval tissues degenerate during settlement and metamorphosis, e.g., the anterior adductor, the ciliated velum, and the ventral retractors [53]. The transition initiates histogenesis of some adult tissues (e.g., calcified shells and gill) as well as the formation of eyespot larvae (pediveliger)-specific tissues (e.g., eyespot and foot) during settlement and metamorphosis. As the oyster adult body plan is distinct from the larva, understanding how the molecular regulation is reorganized is crucial to gaining insight into the complexity of the transition [54].

In this study, a total of 1471 proteins were identified and quantified. Quantitative results showed that 764 DEPs were related to the larval developmental processes of settlement and metamorphosis, and 683 DEPs were related to larval development process of direct metamorphosis induced by epinephrine. Of the latter, there were 417 DEPs present in both the PL-PA-MET and PL-eSEN-eMET groups.

### Comparison of eSEN (EPI-sensitive larvae) and einSEN (EPI -insensitive larvae) groups

There were 246 DEPs in the PL-eSEN group; the number of DEPs in the PL-einSEN group was nearly one-third more, at about 341 proteins. In this study, the larvae that were not successfully induced displayed an excited planktonic state after the epinephrine-inducing fluid was replaced, while the larvae that were successfully induced quietly sank to the bottom of the tank. This may be due to the different motion states between the PL-eSEN group and the PL-einSEN group, where the number of DEPs differed. In addition, down-regulated proteins accounted for 67.1 and 66.6% of the differentially expressed proteins in the PL-eSEN group and PL-einSEN group, respectively. Most of the DEPs in this experiment were down-regulated. The reasons for this may have been as follows: firstly, the result may have been related to sampling. All of the larvae contracted their foot and velum rapidly when epinephrine was added, then closed their shells and sank to the bottom. At this stage, only the rotation of the visceral mass could be observed under the microscope, and most of the larvae remained in a dormant-like state for a long time. The results suggested that most of the physiological processes had slowed down or stopped. In this study, the epinephrine treatment time was only for 2 h. It is possible that larvae could not express new proteins necessary to complete the life cycle in such a short time.

The number of significantly enriched KEGG pathways in the PL-einSEN group was three times that in the PL-eSEN group. There were 50 significantly enriched pathways, including cytochrome P450-mediated substance metabolism, physiological rhythm transformation, extracellular matrix receptor function, and endoplasmic reticulum protein processing, present in both the PL-eSEN and PL-einSEN groups. Cytochrome P450 family was observed with a total of 491 genes belonging to P450 family members in pearl oyster [55]. Metabolism mediated by cytochrome P450 enzymes can decompose lipophilic toxic substances into aqueous solution [56], and this was up-regulated in eSEN and down-regulated in the einSEN group.

In addition, all of the proteins enriched in this functional module were down-regulated, including carbonyl reductase 1 (CBR 1), aldehyde dehydrogenase (E 1.2.1.5), and glutathione S-transferases (GSTs); the detoxification function of glutathione S-transferases has been widely studied [57]. In this study, compared with the PL group, the expression levels of GSTs were low in the einSEN and eSEN groups (Supplementary Table 6). This result suggested that epinephrine treatment for 2 h was not toxic to larvae, so proteins related to detoxification were down-regulated.

The unique signaling pathways in the PL-einSEN group included adrenergic signaling in cardiomyocytes, dopaminergic synapse, insulin signaling pathway, and cholinergic synapse (Supplementary Table 12). The expression trend of most DEPs enriched in these pathways was down-regulation; there were eight DEPs in the adrenergic signaling in cardiomyocytes, among which six were down-regulated, including serine/threonine-protein phosphatase, cAMP-dependent protein kinase (PKA), mitogen-activated protein kinase (MAPK), calcium/calmodulin-dependent protein kinase II (CAMK 2), ryanodine receptor, and calmodulin (Table 3). Although the expression of these proteins was down-regulated in the eSEN group, only three proteins (calmodulin, ryanodine receptor, calcium/calmodulin-dependent protein kinase) were differentially expressed (Table 3). We speculated that the expression levels of proteins related to the epinephrine pathway in the larvae of the eSEN group were higher than in the einSEN group before epinephrine treatment. After the same epinephrine stimulation, although the down-regulation of differential proteins in the two groups was the same, the expression levels of proteins in the eSEN group were higher, so the einSEN group showed more DEPs. In this study, the proteins related to the epinephrine pathway were at higher expression levels in pediveligers (PL), and the expression of those proteins may reflect the developmental stage of the larvae, as this stage was directly involved in metamorphosis when the larvae were treated with epinephrine.

### Proteins involved in energy metabolism in settlement and metamorphosis

There were 29 KEGG enrichment results related to metabolic pathways in the PL-MET group, including carbohydrate and lipid metabolism. The pathways related to carbohydrate metabolism included propanoate metabolism, citrate cycle (TCA cycle), butanoate metabolism, and pentose and glucuronate interconversions. The pathways related to lipid metabolism included fatty acid elongation, sphingolipid metabolism, biosynthesis of unsaturated fatty acids, fatty acid degradation, and primary bile acid biosynthesis (Supplementary Table 10). First, the metabolic processes can generate energy to ensure the normal development of oysters while also providing the material needed for oysters’ development, as the processes of settlement and metamorphosis involve changes in organs and tissues.

Carbohydrates and lipids provide energy and nutrients for embryonic development [58]. Isocitrate dehydrogenase is involved in energy homeostasis, and this was down-regulated in eMET (Supplementary Table 6). ATP synthase is needed for cellular energy interconversion. Enolase is a metalloenzyme responsible for the catalysis of the conversion of 2-phosphoglycerate to phosphoenolpyruvate in glycolysis. Glycolysis-related proteins play a key role in the embryonic development of zebrafish and ascidians [59]. Most proteins involved in carbohydrate metabolic processes were up-regulated in the PL group and down-regulated in the MET group (Supplementary Table 6). These results suggested that the metabolism of larvae was restored to a stable state after settlement and metamorphosis.

The KEGG enrichment of the MET-eMET group showed that KEGG pathways were mainly distributed in metabolic processes and organism systems. Lipid metabolism and polysaccharide synthesis and metabolism were the main metabolic pathways, including fatty acid elongation, fatty acid degradation, sphingomyelin metabolism, biosynthesis of unsaturated fatty acids, carbon metabolism, and glycosphingolipid biosynthesis. Jensen et al. [60] reported artificial induction of larval metamorphosis by free fatty acids in the polychaete *Phragmatopoma californica*, suggesting that free fatty acids induce larval metamorphosis by operating physiologically downstream or parallel to the natural inducer. The results indicated that the metamorphosis process of oysters requires significant energy consumption and that lipid metabolism is involved in larval metamorphosis.

### Proteins involved in settlement between PL and PA groups

In marine invertebrates, the velum disappears gradually from planktonic life at the early veliger stage to the benthic form as the larvae undergo the attachment process. In 1996, Clare reported that a low concentration of Ca^2+^ inhibited the settlement of Barnacle larvae [61]. Yamamoto et al. [62] and Zhang et al. [63] found that calmodulin (CaM) and calmodulin-determined myosin light chain kinase (MLCK) played important roles in the settlement process of barnacles. Zhang et al. [63] showed that CaM was highly expressed in cyprids in the swimming larvae that are competent to attach and undergo metamorphosis. In this study, in comparing the PL and PA groups, calcium/calmodulin-dependent protein kinase type II delta chain (GI 405964165) and calmodulin (GI20137620, GI762161385) were more highly expressed in the PL group. This result indicated that calmodulin was involved in the regulation of settlement of oysters.

Previous studies have found that the p38 MAPK pathway was activated during the settlement process of barnacles [64, 65]. In this study, protein ID c100749_g1 was identified as MAP kinase-activated protein kinase 2-like (Supplementary Table 5). In comparisons of the PL and PA groups, mitogen-activated protein kinase 2-like was expressed at a higher level in the PL group. The NOTCH signaling pathway was involved in the regulation of somatic division of *Capitella sp.* [66]. In this study, the expression levels of neurogenic locus Notch protein in the PL, MET and eMET groups were 0.00, 4.77 and 13.89, respectively. The protein was up-regulated in the MET and eMET groups (Supplementary Table 6). This result indicated that MAPK and NOTCH signaling pathways were also involved in the regulation of settlement and metamorphosis of oysters. The classical signal pathway closely related to cell proliferation and apoptosis, the mitogen-activated protein kinase (MAPK) pathway, has also been well studied. Those pathways are involved in the regulation of settlement and metamorphosis of marine invertebrates [48, 64, 65, 67].

### The expression levels of immune-related proteins increased in metamorphosis

Metamorphosis is the key stage when the expression levels of immune-related genes increase and respond to environmental stimuli [68]. Balseiro et al. [68] revealed that the expression levels of immune-related genes were higher than those in oocytes at this metamorphic stage in *Mytilus galloprovincialis.* The results suggested that immune-related genes were active in mussel larvae. Previous study reported up-regulation of innate immune-related genes during metamorphosis in the ascidian *Boltenia villosa* [69].

Due to the lack of adaptive immunity in mollusks, lectins act as determinants of phagocytosis [70]. Hsp70, as a member of the molecular chaperones, is a highly conserved stress protein that can protect cells from harmful assault. In addition, Hsps are involved in developmental processes [71]. Gunter et al. [71] found higher expression levels of Hsp70 in tissues undergoing larval morphogenesis in the marine gastropod *H. asinine*, similar to the patterns observed in ecdysozoans and deuterostomes [71]. Cu/Zn Superoxide dismutase (Cu/Zn-SOD) is closely related to immunity in mollusks. The enzyme can enhance the activity of phagocytic cells and the body’s immune function and protect the cells from ROS poisoning [72]. In this study, cathepsin Z-like, cathepsin L, 60 kDa heat shock protein, heat shock 70 kDa protein 14-like (GI762129389), heat shock protein 27-like and IgGFc-binding protein-like were up-regulated in MET; subsequently, these proteins were highly expressed in eMET (Supplementary Table 6). Hepatic lectin-like (GI762109657) and superoxide dismutase (Cu-Zn)-like isoform X1 were up-regulated in eMET; subsequently they were highly expressed in MET (Supplementary Table 6). These results might indicate maturation of the innate immune system and the correlation between the resorption and restructuring of larval tissues and the ability of larvae to detect and respond to bacterial settlement cues [68, 69]. Protein expression began to increase after the veliger stage in order to prepare the larvae for metamorphosis.

### Proteins involved in metamorphosis

Some previous studies of metamorphosis in marine organisms have suggested that lectins are involved in settlement, metamorphosis and tissue remodeling [48, 69, 73–75]. Maki and Mitchell [76] proposed a biochemical lectin model for settlement and metamorphosis of marine invertebrate larvae, and they demonstrated that lectin “recognizes” and binds to a glycoconjugate in the exopolymer of marine bacteria. Matsutani et al. [77] discussed how lectin-like factors were involved in larval settlement and metamorphosis in the abalone *H. discus hannai*. Bao et al. [78] suggested that a putative C-type lectin fold might play an important role during the metamorphosis of *Mizuhopecten yessoensis*. In this study, hepatic lectin-like (GI762109657) was up-regulated in the MET and eMET groups (Supplementary Table 6). These results suggested that hepatic lectin-like proteins are involved in the metamorphosis of *C. angulata*.

In this study, cadherins, calmodulin and calreticulin were up-regulated in the MET and eMET groups (Supplementary Table 6). Cadherins are a family of homophilic Ca^2+^-dependent cell adhesion molecules controlling animal morphogenesis [79]. Chen et al. (2012) found that calmodulin affected settlement and metamorphosis of *Hydroides elegans*. In this study, the results were consistent with those from a study by Chen et al. on the expression of calmodulin in the processes of settlement and metamorphosis of the polychaete *Hydroides elegans* [80]. This suggests that calmodulin may play an important role in oyster metamorphosis.

Comparing the PA and MET groups, apoptosis-inducing factor 3-like isoform X1 was expressed at a lower level in the MET group. Apoptosis has also been reported in marine invertebrate metamorphosis. The tail loss in *Ciona intestinalis* is regulated by an apoptosis-related factor during larval metamorphosis [81], suggesting the necessity of apoptosis for larval metamorphosis.

### Proteins associated with shell formation

Changes in shell formation are important in the development of mollusks. The oyster shell undergoes transformation during metamorphosis from an aragonite larval shell to the calcite adult shell [82]. In this study, calmodulin and calreticulin were up-regulated in the MET and eMET groups (Supplementary Table 6). Calmodulin and calreticulin were also identified in the larvae of the snail *P. canaliculata* during shell development [43] and in the Pacific oyster *C. gigas* [35]. It has been reported that calmodulin was involved in shell formation [83] and that it is associated with calcium signaling. The up-regulation of both proteins suggests that they may be involved in the calcification of larval shells. Calreticulin constitutes a cellular protein “quality-control” system [84] that is important for developmental processes.

In addition, calreticulin can bind to misfolded proteins, correcting the protein folding or directing the protein toward a degradation pathway. Regarding mRNA expression levels, compared with the protein expression patterns, the mRNA expression of calmodulin exhibited similar patterns (Fig. 6), suggesting that this gene was involved in shell formation. The proteins related to calcium metabolism may be involved in calcium deposition and are likely involved in the shell formation that occurs in larvae of *C. angulata*.

In this study, comparison of PA and MET group, collagen alpha (GI405954419, 405,961,982) was up-regulated in the MET group. In shell matrix, collagen-like VWA domain containing proteins, fibronectin-like proteins and laminins were involved in the regulation of shell formation [85, 86]. Chitin is also considered the base membrane in shell organic matrix [87]. In this study, comparison of MET and eMET group, chitinase-3-like protein 1 isoform X3 was up-regulated in the eMET group.

### Nervous system remodeling during metamorphosis induced by epinephrine

The oyster foot senses a suitable substratum for settlement, suggesting biogenesis of new nerve connections. As a signal of metamorphosis, the appearance of an eyespot indicates the possible remodelling of the oyster larval nervous system. In the present study, 14–3-3 and SCO-spondin were identified. Both proteins were significantly up-regulated in the eMET group (Supplementary Table 6). The 14–3-3 protein is involved in diverse physiological processes, including growth and development, receptor signal regulation, tumorigenesis, molecular chaperone, activation or inhibition of the target protein activity [88–90], and innate immunity in *Drosophila* [91]. In this study, there were no significant differences among PL, PA, and MET groups, but the 14–3-3 protein was up-regulated in the eMET group (Supplementary Table 6). The overexpression of 14–3-3 in the body plays an important role in nerve cell differentiation [92]. The 14–3-3 proteins participate in neurodevelopment, and 14–3-3ε and ζ are associated with neurogenesis and neuronal progenitors of cell differentiation in the developing brain [93]. The 14–3-3 proteins also play important roles in left–right patterning in the axial direction of the embryo during amphibian embryogenesis [94]. In the present study, SCO-spondin was expressed at a high level in the PA group and was up-regulated in the eMET group. SCO-spondin is needed for neurogenesis during early brain development, Vera et al. [95] revealed that SCO-spondin is a key embryonic cerebrospinal diffusible factor regulating the balance between proliferation and differentiation of the brain neuroepithelial cells. The result also suggested that 14–3-3 and SCO-spondin changed in larvae of *C. angulata* induced by epinephrine*.*

Calmodulin and the 14–3-3 gamma protein are activators of tryptophan 5-monooxygenase and tyrosine 3-monooxygenase [96, 97], which are important for the biosynthesis of serotonin, noradrenalin and epinephrine, neurotransmitters that are key in neuronal activities [98, 99]. Our study results suggested that the activation mechanisms of tryptophan 5-monooxygenase and tyrosine 3-monooxygenase in *C. angulata* might be similar to those of mammals [96, 97]. The up-regulation of 14–3-3 and calmodulin in the MET and eMET groups may indicate their roles in neuronal function and may indicate the enhanced development of the nervous system in *C. angulata*. This result is consistent with a report on the early larval development of the Pacific oyster *C. gigas* [63].

Epinephrine, dopamine, gamma-aminobutyric acid (GABA), and acetylcholine have been shown to be effective neuroendocrine compounds for inducing oyster metamorphosis [31]. In this study, neuronal acetylcholine receptor subunit alpha-10 (c88012_g1) and ryanodine receptor 44F (c96209_g1) were upregulated post epinephrine stimulation in the eSEN group. Neuronal acetylcholine receptor subunit alpha-10 is an agonist of binding that may affect all of the subunits and lead to opening of an ion-conducting channel across the plasma membrane. The channel is permeable to a range of divalent cations including calcium. One of the channels that mediates this release of Ca ^2+^ from the endoplasmic reticulum is the ryanodine receptor (RyR), which is essential for larval development in *Drosophila melanogaster* [100]. Functional assays of these key receptors will help to develop new strategies to control the settlement and metamorphosis of marine animals more effectively.

### Proteins associated with cytoskeletal proteins in metamorphosis

The expression levels of cytoskeletal proteins such as paxillin, myosin regulatory light chain (MYL), vinculin and caltractin were all up-regulated in the MET stage, and the expression levels of caltractin and paxillin in the MET group were 3.9 and 8 times higher than those in PL group, respectively (Supplementary Table 6). These results indicated that skeleton proteins had undergone drastic changes during metamorphosis. Up-regulation of skeleton proteins is often found in the study of settlement and metamorphosis of marine invertebrates [40, 41]. Studies have shown that skeleton proteins play an important role in the metamorphic development of amphibians [101] and insects [102]. Skeleton proteins can maintain cell structure and shape, and they provide support for cell movement, cell proliferation and cell growth. Differentially expressed proteins related to cytoskeleton regulation included integrin alpha L (ITGAL), actin-related protein 2/3 complex, subunit 5 (ARPC5) and gelsolin, which further reflected the drastic changes to the cytoskeleton during settlement and metamorphosis. The metamorphosis of oysters involves drastic tissue degradation and organ reassembly, so up-regulation of these structural proteins was inevitable.

### Other proteins

In present study, the radial spoke head protein was identified, which is structural components of the axoneme and participate in producing flagellar patterns through regulation of the dynein arms [45]. Cilia-and flagella-associated protein was identified, the radial spoke head protein and cilia-and flagella-associated protein increased and then decreased from PL to MET group, this protein was up-regulated in eMET group (Supplementary Table 6), the result showed that it might be related to velum atrophy.

In this study, ferritin was identified, which increased and then decreased from PL to MET group, this protein was up-regulated in eMET group (Supplementary Table 6). Ferritin participate in neural development and is required multiple times during development [103]. Ferritin is required for embryonic and larval development [104], and also participate in shell formation [105]. The result suggest that ferritin is likely important for *C. angulata* embryo development.

## Conclusions

**Fig. 8:**
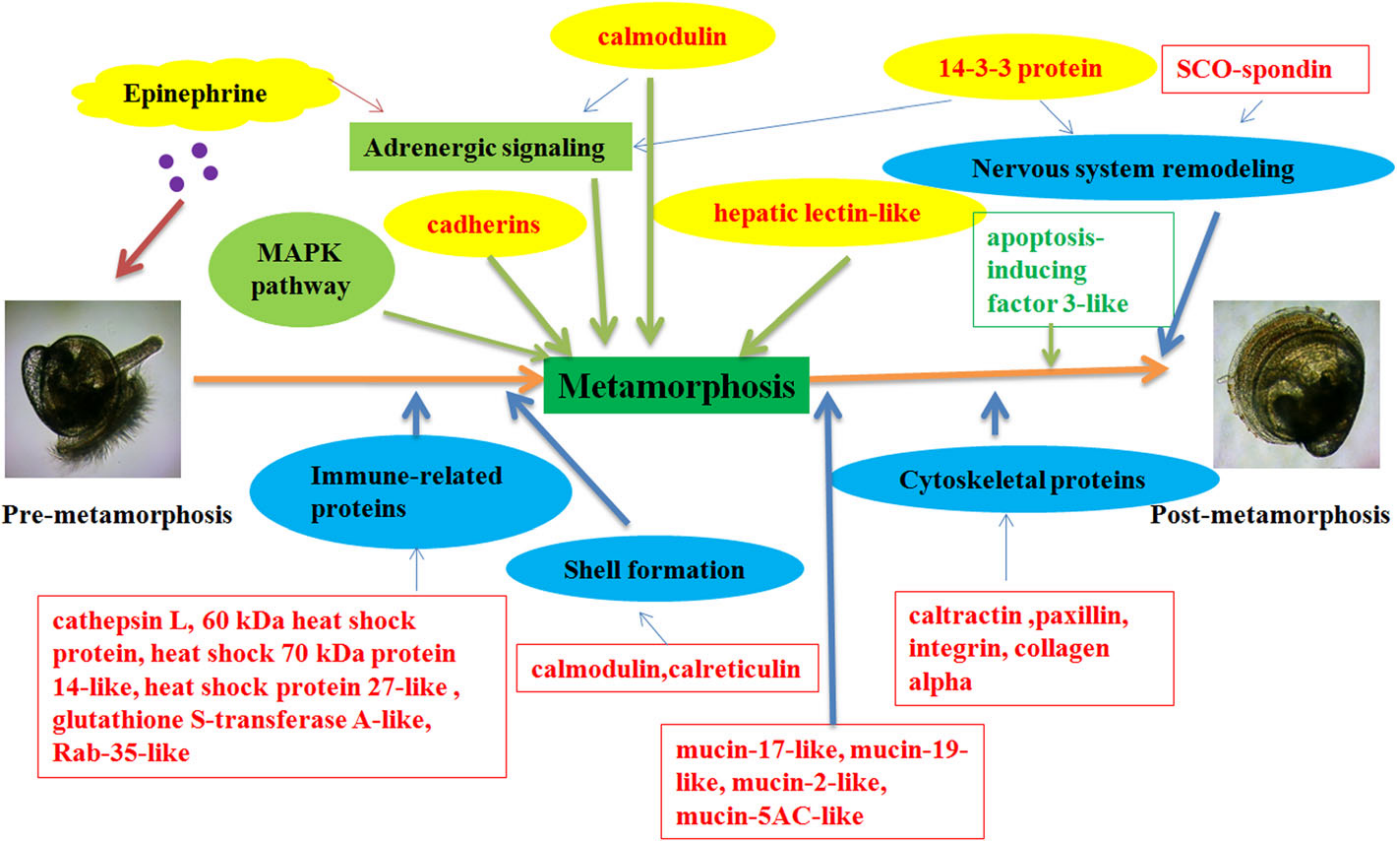
proteins response of oyster *Crassostrea angulata* pediveliger to epinephrine. Red font denote upregulated proteins, green font denote downregulated proteins

In this study, the proteome of larval settlement and metamorphosis induced by epinephrine in the Fujian oyster *C. angulata* was studied by liquid chromatography-mass spectrometry (LC-MS). The DEPs were studied by bioinformatics analysis. The results identified the DEPs involved in the epinephrine signaling pathway, and the levels of these proteins may reflect the developmental stage of oyster larvae. These proteins were up-regulated at the stage of the pediveliger, when epinephrine treatment can induce direct metamorphosis in oysters. The proteins response of oyster *Crassostrea angulata* pediveliger to epinephrine was showed in Fig. 8. In a word, natural settlement and metamorphosis or epinephrine-induced direct metamorphosis involve many biological processes, and how these functions mediate the regulation process needs further research. The focus of future research will be closely related to signal pathways, and the study of protein levels will be more meaningful.

## Methods

### Animal manipulation

The larvae (60–75 μm) were obtained from the Zhangpu oyster aquaculture farm (Fujian Province, China), where the fertilization of *C. angulata* was performed. The larvae were transferred to the laboratory to continue culturing during the pediveliger stage (post eyespot larval stage). The larvae were fed daily with *Isochrysis zhangjiangensis*, *Chlorella vulgaris,* and *Chaetoceros sp*. The seawater temperature was maintained at 24 °C–26 °C, and the water was filtered using 0.22-μm filter membranes. To obtain a consistent growth rate of post-eyespot larvae, 80-mesh screens (pore size: 180 μm) were used to screen immature larvae so that almost all of the individuals were in the same phase of development. After filtration, more than 95% of the larvae could be identified under a microscope as being in the pediveliger stage; 70 to 80% of the larvae used their foot to explore the substratum (Supplementary Fig. 5A). The larvae were retained in sampling boxes. Because the larvae have a preference for settlement substrates, the previous polyacetal film was replaced using a harder resin epoxy insulation board (Supplementary Fig. 5B). The individual density was about 100 ind/mL.

### Sample acquisition

Oyster larvae were cultured in cement ponds. When they were at the stage of the pediveliger, the larvae were collected into 10-L buckets with mesh screens at the point when most of the larvae began to explore substrates, and the larvae were then divided into several sampling boxes. The density of larvae was about 100 ind/mL, and the larvae were at the pediveliger (PL) stage; most of the larvae attached to the bottom of the sampling box after 30 min. Some larvae that did not attach were removed with water from the sampling box, and then the bottom of the sampling box was gently washed with filtered seawater to remove the remaining non-attached larvae. Finally, the settled larvae that had attached to the sampling box were gently brushed down into filtered seawater with a soft brush and collected into freezing tubes; the seawater was removed, and the freezing tube was quickly stored in liquid nitrogen. The sample was labelled as post-attachment (PA). A subsequent PA sample was collected in another sampling box, and then new filtered seawater was added to continue culturing for 10–12 h. The larvae in this sample were collected when secondary shells were observed (Supplementary Fig. 5C). The sample was preserved in liquid nitrogen and labelled as post-metamorphosis sample (post-metamorphosis, MET). For each developmental stage, almost all of the collected individuals were in the same period as confirmed by microscopic examination.

To study the molecular mechanism of direct metamorphosis induced by epinephrine in Fujian oyster larvae without settlement, we also prepared samples of epinephrine-induced larvae (EPI). Coon [32] reported that the best induction effect on the pacific oyster *Crassostrea gigas* occurred when the concentration of epinephrine was between 10^− 4^ and 10^− 5^ M [32]. In this study, combined with our experimental experience, 5 × 10^− 4^ M epinephrine for 2 h was used to induce larvae. After screening, about 70–80% of the pediveligers were observed to explore with their foot; this ensured that most of the larvae could be induced. The larvae were collected and transferred to the sampling box, and epinephrine was added. After about 10 s, almost all the larvae sank to the bottom of the box. After 2 h, most larvae had sunk to the bottom, but some continued to swim. After an additional 0.5 h, the larvae that sank to the bottom were collected as the epinephrine-induced larvae (EPI-sensitive larvae, eSEN) that were at the stage of post-attachment, while the larvae that had been exposed to epinephrine but continued swimming were collected as the epinephrine-insensitive larvae (EPI -insensitive larvae, einSEN). The eSEN larvae were cultured for about 10 h, at which time obvious secondary shells were observed (Supplementary Fig. 5D). The samples were labelled as post-metamorphosis induced by EPI (eMET). Each group of six groups was divided into three independent biological replicates, approximately 4000–5000 per stage, and three technical replicates were performed for each sample to ensure reproducibility.

### Extraction of oyster larvae proteins

For each group sample, protein extraction solution (1 ml, 50 mM Tris 0.1% SDS, 10 mM HEPES pH 8.1) was added to the larvae samples (100 mg), and then 2–3 steel beads were added at 10 min blasts, 30 times/s, boiled at 100 °C for 5 min, and then cooled to room temperature on ice. The samples were centrifuged at 17000×g for 30 min at 4 °C, and the supernatant was collected. Then, samples were immediately suspended in 3–4 times volume of 20% TCA/acetone solution (w/v) with 20 mM DTT, and then placed overnight at − 20 °C. The sample was then centrifuged (17,000×*g*, 15 min, 4 °C), and the supernatant was removed. The sample was washed (0.5 mL, pre-cooled 100% acetone with 20 mM DTT) and placed on ice for five minutes, then centrifuged (17,000×g, 15 min, 4 °C). The sample was washed three times, and the supernatant was removed. The protein pellet was collected, vacuum freeze-dried, and stored at − 70 °C.

### Protein digestion for label-free proteomic analysis

DTT buffer (1 M DTT in 8 M urea buffer per 50 μg protein) was added for a final concentration of 20 mM. Determination of protein concentration used the Bradford method. The sample was kept in a water bath (30 °C) for 30 min, then cooled to room temperature. The protein samples (50 μg) were alkylated with IAA buffer (1 M IAA in 8 M urea buffer) and adjusted to a final concentration of 40 mM IAA, and the mixture was incubated in darkness for 30 min. DTT buffer was added, and the final concentration of DTT was adjusted to 10 mM. The samples then had 7 times the volume of NH_4_HCO_3_ solution added to reduce the concentration of Urea to 1 M. Incubation with trypsin (1 g/50 g protein) (Promega, Madison, WI) was conducted for 12 h at room temperature. The digestion was stopped by adding 5% formic acid (the final concentration was about 0.1%).

### Optimization of the desalination column

One hundred microliters of methanol was added to the desalination column; the sample was centrifuged at 100×g for 1 min, and the supernatant was removed. Then, 100 μL 70% acetonitrile, and 1% formic acid solution were added; the sample was centrifuged at 110×g for 1 min, and the supernatant was removed. Then, 200 μL 1% formic acid solution was added; the sample was centrifuged at 110×g for 1 min, and the supernatant was removed.

### Sample loading and elution

Formic acid was added into the trypsin digestion solution, and the pH value was adjusted to pH 2–pH 3. The trypsin digestion solution was added into the desalination column; the sample was centrifuged at 110×g for 1 min, and the supernatant was removed. Then, 200 μl 1% formic acid solution was added; the sample was centrifuged at 110×g for 1 min, and supernatant was removed. The sample was transfered from the desalination column to a new centrifuge tube, and then 100 μl 50, 75, and 100% acetonitrile (containing 1% formic acid) were added to elute the peptide fragments from the column, and the solution was collected. The solution was then freeze-dried to powder.

### Sample preparation and mass spectrometry analysis

Freeze-dried peptide segments were dissolved in 30–50 μl of a solution containing 0.1% formic acid and 2% acetonitrile. Then, the peptide segments sample was centrifuged at 12000×g for 10 min. The supernatant was filtered with a 0.22-μm microporous membrane, and the polypeptide solution was mixed in the sample bottle using an oscillator. A Thermo Scientific-Q Exactive™, a high-performance benchtop Quadrupole-Orbitrap™ Mass Spectrometer and a WATERS nano UPLC liquid phase system were employed. The Flex source was used for the ion source. The mobile phase was acetonitrile solution; phase A was 2% acetonitrile with 0.1% formic acid solution, and phase B was 98% acetonitrile with 0.1% formic acid solution.

### Data analysis

Data analysis was performed with three replicate injections of each sample using a Q-Exactive mass spectrometer with the following parameters: duration: 120 min; detection method: positive ion detection; parent ion scanning scope: 300–1800 m/z. Each sample was analyzed three times. The program consisted of a 70,000 resolution full-scan MS scan, and the 10 most abundant peaks were selected for MS/MS using a 17,500 resolution scan. The ion selection window was a 1.6 mass-to-charge (m/z) ratio, and the normalized collision energy was 30 eV. The program used a 40s dynamic exclusion window to avoid repeated selection of peptides for MS/MS. MS1 resolution at M/Z 200: 70,000; MS2 resolution at M/Z 200: 17,500. The mass-to-charge ratios of polypeptide fragments were obtained by collecting 20 fragment patterns (MS2 scan, HCD) after each full scan.

Raw files were processed to peak lists. The original data were analyzed with three replicate injections of each sample using Proteome Discoverer software version 1.4, a protein identification software of ThermoFisher Scientific, and analyzed using SIEVE software, to quantify all of the detected peaks. The *Crassostrea* protein database was downloaded from NCBI (*Crassostrea gigas* has been sequenced with a genome-wide database), and then combined with the protein database predicted by the *Crassostrea* transcriptome CDS. The software Scaffold version 4.4 was selected to carry out the relative quantification of the proteins. The database search parameters were as follows: main search ppm: 6; MS/MS tolerance ppm: 20; missed cleavage: 2; De-Isotopic: ture; enzyme: trypsin; fixed modification: carbamidomethyl (C); variable modification: oxidation (M), acetyl (protein N-term); LFQ: ture; LFQ min ratio count: 1; decoy database pattern: reserve; match between runs: 2 min; protein or peptide FDR: 0.01.

### GO and KEGG annotation and enrichment

The annotations included GO and KEGG protein annotations through transcripts and the NCBI protein database based on the GI number. Fisher’s Exact test was used to evaluate the significance of enrichment levels of proteins under each GO term. The protein sequences were annotated with the KEGG database. The significance of the enrichment level of each pathway was calculated by Fisher’s Exact test to determine the significance level.

### Experimental validation using RT-qPCR

The mRNA expression profiles of six differentially expressed genes were validated using SYBR Green fluorescent RT-qPCR with the Fujian oyster reference gene encoding elongation factor 1(EF1-F) (c233532_g1; gi|524,916,452|ref.|XP_005113003.1| PREDICTED:elongation factor 1-alpha [*Aplysia californica]*) and Ubiquitin (UBQ) for each sample. This SYBR Green qPCR assay was conducted using a QuantStudio 6 Flex Real-Time PCR System following the manufacturer’s instructions, and relative gene expression levels were analyzed using the 2^−ΔΔCT^ method. This experiment was conducted in triplicate. Primer nucleotide sequences were designed using Primer Premier 6 software (Premier Biosoft, USA) (Supplementary Table 14). PCR amplification experiments were performed under the following conditions: 95 °C for 30 s, then 40 cycles of 95 °C for 5 s, 55 °C for 30 s, and 72 °C for 30 s. Three replicates were performed for each developmental stage to ensure reproducibility.

## Supplementary information

**Additional file 1: Supplementary Figure 1.** The basic information of proteome identification. **Supplementary Figure 2.** The overall distribution of quantitative proteomics. A. Protein mass distribution; B. Peptide length distribution; C. Distribution of peptides’s sequence coverage; D. Distribution of unique peptide. **Supplementary Figure 3.** Gene ontology classification of oyster proteome. **Supplementary Figure 4.** KEGG function classification of oyster proteome. A-cellular processes, B-environmental information processing, C-genetic information processing, D-metabolism, E-organismal system. **Supplementary Figure 5.** Embryonic development of Fujian oyster *Crassostrea angulate* (A) Post eyespot larvae; (B) the collection box in Fujian oyster *C. angulate;* (C) the larvae after metamorphosis; (D) the larvae after metamorphosis induced by epinephrine.

**Additional file 2: Supplementary Table 1.** Compared with PL, High and low level expression for differentially abundant proteins in PA

**Additional file 3: Supplementary Table 2.** Compared with PA, High and low level expression for differentially abundant proteins in MET

**Additional file 4: Supplementary Table 3.** Compared with PA, High and low level expression for differentially abundant proteins in e-SEN

**Additional file 5: Supplementary Table 4.** Compared with MET, High and low level expression for differentially abundant proteins in eMET

**Additional file 6: Supplementary Table 5.** The differentially expressed proteins among six groups with protein volumes calculated

**Additional file 7: Supplementary Table 6.** The differentially expressed proteins with the same name were merged among six groups. **Supplementary Table 7.** Extremely significant enrichment GO term both in PL-PA and PA-MET groups. **Supplementary Table 8.** Extremely significant enrichment GO term in PL-eSEN -einSEN groups. **Supplementary Table 9.** Extremely significant enrichment GO term in PL-MET -eMET groups. **Supplementary Table 10.** Extremely significant enrichment KEGG pathway of PL-MET group. **Supplementary Table 11.** Extremely significant enrichment pathway only in eSEN-eMET group against PL-eSEN group. **Supplementary Table 12.** Extremely significant enrichment pathway only in PL-einSEN group against PL-eSEN group. **Supplementary Table 13.** Protein abbreviations and corresponding full name

**Additional file 8: Supplementary Table 14.** Primer of qPCR

## Data Availability

All data generated or analysed during this study are included in this published article and its Additional files.

## Abbreviations

GO: Gene ontology
KEGG: Kyoto Encyclopedia of Genes and Genomes
MS: Mass spectrometry
EPI: Epinephrine
PL: Pediveliger (post eyespot larvae, pre-settlement)
PA: Post-attachment
MET: Post-metamorphosis
eSEN: EPI-sensitive larvae
einSEN: EPI -insensitive larvae
eMET: Post-metamorphosis induced by EPI
RT-qPCR: RealTime Quantitative PCR
LC-MS: Liquid chromatography-mass spectrometry
SDS: Superoxide dismutase
HEPES: Hydroxyethyl piperazineethanesulfonic acid
TCA: Trichloroacetic acid
DTT: Dithiothreitol
IAA: Iodoacetamide
NCBI: The US National Center for Biotechnology Information
CDS: Coding sequences
kDa: Kilodaltons
DEPs: Differentially expressed proteins

## References

[1] FAO. FAO Fisheries Department, Fishery Information, Data and Statistics Unit, FishStat Plus: Universal software for fishery statistical time series, Version 2.3. 2000.

[2] Qin J, Huang ZX, Chen J, Zou Q, You WW, Ke CH (2012). Sequencing and de novo analysis of *Crassostrea angulata* (Fujian oyster) from 8 different developing phases using 454 GSFLx. PLoS One.

[3] Ministry of Agriculture. Yearbook of Fisheries Statistics in China. 2017.

[4] Balseiro P, Moreira R, Chamorro R, Figueras A, Novoa B (2013). Immune responses during the larval stages of *Mytilus galloprovincialis*: metamorphosis alters immunocompetence, body shape and behavior. Fish Shellfish Immun.

[5] USEPA. Methods for measuring the acute toxicity of effluents and receiving waters to freshwater and marine organisms, 5th Edition, EPA-821-R-02-012 ,2002.

[6] Benton TG, Plaistow SJ, Coulson TN (2006). Complex population dynamics and complex causation: devils, details and demography. P Roy Soc B-Biol Sci.

[7] Beldade P, Mateus ARA, Keller RA (2011). Evolution and molecular mechanisms of adaptive developmental plasticity. Mol Ecol.

[8] West-Eberhard MJ (2003). Developmental plasticity and evolution.

[9] Hadfield MG (2000). Why and how marine-invertebrate larvae metamorphose so fast. Semin Cell Dev Biol.

[10] Degnan SM, Degnan BM (2010). The initiation of metamorphosis as an ancient polyphenic trait and its role in metazoan life-cycle evolution. Phil Trans R Soc B.

[11] Underwood A, Keough MJ (2001). Supply-side ecology: the nature and consequences of variations in recruitment of intertidal organisms. Unknown..

[12] Yang B, Li L, Pu F, You WW, Huang HQ, Ke CH (2015). Molecular cloning of two molluscan caspases and gene functional analysis during *Crassostrea angulata* (Fujian oyster) larval metamorphosis. Mol Biol Rep.

[13] Yang BY, Li Y, Chen ZW. Funtional analysis on a novel gene SMRP1 from Fujian *Crassostrea angulata*. Food Res Dev. 2019;10.

[14] Cranfield HJ (1973). Observations on the behaviour of the pediveliger of *Ostrea edulis* during attachment and cementing. Mar Biol.

[15] Cranfield HJ (1973). Observations on the function of the glands of the foot of the pediveliger of *Ostrea edulis* during settlement. Mar Biol.

[16] Cranfield HJ (1974). Observations on the morphology of the mantle folds of the pediveliger of *Ostrea Edulis* and their function during settlement. J Mar Biol Assoc UK.

[17] Ke CH, Feng DQ (2006). Researches on Larval Settlement and Metamorphosis of Marine Benthos. J Xiamen Univ (Natural Science).

[18] Bishop CD, Huggett MJ, Heyland A, Hodin J, Brandhorst BP (2006). Interspecific variation in metamorphic competence in marine invertebrates: the significance for comparative investigations into the timing of metamorphosis. Integr Comp Biol.

[19] Yang C, Su XR, Li TW (2003). The cultural technique of Pacific oyster, *Crassostrea gigas*. Fisheries Sci.

[20] Coon SL, Bonar DB, Weiner RM (1986). Chemical production of cultchless oyster spat using epinephrine and norepinephrine. Aquaculture..

[21] Bonar BD, Coon SL, Walch M, Weiner MR, Fitt W (1990). Control of oyster settlement and metamorphosis by endogenous and exogenous chemical cues. Bull Mar Sci.

[22] Coon SL, Bonar DB, Weiner RM (1985). Induction of settlement and metamorphosis of the Pacific oyster*, Crassostrea gigas* (Thunberg), by L-DOPA and catecholamines. J Exp Mar Biol Ecol.

[23] Shpigel M, Coon SL, Kleinot P (1989). Growth and survival of cultchless spat of *Ostrea edulis* Linnaeus 1750 produced using epinephrine and shell chips. J Shellfish Res.

[24] Dupuy JL (1972). Ftivkin S. the development of laboratory techniques for the production of cultch-free spat of the oyster, *Crassostrea virginica*. Chesap Sci.

[25] Helm MM, Bourne N. Hatchery culture of bivalves, a practical manual. In: Lovatelli, A. (Ed.), FAO Fisheries Technical Paper No. 471, Rome, 2004; (177 pp).

[26] Lucas JS, Southgate PC. Bivalve molluscs. In: Lucas, JS, Southgate PC (Eds.) Aquaculture (Second edition): Farming Aquatic Animals and Plants. Wiley-Blackwell, Ltd, 2012, pp. 541–66.

[27] García-Lavandeira M, Silva A, Abad M, Pazos JA, Sánchez LJ, Pérez-Parallé L (2005). Effects of GABA and epinephrine on the settlement and metamorphosis of the larvae of four species of bivalve molluscs. J Exp Mar Biol Ecol.

[28] Teh CP, Zulfigar Y, Tan SH. Epinephrine and L-DOPA promote larval settlement and metamorphosis of the tropical oyster, *Crassostrea iredalei* (Faustino,1932): an oyster hatchery perspective. Aquaculture. 2012:338–41 260–263.

[29] Pérez-Bustamante IS, García-Esquivel Z (2017). Effect of five chemical compounds on larval metamorphosis of the Cortez geoduck clam, Panopea globosa. Aquaculture.

[30] Susanne V, Penny ME, Xiaoxu L, Wikfors GH, Alyssa J (2018). First report of a putative involvement of the NMDA pathway in pacific oyster (*Crassostrea gigas*) development: effect of NMDA receptor ligands on oyster metamorphosis with implications for bivalve hatchery management. Aquaculture..

[31] Joyce A, Vogeler S (2018). Molluscan bivalve settlement and metamorphosis: neuroendocrine inducers and morphogenetic responses. Aquaculture..

[32] Coon SL, Bonar DB (1987). Pharmacological evidence that alpha1-adrenoceptors mediate metamorphosis of the pacific oyster, *Crassostrea gigas*. Neuroscience.

[33] Yang B, Qin J, Shi B, Han G, Chen J, Huang H, Ke C (2012). Molecular characterization and functional analysis of adrenergic like receptor during larvae metamorphosis in *Crassostrea angulata*. Aquaculture..

[34] Fiedler TJ, Hudder A, Mckay SJ, Shivkumar S, Capo TR, Schmale MC, Walsh PJ (2010). The transcriptome of the early life history stages of the California Sea hare *Aplysia californica*. Comp Biochem Physiol Part D Genomics Proteomics.

[35] Huan P, Wang H, Dong B, Liu B (2012). Identification of differentially expressed proteins involved in the early larval development of the Pacific oyster *Crassostrea gigas*. J Proteome.

[36] Lopez JL, Abalde SL, Fuentes J (2005). Proteomic approach to probe for larvae proteins of the mussel *Mytilus galloprovincialis*. Mar Biotechnol (NY).

[37] Thiyagarajan V, Qian PY (2008). Proteomic analysis of larvae during development, attachment, and metamorphosis in the fouling barnacle, *Balanus amphitrite*. Proteomics.

[38] Thiyagarajan V, Wong T, Qian PY (2009). 2D gel-based proteome and phospho proteome analysis during larvae metamorphosis in two major marine biofouling invertebrates. J Proteome Res.

[39] Wang H, Zhang H, Wong YH, Voolstra C, Ravasi TB, Bajic V, Qian PY (2010). Rapid transcriptome and proteome profiling of a non-model marine invertebrate, *Bugula neritina*. Proteomics.

[40] Zhang Y, Sun J, Xiao K, Arellano SM, Thiyagarajan V, Qian PY (2010). 2D gel-based multiplexed proteomic analysis during larvae development and metamorphosis of the biofouling polychaete tubeworm *Hydroides elegans*. J Proteome Res.

[41] Chandramouli KH, Mok FS, Wang H, Qian PY (2011). Phosphoproteome analysis during larvae development and metamorphosis in the spionid polychaete *Pseudopolydora vexillosa*. BMC Dev Biol.

[42] Chen ZF, Zhang H, Wang H, Matsumura K, Wong Y, Ravasi T, Qian PY (2014). Quantitative proteomics study of larvae settlement in the barnacle *Balanus amphitrite*. PLoS One.

[43] Sun J, Zhang Y, Thiyagarajan V, Qian P, Qiu JW (2010). Protein expression during the embryonic development of a gastropod. Proteomics..

[44] Huan W, Xu F, Qu T, Zhang R, Li L, Que H, Zhang G (2015). Identification of thyroid hormones and functional characterization of thyroid hormone receptor in the Pacific oyster, *Crassostrea gigas*, provide insight into evolution of the thyroid hormone system. PLoS One.

[45] Mendoza-Porras O, Botwright NA, McWilliam SM, Cook MT, Harris JO, Wijffels G, Colgrave ML (2014). Exploiting genomic data to identify proteins involved in abalone reproduction. J Proteome.

[46] Di G, Kong X, Miao X, Zhang Y, Huang M, Gu Y, You W, Zhang J, Ke C (2017). Proteomic analysis of trochophore and veliger larvae development in the small abalone *Haliotis diversicolor*. BMC Genomics.

[47] Miao Y, Zhang L, Sun Y, Jiao W, Li Y, Sun J, Wang Y, Wang S, Bao Z, Liu W (2015). Integration of transcriptomic and proteomic approaches provides a core set of genes for understanding of scallop attachment. Mar Biotechnol.

[48] Shen M, Di G, Li M, Fu J, Dai Q, Miao X, Huang M, You W, Ke C (2018). Proteomics studies on the three larval stages of development and metamorphosis of *Babylonia areolata*. Sci Rep-Uk.

[49] Wang H, Qian PY (2010). Involvement of a novel p38 mitogen-activated protein kinase in larvae metamorphosis of the polychaete *Hydroides elegans* (Haswell). J Exp Zool B Mol Dev Evol.

[50] Chandramouli KH, Sun J, Mok FS, Liu L, Qiu JW, Ravasi T, Qian PY (2013). Transcriptome and quantitative proteome analysis reveals molecular processes associated with larvae metamorphosis in the polychaete *Pseudopolydora vexillosa*. J Proteome Res.

[51] Zhu W, Smith JW, Huang CM (2010). Mass spectrometry-based label-free quantitative proteomics. J Biomed Biotechnol.

[52] Searle BC (2010). Scaffold: a bioinformatic tool for validating MS/MS-based proteomic studies. Proteomics..

[53] Li HJ, Li Q, Yu H, Du SJ (2019). Developmental dynamics of myogenesis in Pacific oyster Crassostrea gigas. Comp Biochem Phys B.

[54] Heyland A, Moroz LL (2006). Signaling mechanisms underlying metamorphic transitions in animals. Integr Comp Biol.

[55] Zheng Z, Hao R, Xiong X, Jiao Y, Deng Y, Du X (2019). Developmental characteristics of pearl oyster *Pinctada fucata martensii* : insight into key molecular events related to shell formation, settlement and metamorphosis. BMC Genomics.

[56] Denison M (1995). Xenobiotic-inducible transcription of cytochrome P450 genes. J Biol Chem.

[57] Raijmakers MTM, Bruggeman SWM, Steegers EA, Peters WH (2002). Distribution of components of the glutathione detoxification system across the human placenta after uncomplicated vaginal deliveries. Placenta..

[58] Heras H, Garin CF, Pollero RJ (1998). Biochemical composition and energy sources during embryo development and in early juveniles of the snail *Pomacea canaliculata* (Mollusca: Gastropoda). J Exp Zool.

[59] Nomura M, Nakajima A, Inaba K (2009). Proteomic profiles of embryonic development in the ascidian *Ciona intestinalis*. Develop Biol.

[60] Jensen RA, Morse DE, Petty RL, Hooker N (1990). Artificial induction of larval metamorphosis by free fatty acids. Mar Ecol-Prog Ser.

[61] Clare AS (1996). Signal transduction in barnacle settlement: calcium re-visited. Biofouling..

[62] Yamamoto T, OgawaY IN (1998). Effect of the poloidal field coil system on the ramp-up scenario with L/H transition. Fusion Eng Des.

[63] Zhang F, Chen HW, Pei YQ (2012). Expression of calmodulin and myosin light chain kinase during larvae settlement of the barnacle *Balanus amphitrite*. PLoS One.

[64] Li S, Xu Y, Matsumura K, Zhang Y, Zhang G, Qi SH, Qian PY (2012). Evidence for the involvement of p38 MAPK activation in barnacle larvae settlement. PLoS One.

[65] Zhang G, He LS, Him Wong Y, Xu Y, Zhang Y, Qian PY (2015). p38 MAPK regulates PKAα and CUB-serine protease in *Amphibalanus amphitrite* cyprids. Sci Rep.

[66] Thamm K, Seaver C (2008). Notch signaling during larvae and juvenile development in the polychaete annelid *Capitella sp I*. Dev Biol.

[67] Zhang G, He LS, Wong YH, Qian PY (2013). MKK3 was involved in larvae settlement of the barnacle *Amphibalanus amphitrite* through activating the kinase activity of p38MAPK. PLoS One.

[68] Balseiro SR, Besbes O, Weintraub GY. Auctions for online display advertising exchanges:approximations and design. Fourteenth Acm Conference on Electronic Commerce. ACM. 2013:53–4.

[69] Davidson B, Swalla BJ (2002). A molecular analysis of ascidian metamorphosis reveals activation of an innate immune response. Development..

[70] Sharon N (1984). Surface carbohydrates and surface lectins are recognition determinants in phagocytosis. Immunol Today.

[71] Gunter HM, Degnan BM (2007). Developmental expression of Hsp90, Hsp70 and HSF during morphogenesis in the vetigastropod *Haliotis asinina*. Dev Genes Evol.

[72] Kim KY, Lee SY, Cho YS, Bang IC, Kim KH, Kim DS, Nam YK (2007). Molecular characterization and mRNA expression during metal exposure and thermal stress of copper/zinc- and manganese-superoxide dismutases in disk abalone, *Haliotis discus discus*. Fish Shellfish Immunol.

[73] Woods RG, Roper E, Gauthier M, Bebell LM, Sung K, Degnan BM, Lavin MF (2004). Gene expression during early ascidian metamorphosis requires signalling by hemps, an EGF-like protein. Development..

[74] Roberts B, Davidson B, MacMaster G, Lockhart V, Ma E, Wallace SS, Swalla BJ (2007). A complement response may activate metamorphosis in the ascidian *Boltenia villosa*. Dev Genes Evol.

[75] Grasso LC, Maindonald J, Rudd S, Hayward DC, Saint R, Miller DJ, Ball EE (2008). Microarray analysis identifies candidate genes for key roles in coral development. BMC Genomics.

[76] Maki JS, Mitchell R (1985). Involvement of lectins in the settlement and metamorphosis of marine invertebrate larvae. B Mar Sci.

[77] Matsutani T, Morishita K, Seki T, Mori K (2001). Involvement of lectin-like factors in larval settlement and metamorphosis in the abalone, *Haliotis discus hannai*. Tohoku J Agricult Res.

[78] Bao XB, He CB, Fu CD, Wang B, Liu WD (2015). A C-type lectin fold gene from Japanese scallop *Mizuhopecten yessoensis*, involved with immunity and metamorphosis. Genet Mol Res.

[79] Takeichi M (1988). The cadherins: cell-cell adhesion molecules controlling animal morphogenesis. Development..

[80] Chen ZF, Wang H, Qian PY (2012). Characterization and expression of calmodulin gene during larvae settlement and metamorphosis of the polychaete *Hydroides elegans*. Comp Biochem Physiol B Biochem Mol Biol.

[81] Chambon JP, Soule J, Pomies P, Fort P, Sahuquet A, Alexandre D, Mangeat PH, Baghdiguian S (2002). Tail regression in *Ciona intestinalis* (Prochordate) involves a Caspase-dependent apoptosis event associated with ERK activation. Development.

[82] Haley BA, Hales B, Brunner EL, Kovalchik K, Waldbusser GG (2018). Mechanisms to explain the elemental composition of the initial aragonite Shell of larval oysters. Geochem Geophy Geosy.

[83] Jackson DJ, Wö Rheide W, Degnan BM (2007). Dynamic expression of ancient and novel molluscan shell genes during ecological transitions. BMC Evol Biol.

[84] Pernille R (2008). Quality control in an unreliable world. EMBO J.

[85] Feng D, Li Q, Yu H, Kong L, Du S (2017). Identification of conserved proteins from diverse shell matrix proteome in *Crassostrea gigas*: characterization of genetic bases regulating shell formation. Sci Rep.

[86] Du X, Fan G, Jiao Y, Zhang H, Guo X, Huang R (2017). The pearl oyster *Pinctada fucata martensii* genome and multi-omic analyses provide insights into biomineralization. Gigascience..

[87] Levi-Kalisman Y, Falini G, Addadi L, Weiner S (2001). Structure of the nacreous organic matrix of a bivalve mollusk shell examined in the hydrated state using Cryo-TEM. J Struct Biol.

[88] Chung HJ, Sehnke PC, Ferl RJ (1999). The 14-3-3 proteins: cellular regulators of plant metabolism. Trends Plant Sci.

[89] Yumang F (2004). Analysis of nonlinear effects on wdm system performance and a novel wdm channel monitoring method through hos-based blind signal separation. Org Lett.

[90] Oksvold MP, Huitfeldt HS, Langdon WY (2004). Identification of 14-3-3 zeta as an EGF receptor interacting protein. FEBS Lett.

[91] Shandala T, Woodcock JM, Ng Y, Biggs L, Skoulakis EM, Brooks DA, Lopez AF (2011). Drosophila 14-3-3ε has a crucial role in anti-microbial peptide secretion and innate immunity. J Cell Sci.

[92] Skoulakis EMC, Davis RL (1998). 14-3-3 proteins in neuronal development and function. Mol Neurobiol.

[93] Toyo-oka K, Wachi T, Hunt RF, Baraban SC, Taya S, Ramshaw H, Kaibuchi K, Schwarz QP, Lopez AF, Wynshaw-Boris A (2014). 14-3-3ε and ζ regulate neurogenesis and differentiation of neuronal progenitor cells in the developing brain. J Neurosci.

[94] Bunney TD, De Boer H, Levin M (2003). Fusicoccin signaling reveals 14–3-3 protein function as a novel step in left-right patterning during amphibian embryogenesis. Development..

[95] Vera A, Stanic K, Montecinos H, Torrejón M, Marcellini S, Caprile T (2013). Sco-spondin from embryonic cerebrospinal fluid is required for neurogenesis during early brain development. Aquac Res.

[96] Yamauchi T, Fujisawa H (1981). Tyrosine 3-monooxygenase is phosphorylated by Ca2+, calmodulin-dependent protein kinase, followed by activation by activator protein. Biochem Biophys Res Commun.

[97] Ichimura T, Isobe T, Okuyama T, Yamauchi T, Fujisawa HB (1987). 14-3-3 protein is an activator protein that activates tryptophan 5-monooxygenase and tyrosine 3-monooxygenase in the presence of Ca2+, calmodulin-dependent protein kinase II. FEBS Lett.

[98] Nagatsu T, Levitt M, Udenfriend S (1964). Tyrosine hydroxylase. The initial step in norepinephrine biosynthesis. J Biol Chem.

[99] Jéquier E, Lovenberg W, Sjoerdsma A (1967). Tryptophan hydroxylase inhibition: the mechanism by which p-chlorophenylalanine depletes rat brain serotonin. Mol Pharmacol.

[100] Sullivan KMC, Scott K, Zuker CS, Rubin GM (2000). The ryanodine receptor is essential for larval development in drosophila melanogaster. P Natl Acad Sci USA.

[101] Shi YB, Fu L, Hasebe T, Ishizuya-Oka A (2007). Regulation of extracellular matrix remodeling and cell fate determination by matrix metalloproteinase stromelysin-3 during thyroid hormone-dependent post-embryonic development. Pharmacol Ther.

[102] Royer V, Hourdry A, Fraichard S, Bouhin H (2004). Characterization of a putative extracellular matrix protein from the beetle Tenebrio molitor: hormonal regulation during metamorphosis. Dev Genes Evol.

[103] Tennessen JM, Bertagnolli NM, Evans J, Sieber MH, Cox J, Thummel CS (2014). Coordinated metabolic transitions during drosophila embryogenesis and the onset of aerobic glycolysis. G3 (Bethesda Md).

[104] Tang X, Zhou B (2013). Ferritin is the key to dietary iron absorption and tissue iron detoxification in Drosophila melanogaster. FASEB J.

[105] Zhang Y, Meng QX, Jiang TM, Wang HZ, Xie LP, Zhang RQA (2003). Novel ferritin subunit involved in shell formation from the pearl oyster (*Pinctada fucata*). Comp Biochem Physiol B-Biochem Mol Biol.

