# Supplementary material for: Proteome of larval metamorphosis induced by epinephrine in the Fujian oyster Crassostrea angulata
Source: BMC Genomics. 2020 Sep 29;21:675. doi: 10.1186/s12864-020-07066-z (PMC7525975; doi:10.1186/s12864-020-07066-z)

**Titles and legends of supplementary figures**

**Supplementary Fig. 1** The basic information of proteome identification

**Supplementary Fig. 2** The overall distribution of quantitative proteomics

A．Protein mass distribution；B. Peptide length distribution；C. Distribution of peptides’s sequence coverage；D. Distribution of unique peptide

**Supplementary Fig.3** Gene ontology classification of oyster proteome

**Supplementary Fig.4** KEGG function classification of oyster proteome

A-cellular processes, B-environmental information processing, C-genetic information processing, D-metabolism, E-organismal system

**Supplementary Fig.5** Embryonic development of Fujian oyster *Crassostrea angulate* **(A)**Post eyespot larvae; **(B)** the collection box in Fujian oyster *C. angulate;* **(C)**the larvae after metamorphosis; **(D)** the larvae after metamorphosis induced by epinephrine.

**Supplementary Fig.1**


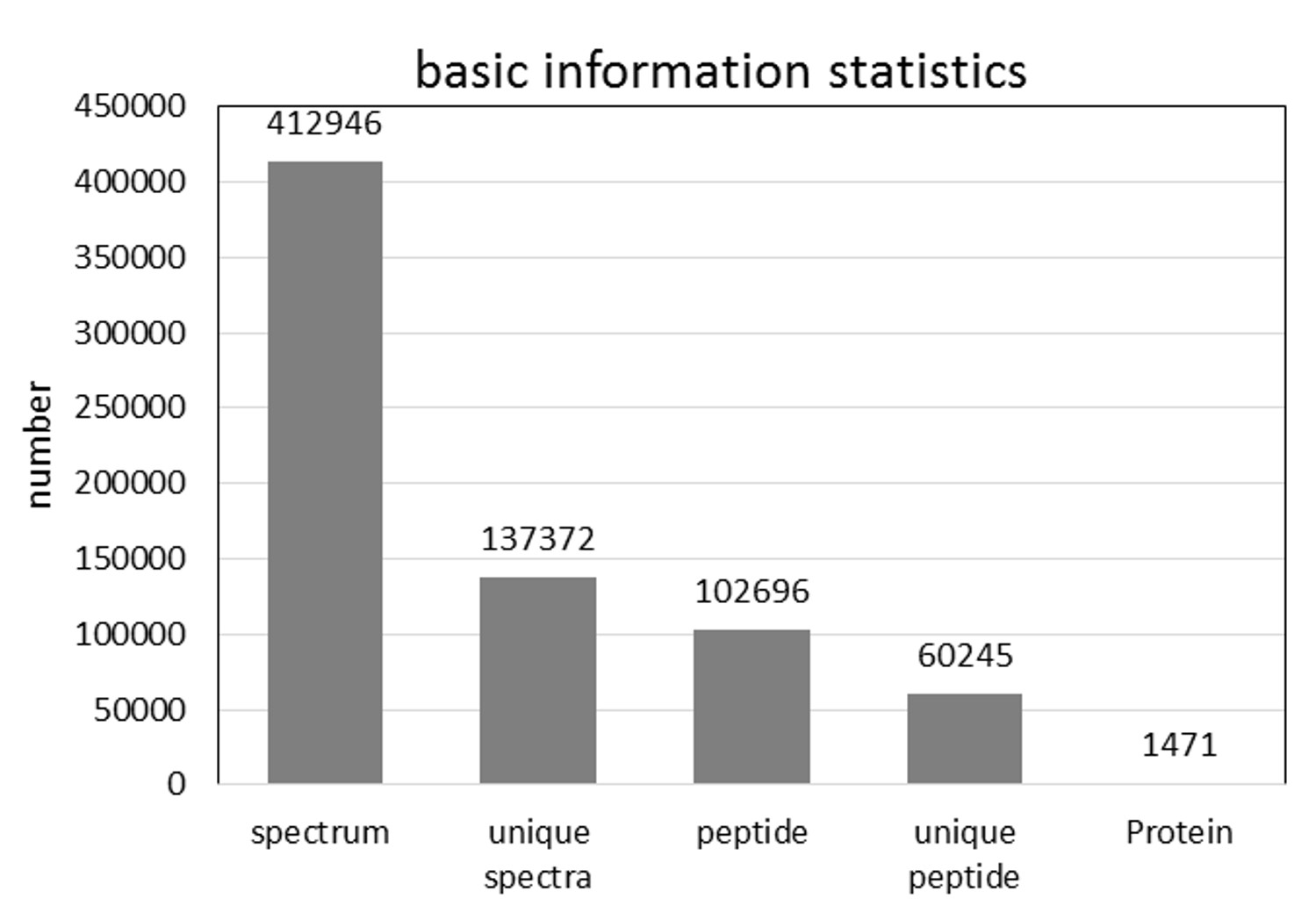


**Supplementary Fig.2**


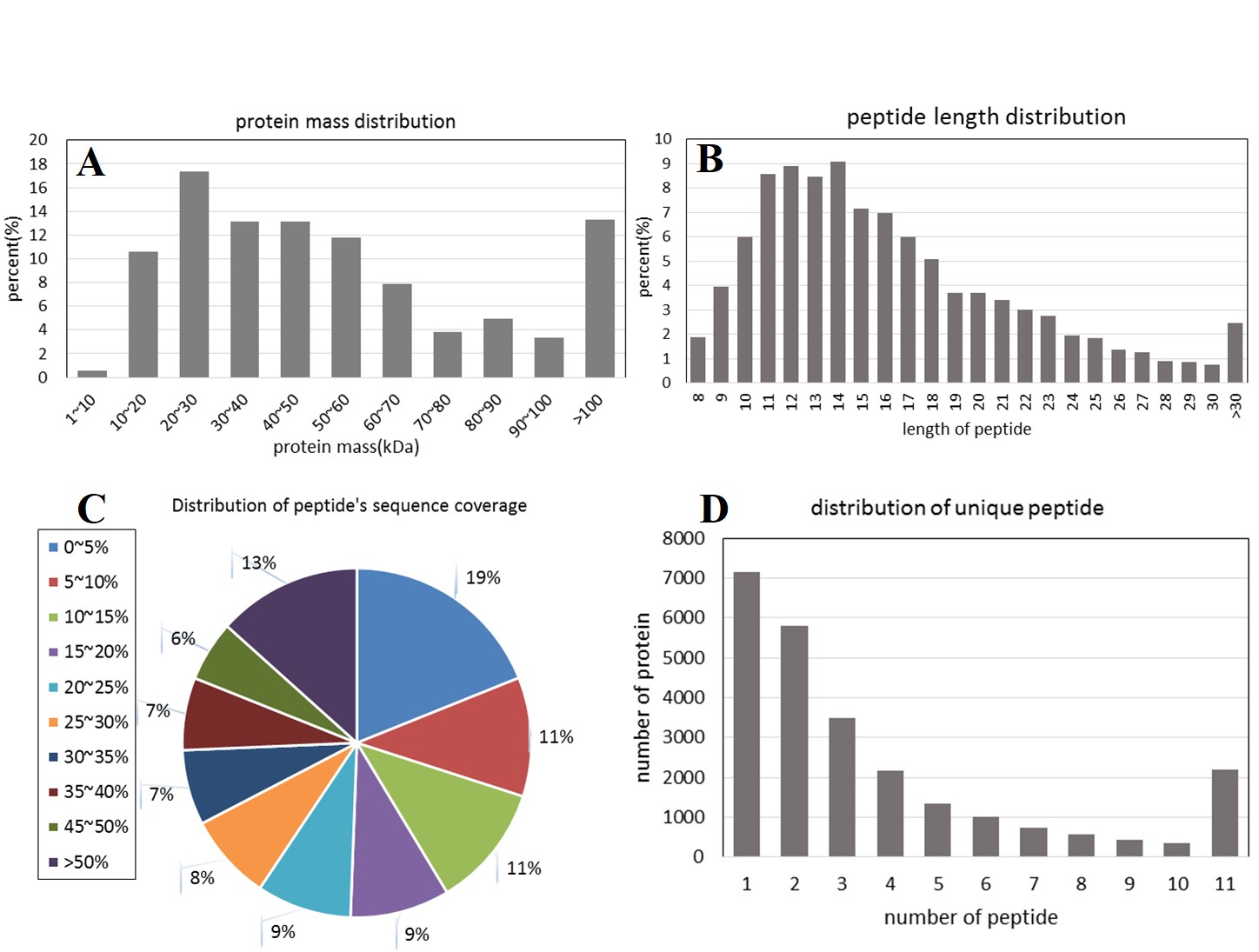


**Supplementary Fig.3**


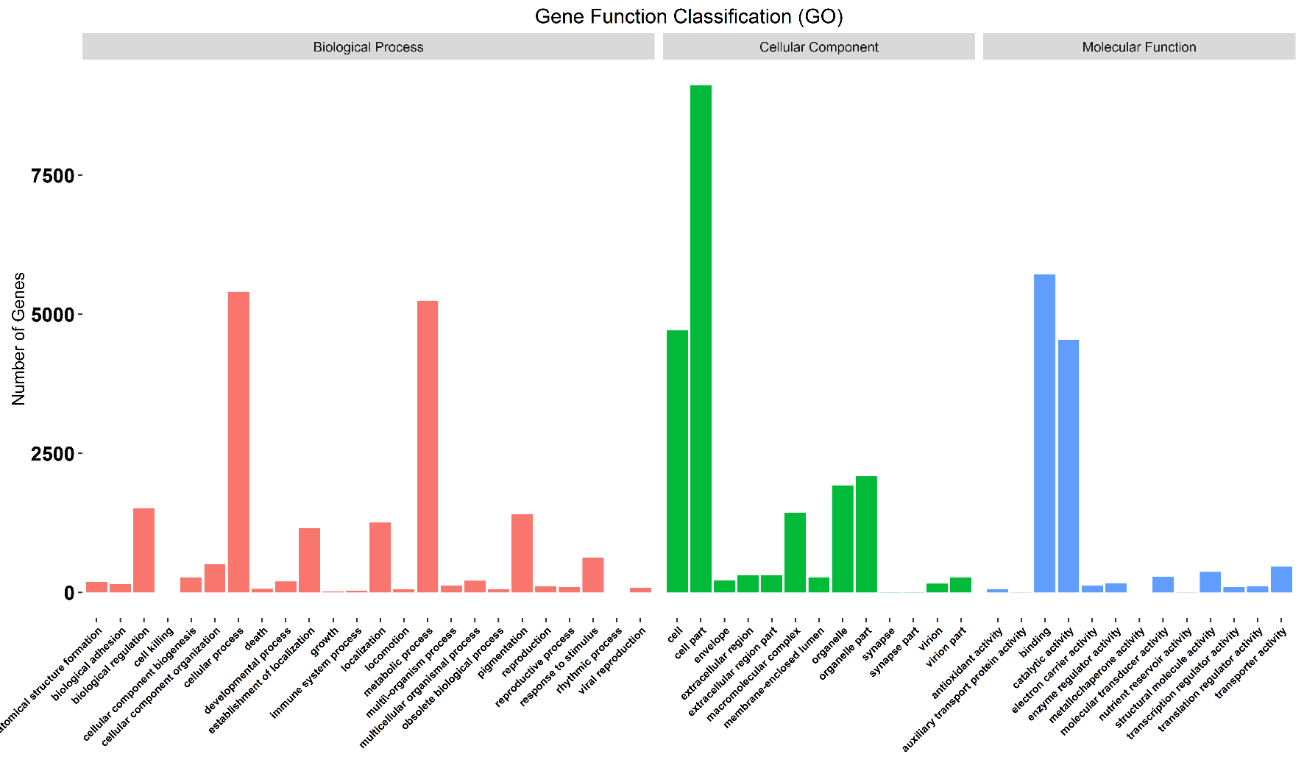


**Supplementary Fig.4**

**
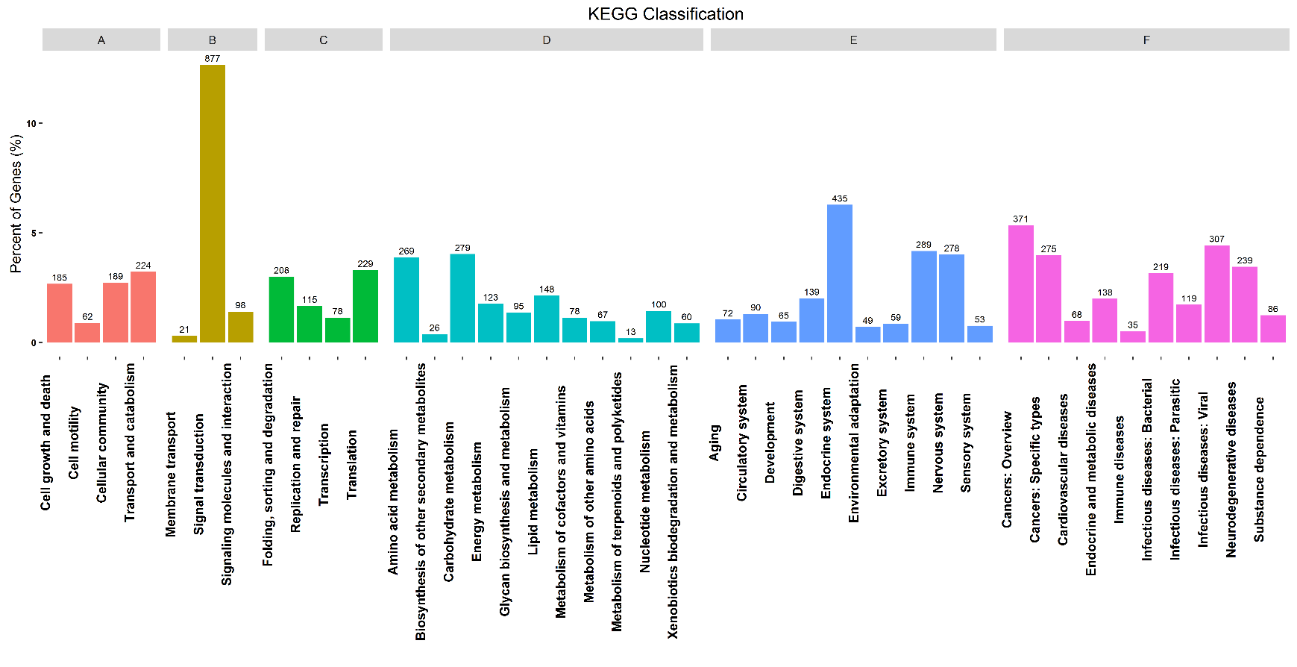
**

**Supplementary Fig.5**


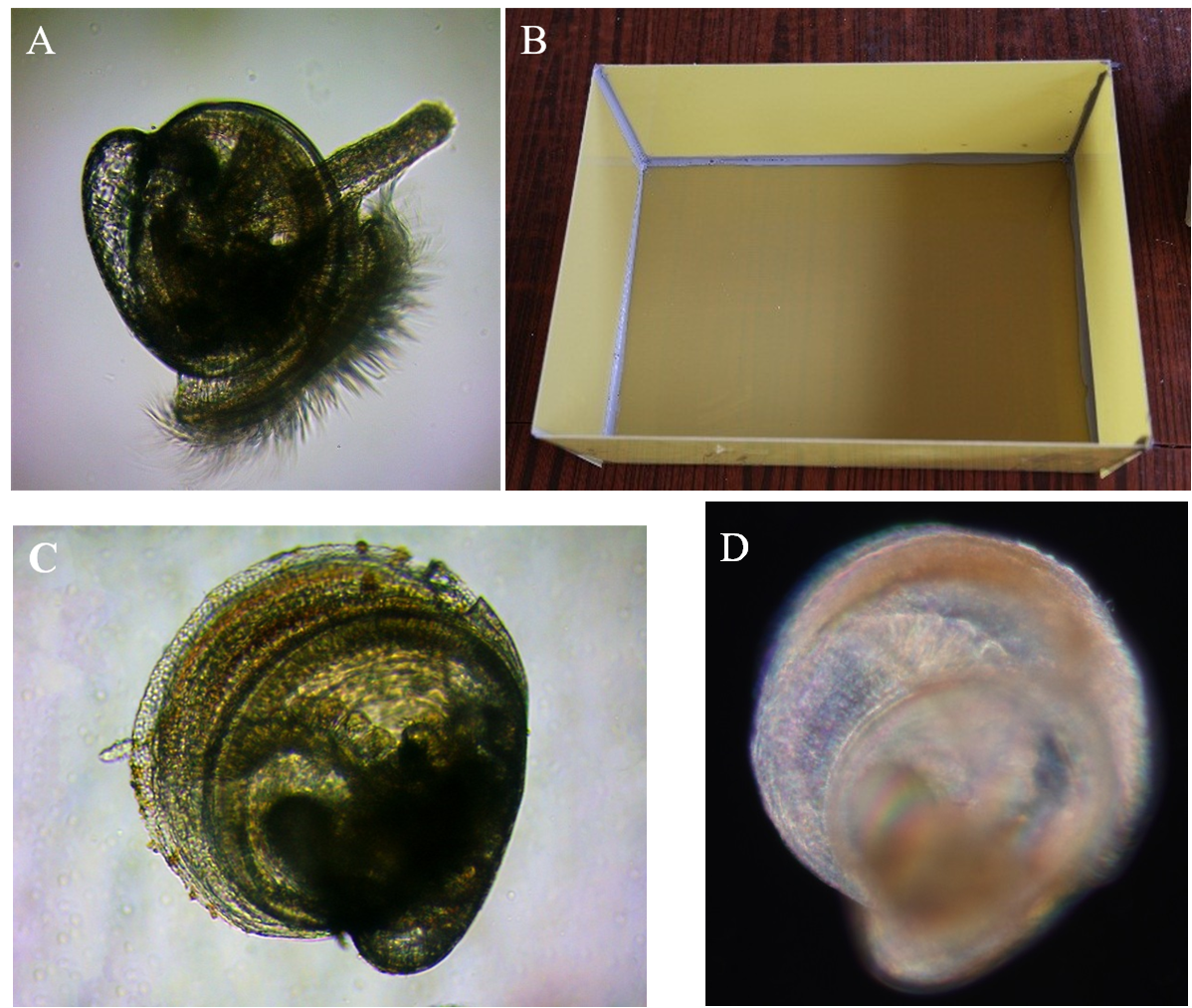

Supplement: Supplementary file 1 — Additional file 1: Supplementary Figure 1. The basic information of proteome identification. Supplementary Figure 2. The overall distribution of quantitative proteomics. A. Protein mass distribution; B. Peptide length distribution; C. Distribution of peptides’s sequence coverage; D. Distribution of unique peptide. Supplementary Figure 3. Gene ontology classification of oyster proteome. Supplementary Figure 4. KEGG function classification of oyster proteome. A-cellular processes, B-environmental information processing, C-genetic information processing, D-metabolism, E-organismal system. Supplementary Figure 5. Embryonic development of Fujian oyster Crassostrea angulate (A) Post eyespot larvae; (B) the collection box in Fujian oyster C. angulate; (C) the larvae after metamorphosis; (D) the larvae after metamorphosis induced by epinephrine. [file 12864_2020_7066_MOESM1_ESM.doc]
