# Supplementary material for: Proteome of larval metamorphosis induced by epinephrine in the Fujian oyster Crassostrea angulata
Source: BMC Genomics. 2020 Sep 29;21:675. doi: 10.1186/s12864-020-07066-z (PMC7525975; doi:10.1186/s12864-020-07066-z)
Supplement: Supplementary file 2 — Additional file 2: Supplementary Table 1. Compared with PL, High and low level expression for differentially abundant proteins in PA [file 12864_2020_7066_MOESM2_ESM.doc]

**Supplementary Table 1** Compared with PL, High and low level expression for differentially abundant proteins in PA

| **Accession Number** | **NR GI** | **Identified Proteins** | **Species** | **PA/PL** |
| --- | --- | --- | --- | --- |
|  | 405970417 | Titin | *Crassostrea gigas* | 35 |
|  | 405974270 | 5'-AMP-activated protein kinase subunit beta-2 | *Crassostrea gigas* | 3.3 |
|  | 405965820 | 60S ribosomal protein L26 | *Crassostrea gigas* | 2.2 |
| c85397_g1 | 762138531 | ATP synthase mitochondrial F1 complex assembly factor 2-like | *Crassostrea gigas* | 3.1 |
| c84804_g1 | 762131596 | ATP synthase subunit delta, mitochondrial-like | *Crassostrea gigas* | 2.1 |
|  | **405960423** | **Cadherin-23** | ***Crassostrea gigas*** | **4.9** |
| **c100397_g2** | **762110068** | **cadherin-23-like isoform X3** | ***Crassostrea gigas*** | **2.5** |
| **c101186_g1** | **762110074** | **cadherin-87A-like isoform X3** | ***Crassostrea gigas*** | **6.6** |
|  | **150404776** | **calreticulin** | ***Pinctada fucata*** | **2.1** |
|  | **405966500** | **Cathepsin L** | ***Crassostrea gigas*** | **2.2** |
| **c94508_g1** | **762107740** | **cathepsin L1-like** | ***Crassostrea gigas*** | **7.6** |
| **c90692_g1** | **762167480** | **cathepsin L1-like** | ***Crassostrea gigas*** | **2.3** |
| **c102687_g1** | **762092188** | **CD109 antigen-like** | ***Crassostrea gigas*** | **2.6** |
| c75751_g1 | 762149469 | chondroitin proteoglycan 2-like isoform X1 | *Crassostrea gigas* | 2.1 |
| c93420_g1 | 762126464 | cold shock domain-containing protein 3-like | *Crassostrea gigas* | 2.5 |
|  | 405954419 | Collagen alpha-3(VI) chain | *Crassostrea gigas* | 2.6 |
|  | 405975170 | CUB and sushi domain-containing protein 1 | *Crassostrea gigas* | 7.5 |
| c28847_g1 | 762072289 | cubilin-like | *Crassostrea gigas* | 5.6 |
| c101114_g1 | 762113070 | cystathionine beta-synthase-like isoform X6 | *Crassostrea gigas* | 2.1 |
|  | 405966262 | Deleted in malignant brain tumors 1 protein | *Crassostrea gigas* | 3.4 |
|  | 405976185 | Deleted in malignant brain tumors 1 protein | *Crassostrea gigas* | 2.8 |
|  | 405975234 | Dual oxidase 2, partial | *Crassostrea gigas* | 5 |
|  | 405969825 | E3 ubiquitin-protein ligase HUWE1 | *Crassostrea gigas* | 18 |
| c79422_g1 | 762115410 | EF-hand calcium-binding domain-containing protein 10-like | *Crassostrea gigas* | 2 |
| c92449_g2 | 762129765 | ER membrane protein complex subunit 10-like isoform X1 | *Crassostrea gigas* | 2.6 |
|  | 405966738 | Eukaryotic translation initiation factor 4 gamma 3 | *Crassostrea gigas* | 2.8 |
|  | 405975002 | Eukaryotic translation initiation factor 6 | *Crassostrea gigas* | 2.3 |
|  | 405968987 | Fatty acid-binding-like protein 5 | *Crassostrea gigas* | 2.4 |
|  | 405963678 | Fibropellin-1 | *Crassostrea gigas* | 3.7 |
|  | 405973352 | Glucose-repressible alcohol dehydrogenase transcriptional effector | *Crassostrea gigas* | 2 |
| c90196_g1 | 762099264 | glucosidase 2 subunit beta-like isoform X2 | *Crassostrea gigas* | 2.4 |
| c96570_g1 | 762134867 | glutathione reductase, mitochondrial-like | *Crassostrea gigas* | 6.9 |
| c89955_g1 | 762129389 | heat shock 70 kDa protein 14-like | *Crassostrea gigas* | 2 |
|  | 15004986 | integrin beta cgh | *Crassostrea gigas* | 2 |
| c100530_g1 | 762106758 | integrin beta pat-3-like | *Crassostrea gigas* | 21 |
| c101066_g1 | 762162532 | integrin beta-1-B-like | *Crassostrea gigas* | 3.3 |
|  | 405977054 | Kinesin heavy chain | *Crassostrea gigas* | 2 |
|  | **405972462** | **Kyphoscoliosis peptidase** | ***Crassostrea gigas*** | **3.8** |
| c103140_g1 | 762101975 | laccase-4-like | *Crassostrea gigas* | 2.1 |
|  | 405969732 | Laminin subunit alpha | *Crassostrea gigas* | 3.2 |
| c103776_g1 | 762109423 | laminin subunit alpha-like | *Crassostrea gigas* | 3.9 |
|  | 405950801 | La-related protein 1 | *Crassostrea gigas* | 2.1 |
|  | 405959794 | Leucine-zipper-like transcriptional regulator 1 | *Crassostrea gigas* | 2 |
|  | 405952731 | Lupus La-like protein | *Crassostrea gigas* | 9.5 |
| c95118_g1 | 762168992 | minus strand |  | 17 |
| c85400_g1 | 406817026 | minus strand |  | 2.3 |
| **c91752_g1** | **762100460** | **mucin-like protein** | ***Crassostrea gigas*** | **8.4** |
|  | 405975739 | Murinoglobulin-2 | *Crassostrea gigas* | 2.7 |
|  | 405967527 | Nesprin-1 | *Crassostrea gigas* | 3.3 |
| c101304_g1 | 762085332 | neural cell adhesion molecule 2-like isoform X7 | *Crassostrea gigas* | 2.3 |
|  | 405958312 | Neuroglian | *Crassostrea gigas* | 2.7 |
| c96718_g1 | 762143197 | neutral ceramidase-like | *Crassostrea gigas* | 2.5 |
| c94770_g1 | 762089386 | nidogen-1-like isoform X1 | *Crassostrea gigas* | 2.2 |
| c58989_g1 | 762081924 | nidogen-2-like | *Crassostrea gigas* | 2.4 |
|  | 405958470 | Papilin | *Crassostrea gigas* | 2.4 |
| c93257_g1 | 762136319 | phosphomannomutase-like isoform X1 | *Crassostrea gigas* | 2.3 |
| c83148_g1 | 762111593 | plasminogen activator inhibitor 1 RNA-binding protein-like isoform X1 | *Crassostrea gigas* | 2.3 |
|  | 405975722 | Polyamine-modulated factor 1-binding protein 1 | *Crassostrea gigas* | 5.2 |
| c90524_g1 | 762125425 | proliferating cell nuclear antigen-like | *Crassostrea gigas* | 2.2 |
|  | 405963677 | Protein VPRBP | *Crassostrea gigas* | 2.2 |
|  | 405950809 | Protocadherin Fat 4 | *Crassostrea gigas* | 3.1 |
| c115430_g1 | 762134719 | protocadherin Fat 4-like | *Crassostrea gigas* | 2.9 |
| c101457_g1 | 762080804 | protocadherin Fat 4-like isoform X3 | *Crassostrea gigas* | 2.4 |
|  | 405951930 | Protocadherin-like wing polarity protein stan | *Crassostrea gigas* | 2.2 |
| c95972_g1 | 762095264 | putative malate dehydrogenase 1B | *Crassostrea gigas* | 2 |
|  | 405978194 | Putative sulfite oxidase, mitochondrial | *Crassostrea gigas* | 2.1 |
| **c75090_g1** | **762085934** | **radial spoke head 1 homolog** | ***Crassostrea gigas*** | **2.3** |
|  | **405966926** | **SCO-spondin** | ***Crassostrea gigas*** | **7.8** |
| **c83004_g1** | **675373238** | **SCO-spondin, partial** | ***Stegodyphus mimosarum*** | **6.3** |
|  | 405951043 | Serine protease inhibitor dipetalogastin | *Crassostrea gigas* | 10 |
|  | 405953442 | Signal peptide, CUB and EGF-like domain-containing protein 1 | *Crassostrea gigas* | 2 |
| c99017_g1 | 762122989 | spectrin alpha chain-like isoform X6 | *Crassostrea gigas* | 2.1 |
|  | 405971538 | Synaptophysin [Crassostrea gigas |  | 2 |
|  | 405960104 | Tenascin-X | *Crassostrea gigas* | 9 |
|  | 405972180 | Tetratricopeptide repeat protein 25 | *Crassostrea gigas* | 2.7 |
| c90531_g1 | 762118303 | transforming growth factor-beta-induced protein ig-h3-like | *Crassostrea gigas* | 2.7 |
|  | 405976865 | Translocon-associated protein subunit alpha | *Crassostrea gigas* | 4.9 |
|  | 405967637 | Tropomyosin | *Crassostrea gigas* | 6 |
|  | 219806594 | tropomyosin | *Crassostrea gigas* | 4.5 |
|  | 375073719 | tropomyosin 1, partial | *Ostrea edulis* | 4.5 |
| c96730_g1 | 762108713 | ubiquilin-1-like | *Crassostrea gigas* | 2.3 |
|  | 405960135 | Voltage-dependent calcium channel subunit alpha-2/delta-2 | *Crassostrea gigas* | 2.4 |
|  | 405972713 | von Willebrand factor D and EGF domain-containing protein | *Crassostrea gigas* | 6.2 |
|  | 405969928 | von Willebrand factor D and EGF domain-containing protein | *Crassostrea gigas* | 5.8 |
| **c96390_g1** | **762145704** | **V-type proton ATPase subunit S1-like** | ***Crassostrea gigas*** | **8.4** |
|  | 405954463 | WD repeat-containing protein C10orf79 | *Crassostrea gigas* | 19 |
|  | 405966050 | Zinc finger RNA-binding protein | *Crassostrea gigas* | 6.5 |
|  | 405964878 | 40S ribosomal protein S11 | *Crassostrea gigas* | 0.3 |
| c85361_g1 | 762086880 | 4-hydroxyphenylpyruvate dioxygenase | *Crassostrea gigas* | 0 |
|  | 405976318 | 6-phosphogluconate dehydrogenase, decarboxylating | *Crassostrea gigas* | 0.4 |
|  | 405974071 | Actin | *Crassostrea gigas* | 0 |
|  | 405969755 | Actin-3 | *Crassostrea gigas* | 0.4 |
| c93725_g3 | 527271971 | acyl-CoA-binding protein | *Melopsittacus undulatus* | 0.2 |
|  | 405972978 | Adenylosuccinate synthetase | *Crassostrea gigas* | 0.3 |
|  | 405961802 | ADP-ribosylation factor | *Crassostrea gigas* | 0.5 |
| c99122_g1 | 762095292 | aldehyde dehydrogenase family 3 member B1-like | *Crassostrea gigas* | 0 |
| c90043_g1 | 762146639 | alpha-amylase-like | *Crassostrea gigas* | 0 |
|  | 405961891 | Alpha-crystallin B chain | *Crassostrea gigas* | 0.5 |
| c92241_g2 | 762101727 | alpha-L-fucosidase-like isoform X2 | *Crassostrea gigas* | 0.4 |
|  | 405954380 | Alpha-soluble NSF attachment protein | *Crassostrea gigas* | 0 |
|  | 405977952 | Aminopeptidase N | *Crassostrea gigas* | 0.4 |
|  | 405962570 | AP-2 complex subunit alpha-2 | *Crassostrea gigas* | 0.1 |
| c89235_g1 | 405951507 | AP-2 complex subunit mu-1 | *Crassostrea gigas* | 0 |
| c80501_g2 | 762073926 | arcoplasmic calcium-binding protein-like isoform X2 | *Crassostrea gigas* | 0.4 |
|  | 163311503 | ATP synthase F0 subunit 6 | *Crassostrea hongkongensis* | 0.3 |
| c90671_g1 | 762136815 | beta-catenin-like protein 1 | *Crassostrea gigas* | 0 |
|  | 405967658 | Bifunctional aminoacyl-tRNA synthetase | *Crassostrea gigas* | 0.4 |
|  | **405964165** | **Calcium/calmodulin-dependent protein kinase type II delta chain** | ***Crassostrea gigas*** | **0.1** |
|  | **405969211** | **Calcium-binding mitochondrial carrier protein Aralar1** | ***Crassostrea gigas*** | **0** |
|  | **405968450** | **Calcium-transporting ATPase sarcoplasmic/endoplasmic reticulum type** | ***Crassostrea gigas*** | **0.2** |
| **c90479_g1** | **762086942** | **calcium-transporting ATPase sarcoplasmic/endoplasmic reticulum type-like** | ***Crassostrea gigas*** | **0.2** |
|  | **20137620** | **Calmodulin; Short=CaM** |  | **0.5** |
| **c55559_g1** | **762161385** | **calmodulin-like** | ***Crassostrea gigas*** | **0** |
| **c97263_g1** | **405967580** | **Calnexin** | ***Crassostrea gigas*** | **0.5** |
| **c88952_g1** | **762104881** | **calumenin-like isoform X1** | ***Crassostrea gigas*** | **0** |
|  | 405953236 | Carbonic anhydrase | *Crassostrea gigas* | 0 |
| c97264_g1 | 762104782 | carbonic anhydrase 2-like | *Crassostrea gigas* | 0 |
|  | 405974400 | Carbonyl reductase[NADPH 1 | *Crassostrea gigas* | 0.05 |
| c91048_g1 | 762121724 | cell migration-inducing and hyaluronan-binding protein-like | *Crassostrea gigas* | 0 |
| **c75378_g1** | **762102752** | **cilia- and flagella-associated protein 20** | ***Crassostrea gigas*** | **0.4** |
|  | 405961982 | Collagen alpha-5(VI) chain | *Crassostrea gigas* | 0.1 |
|  | 405954309 | Constitutive coactivator of PPAR-gamma-like protein 1-like protein | *Crassostrea gigas* | 0.2 |
|  | 229324834 | cytochrome b | *Crassostrea angulata* | 0 |
|  | 187762792 | cytochrome c oxidase subunit 1 | *Crassostrea gigas* | 0.05 |
|  | 229324835 | cytochrome c oxidase subunit II | *Crassostrea angulata* | 0.4 |
|  | 405977373 | Dynein heavy chain 1, axonemal | *Crassostrea gigas* | 0.5 |
|  | 405966381 | Dynein heavy chain 6, axonemal | *Crassostrea gigas* | 0.08 |
|  | 405963852 | Dynein heavy chain 7, axonemal | *Crassostrea gigas* | 0.4 |
|  | 405969117 | Dynein heavy chain 7, axonemal | *Crassostrea gigas* | 0.3 |
| c101386_g1 | 762133698 | EF-hand calcium-binding domain-containing protein 5-like isoform X1 | *Crassostrea gigas* | 0 |
| c86615_g1 | 762130855 | EF-hand domain-containing family member C2-like | *Crassostrea gigas* | 0.3 |
| c82826_g1 | 762138085 | EF-hand domain-containing protein 1-like | *Crassostrea gigas* | 0.4 |
|  | 405971816 | Endoplasmic reticulum aminopeptidase 1 | *Crassostrea gigas* | 0.5 |
| c88602_g1 | 405975361 | eosinophil peroxidase-like isoform X2 | *Crassostrea gigas* | 0.2 |
| c100882_g1 | 762105167 | flotillin-1-like isoform X4 | *Crassostrea gigas* | 0.3 |
| c94435_g1 | 762097616 | flotillin-2a-like | *Crassostrea gigas* | 0.2 |
| c98882_g1 | 762080824 | galactokinase-like | *Crassostrea gigas* | 0.4 |
| c97708_g1 | 762072670 | GDP-L-fucose synthase-like | *Crassostrea gigas* | 0 |
| c99968_g1 | 762101323 | glucose-6-phosphate isomerase-like | *Crassostrea gigas* | 0 |
| c83840_g1 | 762070756 | glutathione S-transferase A-like | *Crassostrea gigas* | 0.2 |
|  | 56718386 | glycogen synthase | *Crassostrea gigas* | 0.4 |
| c101959_g1 | 405975684 | HEAT repeat-containing protein 7A | *Crassostrea gigas* | 0.3 |
|  | 405961245 | Heat shock 70 kDa protein 12B | *Crassostrea gigas* | 0.3 |
| **c67896_g1** | **762109657** | **hepatic lectin-like** | ***Crassostrea gigas*** | **0.5** |
|  | 405962319 | Histone H3 | *Crassostrea gigas* | 0.4 |
|  | 405963114 | Hydroxysteroid dehydrogenase-like protein 2 | *Crassostrea gigas* | 0.2 |
|  | 405969882 | Importin-7 | *Crassostrea gigas* | 0.1 |
| c87788_g2 | 762102409 | integrin alpha-6-like isoform X2 | *Crassostrea gigas* | 0 |
|  | 405972492 | Kinesin-related protein 1 | *Crassostrea gigas* | 0.4 |
| c101403_g2 | 405958866 | Lachesin | *Crassostrea gigas* | 0 |
|  | 405963229 | Laminin subunit gamma-1 | *Crassostrea gigas* | 0.3 |
| c95819_g1 | 405977265 | Long-chain specific acyl-CoA dehydrogenase, mitochondrial | *Crassostrea gigas* | 0.4 |
| c87967_g1 | 762115439 | mannose-6-phosphate isomerase-like | *Crassostrea gigas* | 0.4 |
| **c100749_g1** | **762156704** | **MAP kinase-activated protein kinase 2-like** | ***Crassostrea gigas*** | **0.4** |
|  | 405974809 | Metabotropic glutamate receptor 3 | *Crassostrea gigas* | 0.2 |
| c93709_g2 | 762164091 | MICOS complex subunit Mic60-like isoform X1 | *Crassostrea gigas* | 0.4 |
| c95340_g1 | 762099550 | minus strand |  | 0.5 |
| c98275_g1 | 665815290 | minus strand |  | 0.4 |
| c98973_g1 | 762109068 | multidrug resistance-associated protein 1-like isoform X1 | *Crassostrea gigas* | 0.2 |
|  | 405975835 | NAD(P) transhydrogenase, mitochondrial | *Crassostrea gigas* | 0.5 |
| c91009_g1 | 762070002 | NADPH--cytochrome P450 reductase-like | *Crassostrea gigas* | 0.3 |
|  | 405964679 | Neurexin-4 | *Crassostrea gigas* | 0 |
|  | 405960111 | Neuroglian | *Crassostrea gigas* | 0 |
| c102881_g1 | 762129353 | neuroglian-like isoform X1 | *Crassostrea gigas* | 0.4 |
|  | 405966986 | Paramyosin | *Crassostrea gigas* | 0.4 |
| c94503_g2 | 762070443 | pathogen-related protein-like | *Crassostrea gigas* | 0 |
|  | **405962230** | **Peroxidasin** | ***Crassostrea gigas*** | **0.05** |
|  | **405962229** | **Peroxidasin-like protein** | ***Crassostrea gigas*** | **0.3** |
|  | **405977917** | **Peroxisomal multifunctional enzyme type 2** | ***Crassostrea gigas*** | **0.3** |
| **c93516_g1** | **762084138** | **peroxisomal multifunctional enzyme type 2-like** | ***Crassostrea gigas*** | **0.2** |
| c90267_g1 | 762070867 | phosphoenolpyruvate carboxykinase, cytosolic[GTP-like isoform X1 | *Crassostrea gigas* | 0.3 |
| c95528_g1 | 762121240 | PREDICTED: acetyl-CoA acetyltransferase, mitochondrial-like | *Crassostrea gigas* | 0.5 |
|  | 405965891 | Prenylcysteine oxidase | *Crassostrea gigas* | 0 |
|  | 405968717 | Programmed cell death protein 6 | *Crassostrea gigas* | 0.4 |
| c91045_g2 | 762163372 | protein disulfide-isomerase A5-like | *Crassostrea gigas* | 0.5 |
|  | 405965843 | Protein ERGIC-53 | *Crassostrea gigas* | 0.5 |
|  | 405965662 | Protein lap4 | *Crassostrea gigas* | 0 |
|  | 405962160 | Protocadherin Fat 4 | *Crassostrea gigas* | 0.2 |
|  | **333449487** | **Ras-like GTP-binding protein RHO** | ***Crassostrea ariakensis*** | **0.5** |
| **c92890_g1** | **762144541** | **ras-like protein 3 isoform X2** | ***Crassostrea gigas*** | **0.5** |
|  | **405976260** | **Ras-related protein Rab-10** | ***Crassostrea gigas*** | **0.5** |
| **c97767_g2** | **762121318** | **ras-related protein Rab-14** | ***Crassostrea gigas*** | **0.5** |
| **c83633_g1** | **762141921** | **ras-related protein Rab-35-like** | ***Crassostrea gigas*** | **0.2** |
|  | **405978849** | **Rho GTPase-activating protein 17** | ***Crassostrea gigas*** | **0** |
|  | 405962126 | rRNA 2'-O-methyltransferase fibrillarin | *Crassostrea gigas* | 0 |
| **c96209_g1** | **307197748** | **Ryanodine receptor 44F** | ***Harpegnathos saltator*** | **0.2** |
| c80501_g1 | 405963560 | Sarcoplasmic calcium-binding protein | *Crassostrea gigas* | 0.3 |
|  | 405973087 | SH3 domain-binding glutamic acid-rich protein | *Crassostrea gigas* | 0.03 |
|  | 405976087 | Splicing factor U2AF 50 kDa subunit | *Crassostrea gigas* | 0.2 |
|  | 405968979 | Steroid 17-alpha-hydroxylase/17,20 lyase | *Crassostrea gigas* | 0.3 |
|  | 405955028 | Steroid 17-alpha-hydroxylase/17,20 lyase | *Crassostrea gigas* | 0.2 |
| c85806_g1 | 762100869 | succinate dehydrogenase cytochrome b560 subunit, mitochondrial-like isoform X1 | *Crassostrea gigas* | 0.5 |
| **c78713_g1** | **821595281** | **superoxide dismutase[Mn, mitochondrial-like** | ***Crassostrea gigas*** | **0.2** |
|  | 405970435 | Thioredoxin domain-containing protein 5 | *Crassostrea gigas* | 0 |
|  | 405974168 | Titin | *Crassostrea gigas* | 0.4 |
|  | 405957915 | Transmembrane protein 2 | *Crassostrea gigas* | 0.1 |
| c81034_g1 | 405970776 | Troponin C | *Crassostrea gigas* | 0 |
| **c86426_g1** | **762094049** | **universal stress protein A-like protein** | ***Crassostrea gigas*** | **0.4** |
| **c83738_g1** | **762091580** | **universal stress protein A-like protein** | ***Crassostrea gigas*** | **0.3** |
| **c89143_g1** | **762091586** | **universal stress protein A-like protein** | ***Crassostrea gigas*** | **0.2** |
| **c92715_g1** | **762091592** | **universal stress protein A-like protein isoform X2** | ***Crassostrea gigas*** | **0.3** |
| c91906_g2 | 762098971 | UPF0573 protein C2orf70 homolog A-like | *Crassostrea gigas* | 0.4 |
| c101861_g1 | 762144091 | vesicle-fusing ATPase 1-like | *Crassostrea gigas* | 0.3 |
|  | 405968088 | WD repeat-containing protein 19 | *Crassostrea gigas* | 0.2 |
