# Supplementary material for: Proteome of larval metamorphosis induced by epinephrine in the Fujian oyster Crassostrea angulata
Source: BMC Genomics. 2020 Sep 29;21:675. doi: 10.1186/s12864-020-07066-z (PMC7525975; doi:10.1186/s12864-020-07066-z)
Supplement: Supplementary file 3 — Additional file 3: Supplementary Table 2. Compared with PA, High and low level expression for differentially abundant proteins in MET [file 12864_2020_7066_MOESM3_ESM.doc]

**Supplementary Table 2** Compared with PA, High and low level expression for differentially abundant proteins in MET

| **Accession Number** | **NR GI** | **Identified Proteins** | **Species** | **MET/PA** |
| --- | --- | --- | --- | --- |
| c99968_g1 | 762101323 | glucose-6-phosphate isomerase-like | *Crassostrea gigas* | INF |
|  | 405962781 | 26S proteasome non-ATPase regulatory subunit 3 | *Crassostrea gigas* | 2.3 |
|  | 405955617 | 3-hydroxyanthranilate 3,4-dioxygenase | *Crassostrea gigas* | 2.1 |
| c85361_g1 | 762086880 | 4-hydroxyphenylpyruvate dioxygenase | *Crassostrea gigas* | INF |
| c98089_g1 | 762110625 | A disintegrin and metalloproteinase with thrombospondin motifs 6-like | *Crassostrea gigas* | 2.3 |
|  | 405958039 | Acidic leucine-rich nuclear phosphoprotein 32 family member A | *Crassostrea gigas* | 2.1 |
|  | 405974071 | Actin | *Crassostrea gigas* | INF |
|  | 405969755 | Actin-3 | *Crassostrea gigas* | 4.3 |
| c99122_g1 | 762095292 | aldehyde dehydrogenase family 3 member B1-like | *Crassostrea gigas* | INF |
| c90043_g1 | 762146639 | alpha-amylase-like | *Crassostrea gigas* | INF |
| c92241_g2 | 762101727 | alpha-L-fucosidase-like isoform X2 | *Crassostrea gigas* | 2.4 |
|  | 405954380 | Alpha-soluble NSF attachment protein | *Crassostrea gigas* | INF |
|  | 405977952 | Aminopeptidase N | *Crassostrea gigas* | 5.2 |
|  | 405961595 | Anosmin-1 | *Crassostrea gigas* | 2.1 |
| c89235_g1 | 405951507 | AP-2 complex subunit mu-1 | *Crassostrea gigas* | INF |
| c90671_g1 | 762136815 | beta-catenin-like protein 1 | *Crassostrea gigas* | INF |
|  | 405965163 | Beta-hexosaminidase subunit beta | *Crassostrea gigas* | 2.1 |
|  | **405960423** | **Cadherin-23** | ***Crassostrea gigas*** | **2.2** |
|  | **405969211** | **Calcium-binding mitochondrial carrier protein Aralar1** | ***Crassostrea gigas*** | **INF** |
|  | **405968450** | **Calcium-transporting ATPase sarcoplasmic/endoplasmic reticulum type** | ***Crassostrea gigas*** | **2.8** |
| **c90479_g1** | **762086942** | **calcium-transporting ATPase sarcoplasmic/endoplasmic reticulum type-like** | ***Crassostrea gigas*** | **3.9** |
|  | **20137620** | **Calmodulin; Short=CaM** |  | **2.5** |
| **c55559_g1** | **762161385** | **calmodulin-like** | ***Crassostrea gigas*** | **INF** |
| **c97263_g1** | **405967580** | **Calnexin** | ***Crassostrea gigas*** | **3.4** |
| **c93407_g1** | **762076798** | **caltractin-like** | ***Crassostrea gigas*** | **2** |
| **c88952_g1** | **762104881** | **calumenin-like isoform X1** | ***Crassostrea gigas*** | **INF** |
| **c98322_g1** | **762079826** | **calumenin-like isoform X1** | ***Crassostrea gigas*** | **2.6** |
|  | 405953236 | Carbonic anhydrase | *Crassostrea gigas* | INF |
| c97264_g1 | 762104782 | carbonic anhydrase 2-like | *Crassostrea gigas* | INF |
|  | 405974400 | Carbonyl reductase[NADPH 1] | *Crassostrea gigas* | 6.6 |
| **c88094_g1** | **762099884** | **cathepsin L1-like** | ***Crassostrea gigas*** | **2** |
| c91048_g1 | 762121724 | cell migration-inducing and hyaluronan-binding protein-like | *Crassostrea gigas* | INF |
|  | **405954419** | **Collagen alpha-3(VI) chain** | ***Crassostrea gigas*** | **4.7** |
|  | **405961982** | **Collagen alpha-5(VI) chain** | ***Crassostrea gigas*** | **18** |
|  | 229324834 | cytochrome b | *Crassostrea angulata* | INF |
|  | 187762792 | cytochrome c oxidase subunit 1 | *Crassostrea gigas* | 4.1 |
|  | 405966262 | Deleted in malignant brain tumors 1 protein | *Crassostrea gigas* | 3.8 |
|  | 405965494 | DnaJ-like protein subfamily B member 11 | *Crassostrea gigas* | 5.3 |
| **c90254_g1** | **762155518** | **drebrin-like protein B isoform X2** | ***Crassostrea gigas*** | **19** |
| c101386_g1 | 762133698 | EF-hand calcium-binding domain-containing protein 5-like isoform X1 | *Crassostrea gigas* | INF |
|  | 405968675 | EF-hand domain-containing protein D1 | *Crassostrea gigas* | 2.2 |
|  | 405971816 | Endoplasmic reticulum aminopeptidase 1 | *Crassostrea gigas* | 6.4 |
| c100882_g1 | 762105167 | flotillin-1-like isoform X4 | *Crassostrea gigas* | 2 |
| c94435_g1 | 762097616 | flotillin-2a-like | *Crassostrea gigas* | 2.7 |
| c100029_g2 | 762099370 | gastric intrinsic factor-like | *Crassostrea gigas* | 2.1 |
| c97708_g1 | 762072670 | GDP-L-fucose synthase-like | *Crassostrea gigas* | INF |
|  | 405973352 | Glucose-repressible alcohol dehydrogenase transcriptional effector | *Crassostrea gigas* | 4.5 |
| **c101959_g1** | **405975684** | **HEAT repeat-containing protein 7A** | ***Crassostrea gigas*** | **2.6** |
| **c89955_g1** | **762129389** | **heat shock 70 kDa protein 14-like** | ***Crassostrea gigas*** | **2.4** |
| **c82792_g1** | **762131241** | **heat shock protein 27-like** | ***Crassostrea gigas*** | **5.8** |
|  | 405978261 | Hexokinase type 2 | *Crassostrea gigas* | 2.2 |
|  | 405963114 | Hydroxysteroid dehydrogenase-like protein 2 | *Crassostrea gigas* | 6.8 |
| **c94262_g1** | **762101734** | **IgGFc-binding protein-like** | ***Crassostrea gigas*** | **2.9** |
|  | 405969882 | Importin-7 | *Crassostrea gigas* | 2.6 |
| **c87788_g2** | **762102409** | **integrin alpha-6-like isoform X2** | ***Crassostrea gigas*** | **INF** |
| **c101066_g1** | **762162532** | **integrin beta-1-B-like** | ***Crassostrea gigas*** | **2.2** |
| **c95812_g1** | **762147513** | **interferon-induced protein 44-like** | ***Crassostrea gigas*** | **2.2** |
| c101403_g2 | 405958866 | Lachesin | *Crassostrea gigas* | INF |
|  | 405969732 | Laminin subunit alpha | *Crassostrea gigas* | 2.5 |
| c103776_g1 | 762109423 | laminin subunit alpha-like | *Crassostrea gigas* | 2.1 |
|  | 405963229 | Laminin subunit gamma-1 | *Crassostrea gigas* | 29 |
| **c99479_g1** | **762118717** | **lysosome-associated membrane glycoprotein 1-like isoform X2** | ***Crassostrea gigas*** | **2.1** |
|  | 405965903 | Major egg antigen | *Crassostrea gigas* | 3.6 |
|  | 405959230 | Membrane metallo-endopeptidase-like 1 | *Crassostrea gigas* | 4 |
|  | 405974809 | Metabotropic glutamate receptor 3 | *Crassostrea gigas* | 11 |
|  | 405968797 | Methenyltetrahydrofolate synthetase domain-containing protein | *Crassostrea gigas* | 2.2 |
| c93709_g2 | 762164091 | MICOS complex subunit Mic60-like isoform X1 | *Crassostrea gigas* | 2.3 |
| c93185_g1 | 405950468 | minus strand |  | 2.8 |
| c85400_g1 | 406817026 | minus strand |  | 2.1 |
| **c95895_g1** | **762099838** | **mucin-5AC-like** | ***Crassostrea gigas*** | **5.4** |
| **c102473_g1** | **762076941** | **mucin-5AC-like** | ***Crassostrea gigas*** | **3.5** |
|  | 405970698 | Multidrug resistance protein 1 | *Crassostrea gigas* | 5.2 |
|  | 405956360 | Multidrug resistance protein 1, partial | *Crassostrea gigas* | 11 |
| c98973_g1 | 762109068 | multidrug resistance-associated protein 1-like isoform X1 | *Crassostrea gigas* | 3.1 |
|  | 405975835 | NAD(P) transhydrogenase, mitochondrial | *Crassostrea gigas* | 2.1 |
| **c101658_g2** | **762095530** | **neural-cadherin-like** | ***Crassostrea gigas*** | **2.5** |
|  | **405964679** | **Neurexin-4** | ***Crassostrea gigas*** | **INF** |
|  | **405960111** | **Neuroglian** | ***Crassostrea gigas*** | **INF** |
|  | **405958312** | **Neuroglian** | ***Crassostrea gigas*** | **2.5** |
| c94770_g1 | 762089386 | nidogen-1-like isoform X1 | *Crassostrea gigas* | 2.2 |
|  | 405958470 | Papilin | *Crassostrea gigas* | 2.1 |
|  | 405966986 | Paramyosin | *Crassostrea gigas* | 3.2 |
| c94503_g2 | 762070443 | pathogen-related protein-like | *Crassostrea gigas* | INF |
|  | 405960428 | PDZ and LIM domain protein 1 | *Crassostrea gigas* | 2.9 |
|  | 405970466 | Periostin | *Crassostrea gigas* | 2 |
|  | 405969430 | Plasma alpha-L-fucosidase | *Crassostrea gigas* | 2.3 |
|  | 405965891 | Prenylcysteine oxidase | *Crassostrea gigas* | INF |
|  | 405960660 | Protein disulfide-isomerase A5 | *Crassostrea gigas* | 2 |
| c91045_g2 | 762163372 | protein disulfide-isomerase A5-like | *Crassostrea gigas* | 4.9 |
|  | 405965843 | Protein ERGIC-53 | *Crassostrea gigas* | 5.5 |
|  | 405962525 | Protein FAM63B | *Crassostrea gigas* | 2.6 |
| c78719_g1 | 762131783 | protein kinase C and casein kinase substrate in neurons protein 1-like isoform X1 | *Crassostrea gigas* | 2.2 |
|  | 405965662 | Protein lap4 | *Crassostrea gigas* | INF |
|  | 405978194 | Putative sulfite oxidase, mitochondrial | *Crassostrea gigas* | 3.5 |
|  | 405957461 | Putative thiopurine S-methyltransferase | *Crassostrea gigas* | 2.5 |
| **c95388_g1** | **762105818** | **ran-specific GTPase-activating protein-like** | ***Crassostrea gigas*** | **2.1** |
|  | **333449487** | **Ras-like GTP-binding protein RHO** | ***Crassostrea ariakensis*** | **3.2** |
|  | **405978849** | **Rho GTPase-activating protein 17** | ***Crassostrea gigas*** | **INF** |
|  | 405962126 | rRNA 2'-O-methyltransferase fibrillarin | *Crassostrea gigas* | INF |
|  | 405972994 | SEC13-like protein | *Crassostrea gigas* | 2.2 |
|  | 405973087 | SH3 domain-binding glutamic acid-rich protein | *Crassostrea gigas* | 18 |
|  | 405976087 | Splicing factor U2AF 50 kDa subunit | *Crassostrea gigas* | 4.8 |
|  | 405950905 | Syntaxin-5 | *Crassostrea gigas* | 2 |
|  | 405952896 | Tectonic-3 | *Crassostrea gigas* | 2.5 |
|  | 405970435 | Thioredoxin domain-containing protein 5 | *Crassostrea gigas* | INF |
| c96033_g1 | 762085768 | thioredoxin-like protein 1 | *Crassostrea gigas* | 3 |
| c90531_g1 | 762118303 | transforming growth factor-beta-induced protein ig-h3-like | *Crassostrea gigas* | 2.3 |
|  | 405976865 | Translocon-associated protein subunit alpha | *Crassostrea gigas* | 2.4 |
|  | 405957915 | Transmembrane protein 2 | *Crassostrea gigas* | 7.9 |
| c81034_g1 | 405970776 | Troponin C | *Crassostrea gigas* | INF |
|  | 405976987 | Tyrosine-protein phosphatase Lar | *Crassostrea gigas* | 6.9 |
|  | 405963822 | UPF0663 transmembrane protein C17orf28 | *Crassostrea gigas* | 3.4 |
| c101543_g1 | 762168651 | vigilin-like isoform X1 | *Crassostrea gigas Vigilin* | 2.5 |
| c102546_g1 | 762087607 | vinculin-like isoform X7 | *Crassostrea gigas* | 2.2 |
|  | 405972713 | von Willebrand factor D and EGF domain-containing protein | *Crassostrea gigas* | 2 |
| c93514_g1 | 762076444 | xylose isomerase-like | *Crassostrea gigas* | 3 |
|  | 405969358 | ATPase family AAA domain-containing protein 2B | *Crassostrea gigas* | 0.5 |
|  | 405966858 | 3-hydroxyacyl-CoA dehydrogenase type-2 | *Crassostrea gigas* | 0.4 |
|  | 405960426 | 4-aminobutyrate aminotransferase, mitochondrial | *Crassostrea gigas* | 0.1 |
|  | 405965820 | 60S ribosomal protein L26 | *Crassostrea gigas* | 0 |
|  | 405965901 | 60S ribosomal protein L27a | *Crassostrea gigas* | 0 |
|  | 405959119 | 60S ribosomal protein L3, partial | *Crassostrea gigas* | 0.2 |
| c93725_g3 | 527271971 | acyl-CoA-binding protein,Melopsittacus undulatus |  | 0 |
|  | 405972978 | Adenylosuccinate synthetase | *Crassostrea gigas* | 0 |
| c101293_g1 | 762096787 | alanine aminotransferase 1-like | *Crassostrea gigas* | 0 |
|  | 405962156 | Alpha-aminoadipic semialdehyde synthase, mitochondrial | *Crassostrea gigas* | 0.2 |
|  | 405976514 | Amyloid protein-binding protein 2 | *Crassostrea gigas* | 0 |
|  | 405962570 | AP-2 complex subunit alpha-2 | *Crassostrea gigas* | 0.3 |
| **c93079_g1** | **762165574** | **apoptosis-inducing factor 3-like isoform X1** | ***Crassostrea gigas*** | **0.2** |
| c85397_g1 | 762138531 | ATP synthase mitochondrial F1 complex assembly factor 2-like | *Crassostrea gigas* | 0.1 |
| c103615_g1 | 762111269 | ATP-citrate synthase-like isoform X1 | *Crassostrea gigas* | 0 |
|  | 405975706 | Band 4.1-like protein 3 | *Crassostrea gigas* | 0.4 |
|  | 533221120 | beta-mannosidase,Stenotrophomonas maltophilia MF89 |  | 0.4 |
|  | **405964165** | **Calcium/calmodulin-dependent protein kinase type II delta chain** | ***Crassostrea gigas*** | **0** |
|  | **405972360** | **Calpain-7-like protein** | ***Crassostrea gigas*** | **0** |
|  | **405959835** | **Catalase** | ***Crassostrea gigas*** | **0.4** |
| **c91059_g3** | **762099416** | **chitinase-3-like protein 1 isoform X3** | ***Crassostrea gigas*** | **0.3** |
| c75751_g1 | 762149469 | chondroitin proteoglycan 2-like isoform X1 | *Crassostrea gigas* | 0.5 |
| **c75378_g1** | **762102752** | **cilia- and flagella-associated protein 20** | ***Crassostrea gigas*** | **0.4** |
| **c99303_g1** | **762156177** | **cilia- and flagella-associated protein 61-like** | ***Crassostrea gigas*** | **0** |
|  | 405959610 | Coiled-coil domain-containing protein 81 | *Crassostrea gigas* | 0 |
|  | 405954309 | Constitutive coactivator of PPAR-gamma-like protein 1-like protein | *Crassostrea gigas* | 0.2 |
|  | 405950302 | Cystatin-A | *Crassostrea gigas* | 0.5 |
| c77693_g1 | 765826145 | cytochrome b-c1 complex subunit Rieske, mitochondrial-like | *Crassostrea gigas* | 0.2 |
|  | 405951857 | Cytochrome c1, heme protein, mitochondrial | *Crassostrea gigas* | 0.5 |
|  | 405953044 | Dynein beta chain, ciliary | *Crassostrea gigas* | 0.2 |
|  | 405976574 | Dynein beta chain, ciliary | *Crassostrea gigas* | 0.1 |
|  | 405977373 | Dynein heavy chain 1, axonemal | *Crassostrea gigas* | 0 |
|  | 405950869 | Dynein heavy chain 3, axonemal | *Crassostrea gigas* | 0.03 |
|  | 405971451 | Dynein heavy chain 5, axonemal | *Crassostrea gigas* | 0 |
|  | 405966381 | Dynein heavy chain 6, axonemal | *Crassostrea gigas* | 0 |
|  | 405969117 | Dynein heavy chain 7, axonemal | *Crassostrea gigas* | 0 |
|  | 405963852 | Dynein heavy chain 7, axonemal | *Crassostrea gigas* | 0 |
|  | 405969825 | E3 ubiquitin-protein ligase HUWE1 | *Crassostrea gigas* | 0.1 |
| c79422_g1 | 762115410 | EF-hand calcium-binding domain-containing protein 10-like | *Crassostrea gigas* | 0 |
|  | 405963739 | EF-hand calcium-binding domain-containing protein 6 | *Crassostrea gigas* | 0 |
| c86615_g1 | 762130855 | EF-hand domain-containing family member C2-like | *Crassostrea gigas* | 0.03 |
| c82826_g1 | 762138085 | EF-hand domain-containing protein 1-like | *Crassostrea gigas* | 0.07 |
|  | 405965462 | Elongation factor Tu, mitochondrial | *Crassostrea gigas* | 0.5 |
| c88602_g1 | 405975361 | eosinophil peroxidase-like isoform X2 | *Crassostrea gigas* | 0 |
| c92454_g1 | 762156349 | F-box only protein 21-like | *Crassostrea gigas* | 0.4 |
| c87781_g1 | 762131945 | F-box only protein 36-like | *Crassostrea gigas* | 0.1 |
| c83242_g1 | 762141095 | F-box/LRR-repeat protein 3-like | *Crassostrea gigas* | 0.3 |
|  | 405960295 | Fumarylacetoacetase | *Crassostrea gigas* | 0.3 |
| c89917_g1 | 762163284 | galectin-9-like isoform X2 | *Crassostrea gigas* | 0.4 |
| c92723_g1 | 762074236 | gelsolin-like protein 2 | *Crassostrea gigas* | 0.5 |
|  | 405959171 | Glucose-6-phosphate 1-dehydrogenase | *Crassostrea gigas* | 0 |
|  | 405969003 | Glutamate synthase,NADH, amyloplastic | *Crassostrea gigas* | 0.4 |
| c96570_g1 | 762134867 | glutathione reductase, mitochondrial-like | *Crassostrea gigas* | 0.3 |
| c102113_g1 | 762136488 | glycerol-3-phosphate dehydrogenase, mitochondrial-like isoform X2 | *Crassostrea gigas* | 0 |
|  | 405965040 | Glycogen phosphorylase, muscle form | *Crassostrea gigas* | 0.5 |
| c102976_g1 | 762100833 | glycogen phosphorylase, muscle form-like isoform X1 | *Crassostrea gigas* | 0.5 |
| c86623_g1 | 762093198 | growth factor receptor-bound protein 2-like isoform X1 | *Crassostrea gigas* | 0.4 |
|  | 405952109 | Hemicentin-1 | *Crassostrea gigas* | 0.09 |
|  | 405969689 | Hydrocephalus-inducing-like protein | *Crassostrea gigas* | 0 |
|  | 405963851 | IQ and ubiquitin-like domain-containing protein | *Crassostrea gigas* | 0 |
| **c86938_g1** | **405953294** | **Kyphoscoliosis peptidase** | ***Crassostrea gigas*** | **0.5** |
| c95118_g1 | 762168992 | minus strand |  | 0.2 |
| c78564_g1 | 405973457 | minus strand |  | 0.09 |
| **c97912_g1** | **762107124** | **mitogen-activated protein kinase 1-like** | ***Crassostrea gigas*** | **0** |
| **c11357_g1** | **762080102** | **mucin-19-like** | ***Crassostrea gigas*** | **0** |
| **c91752_g1** | **762100460** | **mucin-like protein** | ***Crassostrea gigas*** | **0** |
|  | 405965726 | NADP-dependent malic enzyme | *Crassostrea gigas* | 0.4 |
| c91009_g1 | 762070002 | NADPH--cytochrome P450 reductase-like | *Crassostrea gigas* | 0 |
|  | 405967527 | Nesprin-1 | *Crassostrea gigas* | 0.4 |
| c88012_g1 | 762111301 | Neuronal acetylcholine receptor subunit alpha-10 | *Crassostrea gigas* | 0 |
|  | 405951454 | Outer dense fiber protein 3 | *Crassostrea gigas* | 0.04 |
|  | 405952329 | PAB-dependent poly(A)-specific ribonuclease subunit 2 | *Crassostrea gigas* | 0.3 |
|  | 405962230 | Peroxidasin | *Crassostrea gigas* | 0 |
|  | 405962229 | Peroxidasin-like protein | *Crassostrea gigas* | 0 |
|  | **405977917** | **Peroxisomal multifunctional enzyme type 2** | ***Crassostrea gigas*** | **0** |
| **c93516_g1** | **762084138** | **peroxisomal multifunctional enzyme type 2-like** | ***Crassostrea gigas*** | **0** |
| **c87836_g1** | **762127059** | **Peroxisomal NADH pyrophosphatase NUDT12** | ***Crassostrea gigas*** | **0.5** |
| c80872_g1 | 762086921 | phosphoenolpyruvate phosphomutase | *Crassostrea gigas* | 0.5 |
| c97751_g1 | 762070302 | piwi-like protein 1 isoform X1 | *Crassostrea gigas* | 0.5 |
|  | 405958107 | Poly,ADP-ribose polymerase 1 | *Crassostrea gigas* | 0 |
|  | 405975722 | Polyamine-modulated factor 1-binding protein 1 | *Crassostrea gigas* | 0.3 |
|  | 405969398 | Protein jagged-2 | *Crassostrea gigas* | 0.4 |
|  | 405972892 | Protein SPATIAL | *Crassostrea gigas* | 0.08 |
|  | 405951163 | Putative adenylate kinase-like protein C9orf98-like protein | *Crassostrea gigas* | 0 |
| c95972_g1 | 762095264 | putative malate dehydrogenase 1B | *Crassostrea gigas* | 0 |
|  | 405953655 | Pyruvate dehydrogenase E1 component subunit alpha type II, mitochondrial | *Crassostrea gigas* | 0.4 |
| **c75090_g1** | **762085934** | **radial spoke head 1 homolog** | ***Crassostrea gigas*** | **0.2** |
| **c70729_g1** | **762119151** | **radial spoke head protein 4 homolog A-like** | ***Crassostrea gigas*** | **0.5** |
| **c76439_g1** | **762133006** | **radial spoke head protein 9 homolog** | ***Crassostrea gigas*** | **0.5** |
| **c97767_g2** | **762121318** | **ras-related protein Rab-14** | ***Crassostrea gigas*** | **0.3** |
|  | 405972837 | Retinal dehydrogenase 1 | *Crassostrea gigas* | 0.3 |
| c103590_g1 | 762100293 | rootletin-like isoform X6 | *Crassostrea gigas* | 0.3 |
| **c96209_g1** | **307197748** | **Ryanodine receptor 44F** | ***Harpegnathos saltator*** | **0** |
|  | 405967048 | Scaffold attachment factor B1 | *Crassostrea gigas* | 0 |
|  | 405964168 | Serine/threonine-protein phosphatase 2B catalytic subunit alpha isoform | *Crassostrea gigas* | 0.2 |
| **c86843_g1** | **762100962** | **soma ferritin-like** | ***Crassostrea gigas*** | **0.5** |
|  | 405969074 | Spermatogenesis-associated protein 18-like protein | *Crassostrea gigas* | 0.5 |
|  | 405955028 | Steroid 17-alpha-hydroxylase/17,20 lyase | *Crassostrea gigas* | 0.2 |
|  | 405968979 | Steroid 17-alpha-hydroxylase/17,20 lyase | *Crassostrea gigas* | 0 |
| c85806_g1 | 762100869 | succinate dehydrogenase cytochrome b560 subunit, mitochondrial-like isoform X1 | *Crassostrea gigas* | 0 |
| c86457_g1 | 762076524 | succinate dehydrogenase,ubiquinone iron-sulfur subunit, mitochondrial-like | *Crassostrea gigas* | 0.2 |
|  | 405959519 | Succinate-semialdehyde dehydrogenase, mitochondrial | *Crassostrea gigas* | 0.5 |
|  | 405963331 | Succinyl-CoA ligase[ADP-forming subunit beta, mitochondrial | *Crassostrea gigas* | 0.5 |
| **c78713_g1** | **821595281** | **superoxide dismutase,Mn, mitochondrial-like** | ***Crassostrea gigas*** | **0** |
| c99665_g2 | 405965400 | Synaptopodin-2 | *Crassostrea gigas* | 0.3 |
|  | 405953549 | T-complex protein 1 subunit zeta | *Crassostrea gigas* | 0.2 |
| c80732_g1 | 762169708 | tektin-2-like | *Crassostrea gigas* | 0.2 |
|  | 405975469 | Tektin-3 | *Crassostrea gigas* | 0.1 |
| c89247_g1 | 762135468 | tektin-3-like isoform X2 | *Crassostrea gigas* | 0.2 |
|  | 405972180 | Tetratricopeptide repeat protein 25 | *Crassostrea gigas* | 0.3 |
| c98785_g1 | 762080959 | thioredoxin reductase 1, cytoplasmic-like | *Crassostrea gigas* | 0.5 |
|  | 405974168 | Titin | *Crassostrea gigas* | 0.5 |
|  | 405966231 | Transmembrane protein 2 | *Crassostrea gigas* | 0.2 |
|  | 405967637 | Tropomyosin | *Crassostrea gigas* | 0.5 |
|  | 375073719 | tropomyosin 1, partial | *Ostrea edulis* | 0.4 |
|  | 405969356 | Tudor domain-containing protein 1 | *Crassostrea gigas* | 0 |
| c89916_g1 | 762105018 | ubiquitin-conjugating enzyme E2-17 kDa-like | *Crassostrea gigas* | 0 |
| c89143_g1 | 762091586 | universal stress protein A-like protein | *Crassostrea gigas* | 0 |
| c91906_g2 | 762098971 | UPF0573 protein C2orf70 homolog A-like | *Crassostrea gigas* | 0 |
|  | 405970234 | Very long-chain specific acyl-CoA dehydrogenase, mitochondrial | *Crassostrea gigas* | 0 |
|  | 405968838 | WD repeat-containing protein 63 | *Crassostrea gigas* | 0.3 |
|  | 405970751 | WD repeat-containing protein 65 | *Crassostrea gigas* | 0.3 |
|  | 405954463 | WD repeat-containing protein C10orf79 | *Crassostrea gigas* | 0 |

Note: INF showed that protein amount in PA was 0.
