# Supplementary material for: Proteome of larval metamorphosis induced by epinephrine in the Fujian oyster Crassostrea angulata
Source: BMC Genomics. 2020 Sep 29;21:675. doi: 10.1186/s12864-020-07066-z (PMC7525975; doi:10.1186/s12864-020-07066-z)
Supplement: Supplementary file 4 — Additional file 4: Supplementary Table 3. Compared with PA, High and low level expression for differentially abundant proteins in e-SEN [file 12864_2020_7066_MOESM4_ESM.doc]

**Supplementary Table 3** Compared with PA, High and low level expression for differentially abundant proteins in e-SEN

| **Accession Number** | **NR GI** | **Identified Proteins** | **Species** | **e-SEN/PA** |
| --- | --- | --- | --- | --- |
| c99968_g1 | 762101323 | glucose-6-phosphate isomerase-like | *Crassostrea gigas* | INF |
|  | 405962781 | 26S proteasome non-ATPase regulatory subunit 3 | *Crassostrea gigas* | 2 |
| c85361_g1 | 762086880 | 4-hydroxyphenylpyruvate dioxygenase | *Crassostrea gigas* | INF |
|  | 405974071 | Actin | *Crassostrea gigas* | INF |
|  | 405969755 | Actin-3 | *Crassostrea gigas* | 6 |
| c99122_g1 | 762095292 | aldehyde dehydrogenase family 3 member B1-like | *Crassostrea gigas* | INF |
| c90043_g1 | 762146639 | alpha-amylase-like | *Crassostrea gigas* | INF |
|  | 405954380 | Alpha-soluble NSF attachment protein | *Crassostrea gigas* | INF |
|  | 405977952 | Aminopeptidase N | *Crassostrea gigas* | 2.4 |
|  | 405962570 | AP-2 complex subunit alpha-2 | *Crassostrea gigas* | 3.4 |
| c89235_g1 | 405951507 | AP-2 complex subunit mu-1 | *Crassostrea gigas* | INF |
| c90671_g1 | 762136815 | beta-catenin-like protein 1 | *Crassostrea gigas* | INF |
|  | **405969211** | **Calcium-binding mitochondrial carrier protein Aralar1** | ***Crassostrea gigas*** | **INF** |
|  | **405968450** | **Calcium-transporting ATPase sarcoplasmic/endoplasmic reticulum type** | ***Crassostrea gigas*** | **3.7** |
| **c90479_g1** | **762086942** | **calcium-transporting ATPase sarcoplasmic/endoplasmic reticulum type-like** | ***Crassostrea gigas*** | **3.9** |
| **c55559_g1** | **762161385** | **calmodulin-like** | ***Crassostrea gigas*** | **INF** |
| **c88952_g1** | **762104881** | **calumenin-like isoform X1** | ***Crassostrea gigas*** | **INF** |
|  | 405953236 | Carbonic anhydrase | *Crassostrea gigas* | INF |
| c97264_g1 | 762104782 | carbonic anhydrase 2-like | *Crassostrea gigas* | INF |
|  | 405974400 | Carbonyl reductase[NADPH 1 | *Crassostrea gigas* | 8.4 |
| c91048_g1 | 762121724 | cell migration-inducing and hyaluronan-binding protein-like | *Crassostrea gigas* | INF |
|  | 405961982 | Collagen alpha-5(VI) chain | *Crassostrea gigas* | 5.8 |
|  | 405954309 | Constitutive coactivator of PPAR-gamma-like protein 1-like protein | *Crassostrea gigas* | 3 |
|  | 229324834 | cytochrome b | *Crassostrea angulata* | INF |
|  | 187762792 | cytochrome c oxidase subunit 1 | *Crassostrea gigas* | 22 |
|  | 229324835 | cytochrome c oxidase subunit II | *Crassostrea angulata* | 2.7 |
|  | 405965494 | DnaJ-like protein subfamily B member 11 | *Crassostrea gigas* | 2.8 |
|  | 405966381 | Dynein heavy chain 6, axonemal | *Crassostrea gigas* | 4.2 |
|  | 405963852 | Dynein heavy chain 7, axonemal | *Crassostrea gigas* | 2.4 |
| c101386_g1 | 762133698 | EF-hand calcium-binding domain-containing protein 5-like isoform X1 | *Crassostrea gigas* | INF |
| c86615_g1 | 762130855 | EF-hand domain-containing family member C2-like | *Crassostrea gigas* | 2 |
| c82826_g1 | 762138085 | EF-hand domain-containing protein 1-like | *Crassostrea gigas* | 2.2 |
| c88602_g1 | 405975361 | eosinophil peroxidase-like isoform X2 | *Crassostrea gigas* | 8.4 |
| c87781_g1 | 762131945 | F-box only protein 36-like | *Crassostrea gigas* | 2 |
| c100882_g1 | 762105167 | flotillin-1-like isoform X4 | *Crassostrea gigas* | 2.4 |
| c94435_g1 | 762097616 | flotillin-2a-like | *Crassostrea gigas* | 3.5 |
| c98882_g1 | 762080824 | galactokinase-like | *Crassostrea gigas* | 2.5 |
| c97708_g1 | 762072670 | GDP-L-fucose synthase-like | *Crassostrea gigas* | INF |
|  | 56718386 | glycogen synthase | *Crassostrea gigas* | 2 |
|  | 405961245 | Heat shock 70 kDa protein 12B | *Crassostrea gigas* | 3.9 |
|  | 405962319 | Histone H3 | *Crassostrea gigas* | 2.1 |
|  | 405963114 | Hydroxysteroid dehydrogenase-like protein 2 | *Crassostrea gigas* | 6.6 |
|  | 405969882 | Importin-7 | *Crassostrea gigas* | 2.5 |
| c87788_g2 | 762102409 | integrin alpha-6-like isoform X2 | *Crassostrea gigas* | INF |
|  | 405972492 | Kinesin-related protein 1 | *Crassostrea gigas* | 4 |
| c101403_g2 | 405958866 | Lachesin | *Crassostrea gigas* | INF |
|  | 405963229 | Laminin subunit gamma-1 | *Crassostrea gigas* | 2.4 |
| **c100749_g1** | **762156704** | **MAP kinase-activated protein kinase 2-like** | ***Crassostrea gigas*** | **2.2** |
|  | 405974809 | Metabotropic glutamate receptor 3 | *Crassostrea gigas* | 5.4 |
|  | 405968797 | Methenyltetrahydrofolate synthetase domain-containing protein | *Crassostrea gigas* | 2.5 |
| c93709_g2 | 762164091 | MICOS complex subunit Mic60-like isoform X1 | *Crassostrea gigas* | 4.8 |
| c98973_g1 | 762109068 | multidrug resistance-associated protein 1-like isoform X1 | *Crassostrea gigas* | 4.9 |
|  | 405975835 | NAD(P) transhydrogenase, mitochondrial | *Crassostrea gigas* | 2 |
| c91009_g1 | 762070002 | NADPH--cytochrome P450 reductase-like | *Crassostrea gigas* | 5.5 |
|  | **405964679** | **Neurexin-4** | ***Crassostrea gigas*** | **INF** |
|  | **405960111** | **Neuroglian** | ***Crassostrea gigas*** | **INF** |
| c88012_g1 | 762111301 | Neuronal acetylcholine receptor subunit alpha-10 | *Crassostrea gigas* | 4.7 |
|  | 405966986 | Paramyosin | *Crassostrea gigas* | 3.5 |
| c94503_g2 | 762070443 | pathogen-related protein-like | *Crassostrea gigas* | INF |
|  | 405960428 | PDZ and LIM domain protein 1 | *Crassostrea gigas* | 2.2 |
|  | 405962230 | Peroxidasin | *Crassostrea gigas* | 17 |
|  | 405962229 | Peroxidasin-like protein | *Crassostrea gigas* | 3 |
|  | 405965891 | Prenylcysteine oxidase | *Crassostrea gigas* | INF |
|  | 405968717 | Programmed cell death protein 6 | *Crassostrea gigas* | 3.2 |
|  | 405965843 | Protein ERGIC-53 | *Crassostrea gigas* | 2.9 |
|  | 405965662 | Protein lap4 | *Crassostrea gigas* | INF |
|  | 405962160 | Protocadherin Fat 4 | *Crassostrea gigas* | 5.2 |
|  | **333449487** | **Ras-like GTP-binding protein RHO** | ***Crassostrea ariakensis*** | **3.8** |
| **c83633_g1** | **762141921** | **ras-related protein Rab-35-like** | ***Crassostrea gigas*** | **2.1** |
|  | **405978849** | **Rho GTPase-activating protein 17** | ***Crassostrea gigas*** | **INF** |
|  | 405962126 | rRNA 2'-O-methyltransferase fibrillarin | *Crassostrea gigas* | INF |
| **c96209_g1** | **307197748** | **Ryanodine receptor 44F** | ***Harpegnathos saltator*** | **2** |
|  | 405976087 | Splicing factor U2AF 50 kDa subunit | *Crassostrea gigas* | 2.7 |
|  | 405955028 | Steroid 17-alpha-hydroxylase/17,20 lyase | *Crassostrea gigas* | 4.1 |
|  | 405968979 | Steroid 17-alpha-hydroxylase/17,20 lyase | *Crassostrea gigas* | 2.5 |
| c85806_g1 | 762100869 | succinate dehydrogenase cytochrome b560 subunit, mitochondrial-like isoform X1 | *Crassostrea gigas* | 2.1 |
| **c78713_g1** | **821595281** | **superoxide dismutase[Mn, mitochondrial-like** | ***Crassostrea gigas*** | **8.2** |
|  | 405970435 | Thioredoxin domain-containing protein 5 | *Crassostrea gigas* | INF |
|  | 405957915 | Transmembrane protein 2 | *Crassostrea gigas* | 4.6 |
| **c83738_g1** | **762091580** | **universal stress protein A-like protein** | ***Crassostrea gigas*** | **6.1** |
| **c86426_g1** | **762094049** | **universal stress protein A-like protein** | ***Crassostrea gigas*** | **2.4** |
| **c98764_g1** | **762104468** | **14-3-3 protein zeta** | ***Crassostrea gigas*** | **0.5** |
|  | 405964277 | 26S protease regulatory subunit 4 | *Crassostrea gigas* | 0 |
|  | 405974270 | 5'-AMP-activated protein kinase subunit beta-2 | *Crassostrea gigas* | 0.3 |
| c93725_g3 | 527271971 | acyl-CoA-binding protein | *Melopsittacus undulatus* | 0 |
|  | 405962156 | Alpha-aminoadipic semialdehyde synthase, mitochondrial | *Crassostrea gigas* | 0.3 |
| c100364_g1 | 762162806 | alpha-L-fucosidase-like | *Crassostrea gigas* | 0.4 |
| c102224_g1 | 762151951 | alpha-N-acetylglucosaminidase-like isoform X2 | *Crassostrea gigas* | 0.4 |
|  | 405976514 | Amyloid protein-binding protein 2 | *Crassostrea gigas* | 0 |
|  | 405961595 | Anosmin-1 | *Crassostrea gigas* | 0 |
| **c93079_g1** | **762165574** | **apoptosis-inducing factor 3-like isoform X1** | ***Crassostrea gigas*** | **0** |
| c85397_g1 | 762138531 | ATP synthase mitochondrial F1 complex assembly factor 2-like | *Crassostrea gigas* | 0 |
|  | 405975706 | Band 4.1-like protein 3 | *Crassostrea gigas* | 0.4 |
|  | 533221120 | beta-mannosidase | *Stenotrophomonas maltophilia MF89* | 0.4 |
| c102644_g1 | 762138581 | C-1-tetrahydrofolate synthase, cytoplasmic-like | *Crassostrea gigas* | 0.4 |
| **c93407_g1** | **762076798** | **caltractin-like** | ***Crassostrea gigas*** | **0** |
|  | **405966500** | **Cathepsin L** | ***Crassostrea gigas*** | **0.3** |
| **c90692_g1** | **762167480** | **cathepsin L1-like** | ***Crassostrea gigas*** | **0.3** |
| **c88094_g1** | **762099884** | **cathepsin L1-like** | ***Crassostrea gigas*** | **0.3** |
| **c94508_g1** | **762107740** | **cathepsin L1-like** | ***Crassostrea gigas*** | **0.2** |
| c75751_g1 | 762149469 | chondroitin proteoglycan 2-like isoform X1 | *Crassostrea gigas* | 0.5 |
|  | 405959610 | Coiled-coil domain-containing protein 81 | *Crassostrea gigas* | 0.5 |
|  | 405954419 | Collagen alpha-3(VI) chain | *Crassostrea gigas* | 0.5 |
|  | 405975170 | CUB and sushi domain-containing protein 1 | *Crassostrea gigas* | 0.1 |
| c28847_g1 | 762072289 | cubilin-like | *Crassostrea gigas* | 0.2 |
| c101114_g1 | 762113070 | cystathionine beta-synthase-like isoform X6 | *Crassostrea gigas* | 0.2 |
|  | 405976185 | Deleted in malignant brain tumors 1 protein | *Crassostrea gigas* | 0.5 |
|  | 405972975 | Deleted in malignant brain tumors 1 protein | *Crassostrea gigas* | 0 |
|  | 405966631 | Deleted in malignant brain tumors 1 protein | *Crassostrea gigas* | 0 |
|  | 405975234 | Dual oxidase 2, partial | *Crassostrea gigas* | 0.3 |
| c79422_g1 | 762115410 | EF-hand calcium-binding domain-containing protein 10-like | *Crassostrea gigas* | 0 |
| c85729_g1 | 762142476 | epsin-2-like isoform X1 | *Crassostrea gigas* | 0 |
| c92449_g2 | 762129765 | ER membrane protein complex subunit 10-like isoform X1 | *Crassostrea gigas* | 0.5 |
|  | 405975002 | Eukaryotic translation initiation factor 6 | *Crassostrea gigas* | 0.5 |
| c83242_g1 | 762141095 | F-box/LRR-repeat protein 3-like | *Crassostrea gigas* | 0.5 |
|  | 405963678 | Fibropellin-1 | *Crassostrea gigas* | 0.2 |
|  | 405959171 | Glucose-6-phosphate 1-dehydrogenase | *Crassostrea gigas* | 0.4 |
|  | 405973352 | Glucose-repressible alcohol dehydrogenase transcriptional effector | *Crassostrea gigas* | 0 |
| c90196_g1 | 762099264 | glucosidase 2 subunit beta-like isoform X2 | *Crassostrea gigas* | 0.2 |
|  | 405969003 | Glutamate synthase[NADH, amyloplastic | *Crassostrea gigas* | 0.4 |
| c96570_g1 | 762134867 | glutathione reductase, mitochondrial-like | *Crassostrea gigas* | 0.4 |
| c98962_g1 | 762106909 | glycogenin-1-like isoform X3 | *Crassostrea gigas* | 0.5 |
|  | 405963608 | Heat shock 70 kDa protein 12B | *Crassostrea gigas* | 0.3 |
| **c82792_g1** | **762131241** | **heat shock protein 27-like** | ***Crassostrea gigas*** | **0.3** |
|  | 405952109 | Hemicentin-1 | *Crassostrea gigas* | 0.5 |
|  | 405969689 | Hydrocephalus-inducing-like protein | *Crassostrea gigas* | 0.2 |
|  | 405950357 | Integrin alpha-6 | *Crassostrea gigas* | 0.3 |
|  | **405950471** | **Integrin alpha-8** | ***Crassostrea gigas*** | **0** |
| **c100530_g1** | **762106758** | **integrin beta pat-3-like** | ***Crassostrea gigas*** | **0** |
|  | 405968311 | Isocitrate dehydrogenase[NAD subunit alpha, mitochondrial | *Crassostrea gigas* | 0.5 |
|  | 405977054 | Kinesin heavy chain | *Crassostrea gigas* | 0.4 |
| **c86938_g1** | **405953294** | **Kyphoscoliosis peptidase** | ***Crassostrea gigas*** | **0.5** |
| c103140_g1 | 762101975 | laccase-4-like | *Crassostrea gigas* | 0 |
|  | 405952168 | La-related protein 4 | *Crassostrea gigas* | 0 |
|  | 405962612 | Leucine-rich repeats and immunoglobulin-like domains protein 3 | *Crassostrea gigas* | 0.5 |
| c99032_g1 | 405971834 | Leukocyte elastase inhibitor | *Crassostrea gigas* | 0.5 |
|  | 405967541 | LIM and SH3 domain protein Lasp | *Crassostrea gigas* | 0.5 |
|  | 405952731 | Lupus La-like protein | *Crassostrea gigas* | 0.1 |
| **c99479_g1** | **762118717** | **lysosome-associated membrane glycoprotein 1-like isoform X2** | ***Crassostrea gigas*** | **0.5** |
|  | 405965903 | Major egg antigen | *Crassostrea gigas* | 0 |
|  | 405965621 | Mammalian ependymin-related protein 1, partial | *Crassostrea gigas* | 0.5 |
| c95118_g1 | 762168992 | minus strand |  | 0.1 |
| c78564_g1 | 405973457 | minus strand |  | 0.09 |
| c93185_g1 | 405950468 | minus strand |  | 0 |
|  | 405960381 | Monocarboxylate transporter 12 | *Crassostrea gigas* | 0.4 |
| **c134536_g1** | **762077014** | **mucin-2-like** | ***Crassostrea gigas*** | **0** |
| **c7692_g1** | **871278596** | **mucin-5AC-like** | ***Aplysia californica*** | **0.2** |
| **c102473_g1** | **762076941** | **mucin-5AC-like** | ***Crassostrea gigas*** | **0** |
| **c95895_g1** | **762099838** | **mucin-5AC-like** | ***Crassostrea gigas*** | **0** |
| **c91752_g1** | **762100460** | **mucin-like protein** | ***Crassostrea gigas*** | **0.04** |
|  | 405970698 | Multidrug resistance protein 1 | *Crassostrea gigas* | 0 |
|  | 405975739 | Murinoglobulin-2 | *Crassostrea gigas* | 0.3 |
|  | 405967527 | Nesprin-1 | *Crassostrea gigas* | 0.06 |
| **c101658_g2** | **762095530** | **neural-cadherin-like** | ***Crassostrea gigas*** | **0** |
|  | **405963373** | **Neurogenic locus Notch protein** | ***Crassostrea gigas*** | **0** |
|  | **405958312** | **Neuroglian** | ***Crassostrea gigas*** | **0.4** |
| **c96718_g1** | **762143197** | **neutral ceramidase-like** | ***Crassostrea gigas*** | **0** |
|  | **405952329** | **PAB-dependent poly(A)-specific ribonuclease subunit 2** | ***Crassostrea gigas*** | **0.5** |
|  | **405977917** | **Peroxisomal multifunctional enzyme type 2** | ***Crassostrea gigas*** | **0** |
| **c93516_g1** | **762084138** | **peroxisomal multifunctional enzyme type 2-like** | ***Crassostrea gigas*** | **0** |
| **c87836_g1** | **762127059** | **Peroxisomal NADH pyrophosphatase NUDT12** | ***Crassostrea gigas*** | **0.4** |
| c93257_g1 | 762136319 | phosphomannomutase-like isoform X1 | *Crassostrea gigas* | 0.5 |
|  | 405975722 | Polyamine-modulated factor 1-binding protein 1 | *Crassostrea gigas* | 0 |
| c101643_g2 | 762115946 | pregnancy zone protein-like | *Crassostrea gigas* | 0.5 |
|  | 405960660 | Protein disulfide-isomerase A5 | *Crassostrea gigas* | 0.3 |
| c91045_g2 | 762163372 | protein disulfide-isomerase A5-like | *Crassostrea gigas* | 0.5 |
|  | 405962525 | Protein FAM63B | *Crassostrea gigas* | 0 |
|  | 405969398 | Protein jagged-2 | *Crassostrea gigas* | 0 |
|  | 405975243 | Protein unc-87 | *Crassostrea gigas* | 0.5 |
|  | 405963677 | Protein VPRBP | *Crassostrea gigas* | 0.5 |
|  | 405950809 | Protocadherin Fat 4 | *Crassostrea gigas* | 0.5 |
| c48957_g1 | 405951929 | protocadherin Fat 4-like | *Crassostrea gigas* | 0.3 |
| c101457_g1 | 762080804 | protocadherin Fat 4-like isoform X3 | *Crassostrea gigas* | 0.5 |
|  | 405951930 | Protocadherin-like wing polarity protein stan | *Crassostrea gigas* | 0 |
|  | 405974135 | Putative chitinase 3 | *Crassostrea gigas* | 0.2 |
| c96793_g1 | 405970391 | putative per-hexamer repeat protein 5 isoform X9 | *Crassostrea gigas* | 0.4 |
|  | 405957461 | Putative thiopurine S-methyltransferase | *Crassostrea gigas* | 0.2 |
| **c70729_g1** | **762119151** | **radial spoke head protein 4 homolog A-like** | ***Crassostrea gigas*** | **0.5** |
|  | **405976660** | **Ran GTPase-activating protein 1** | ***Crassostrea gigas*** | **0** |
|  | 405970353 | Replication protein A 70 kDa DNA-binding subunit | *Crassostrea gigas* | 0.1 |
|  | 405953430 | Ribosome-binding protein 1 | *Crassostrea gigas* | 0.3 |
| c103590_g1 | 762100293 | rootletin-like isoform X6 | *Crassostrea gigas* | 0.4 |
|  | 405967048 | Scaffold attachment factor B1 | *Crassostrea gigas* | 0 |
| c100128_g1 | 762155471 | scavenger receptor cysteine-rich type 1 protein M130-like | *Crassostrea gigas* | 0 |
| **c86391_g1** | **405973590** | **SCO-spondin** | ***Crassostrea gigas*** | **0.5** |
|  | **405966926** | **SCO-spondin** | ***Crassostrea gigas*** | **0.2** |
|  | 405972994 | SEC13-like protein | *Crassostrea gigas* | 0.2 |
|  | 405973087 | SH3 domain-binding glutamic acid-rich protein | *Crassostrea gigas* | 0 |
| **c86843_g1** | **762100962** | **soma ferritin-like** | ***Crassostrea gigas*** | **0.5** |
|  | 405965139 | Sorting nexin-2 | *Crassostrea gigas* | 0.4 |
| c99017_g1 | 762122989 | spectrin alpha chain-like isoform X6 | *Crassostrea gigas* | 0.2 |
| c94466_g1 | 762104107 | stress-induced-phosphoprotein 1-like | *Crassostrea gigas* | 0.5 |
| c80732_g1 | 762169708 | tektin-2-like | *Crassostrea gigas* | 0.4 |
|  | 405960104 | Tenascin-X | *Crassostrea gigas* | 0.2 |
|  | 405972180 | Tetratricopeptide repeat protein 25 | *Crassostrea gigas* | 0 |
|  | 405952729 | Titin | *Crassostrea gigas* | 0.4 |
|  | 405970417 | Titin | *Crassostrea gigas* | 0.3 |
|  | 219806594 | tropomyosin | *Crassostrea gigas* | 0.5 |
|  | 405967637 | Tropomyosin | *Crassostrea gigas* | 0.4 |
|  | 375073719 | tropomyosin 1, partial | *Ostrea edulis* | 0.3 |
| c86784_g5 | 871236028 | troponin I-like | *Aplysia californica* | 0.5 |
|  | 405969356 | Tudor domain-containing protein 1 | *Crassostrea gigas* | 0 |
|  | 405976987 | Tyrosine-protein phosphatase Lar | *Crassostrea gigas* | 0 |
|  | 405957445 | Tyrosine-protein phosphatase non-receptor type 6 | *Crassostrea gigas* | 0 |
| c96730_g1 | 762108713 | ubiquilin-1-like | *Crassostrea gigas* | 0.4 |
| c102381_g1 | 762129367 | ubiquitin carboxyl-terminal hydrolase 15-like | *Crassostrea gigas* | 0.4 |
|  | 405969345 | Ubiquitin-associated protein 2 | *Crassostrea gigas* | 0.1 |
| c77339_g1 | 762140796 | ubiquitin-conjugating enzyme E2 variant 2-like isoform X1 | *Crassostrea gigas* | 0.2 |
| c89916_g1 | 762105018 | ubiquitin-conjugating enzyme E2-17 kDa-like | *Crassostrea gigas* | 0 |
| c91906_g2 | 762098971 | UPF0573 protein C2orf70 homolog A-like | *Crassostrea gigas* | 0 |
|  | 405963809 | Vacuolar protein sorting-associated protein 4B | *Crassostrea gigas* | 0.5 |
|  | 405970234 | Very long-chain specific acyl-CoA dehydrogenase, mitochondrial | *Crassostrea gigas* | 0 |
| **c102546_g1** | **762087607** | **vinculin-like isoform X7** | ***Crassostrea gigas*** | **0** |
|  | 405960135 | Voltage-dependent calcium channel subunit alpha-2/delta-2 | *Crassostrea gigas* | 0.2 |
|  | 405969928 | von Willebrand factor D and EGF domain-containing protein | *Crassostrea gigas* | 0.09 |
|  | 405972713 | von Willebrand factor D and EGF domain-containing protein | *Crassostrea gigas* | 0.07 |
| **c96390_g1** | **762145704** | **V-type proton ATPase subunit S1-like** | ***Crassostrea gigas*** | **0.2** |
|  | 405954463 | WD repeat-containing protein C10orf79 | *Crassostrea gigas* | 0.3 |

Note: INF showed that protein amount in PA was 0.
