# Supplementary material for: Proteome of larval metamorphosis induced by epinephrine in the Fujian oyster Crassostrea angulata
Source: BMC Genomics. 2020 Sep 29;21:675. doi: 10.1186/s12864-020-07066-z (PMC7525975; doi:10.1186/s12864-020-07066-z)
Supplement: Supplementary file 5 — Additional file 5: Supplementary Table 4. Compared with MET, High and low level expression for differentially abundant proteins in eMET [file 12864_2020_7066_MOESM5_ESM.doc]

**Supplementary Table 4** Compared with MET, High and low level expression for differentially abundant proteins in eMET

| **Accession Number** | **NR GI** | **Identified Proteins** | **Species** | **e-MET/MET** |
| --- | --- | --- | --- | --- |
| **c98764_g1** | **762104468** | **14-3-3 protein zeta** | ***Crassostrea gigas*** | **3** |
|  | 405966858 | 3-hydroxyacyl-CoA dehydrogenase type-2 | *Crassostrea gigas* | 2 |
|  | 405960426 | 4-aminobutyrate aminotransferase, mitochondrial | *Crassostrea gigas* | 2.7 |
|  | 405965820 | 60S ribosomal protein L26 | *Crassostrea gigas* | INF |
|  | 405965901 | 60S ribosomal protein L27a | *Crassostrea gigas* | INF |
|  | 405959119 | 60S ribosomal protein L3, partial | *Crassostrea gigas* | 3.2 |
|  | 405977927 | 60S ribosomal protein L9 | *Crassostrea gigas* | 2.2 |
|  | 405968537 | Actin | *Crassostrea gigas* | 7.5 |
| c93725_g3 | 527271971 | acyl-CoA-binding protein | *Melopsittacus undulatus* | INF |
|  | 405972978 | Adenylosuccinate synthetase | *Crassostrea gigas* | INF |
| c101293_g1 | 762096787 | alanine aminotransferase 1-like | *Crassostrea gigas* | INF |
| c99122_g1 | 762095292 | aldehyde dehydrogenase family 3 member B1-like | *Crassostrea gigas* | INF |
| c90043_g1 | 762146639 | alpha-amylase-like | *Crassostrea gigas* | INF |
|  | 405976514 | Amyloid protein-binding protein 2 | *Crassostrea gigas* | INF |
|  | 405962570 | AP-2 complex subunit alpha-2 | *Crassostrea gigas* | 2 |
| c89235_g1 | 405951507 | AP-2 complex subunit mu-1 | *Crassostrea gigas* | INF |
| **c93079_g1** | **762165574** | **apoptosis-inducing factor 3-like isoform X1** | ***Crassostrea gigas*** | **4** |
| c85397_g1 | 762138531 | ATP synthase mitochondrial F1 complex assembly factor 2-like | *Crassostrea gigas* | 6.4 |
|  | 405969358 | ATPase family AAA domain-containing protein 2B | *Crassostrea gigas* | 2.1 |
| c103615_g1 | 762111269 | ATP-citrate synthase-like isoform X1 | *Crassostrea gigas* | INF |
|  | 405975706 | Band 4.1-like protein 3 | *Crassostrea gigas* | 5.6 |
| c90671_g1 | 762136815 | beta-catenin-like protein 1 | *Crassostrea gigas* | INF |
|  | **405964165** | **Calcium/calmodulin-dependent protein kinase type II delta chain** | ***Crassostrea gigas*** | **INF** |
|  | **405972360** | **Calpain-7-like protein** | ***Crassostrea gigas*** | **INF** |
| c97264_g1 | 762104782 | carbonic anhydrase 2-like | *Crassostrea gigas* | INF |
|  | **405959835** | **Catalase** | ***Crassostrea gigas*** | **2.6** |
| **c91059_g3** | **762099416** | **chitinase-3-like protein 1 isoform X3** | ***Crassostrea gigas*** | **3.6** |
| c75751_g1 | 762149469 | chondroitin proteoglycan 2-like isoform X1 | *Crassostrea gigas* | 3.6 |
| **c99303_g1** | **762156177** | **cilia- and flagella-associated protein 61-like** | ***Crassostrea gigas*** | **INF** |
| c82901_g1 | 762091603 | citrate synthase, mitochondrial-like isoform X1 | *Crassostrea gigas* | 2.1 |
|  | 405959610 | Coiled-coil domain-containing protein 81 | *Crassostrea gigas* | INF |
| c93420_g1 | 762126464 | cold shock domain-containing protein 3-like | *Crassostrea gigas* | 2 |
|  | 405975170 | CUB and sushi domain-containing protein 1 | *Crassostrea gigas* | 2 |
|  | 229324834 | cytochrome b | *Crassostrea angulata* | INF |
|  | 405972975 | Deleted in malignant brain tumors 1 protein | *Crassostrea gigas* | 8.5 |
|  | 405953044 | Dynein beta chain, ciliary | *Crassostrea gigas* | 3.7 |
|  | 405977373 | Dynein heavy chain 1, axonemal | *Crassostrea gigas* | INF |
|  | 405950869 | Dynein heavy chain 3, axonemal | *Crassostrea gigas* | 9.8 |
|  | 405971451 | Dynein heavy chain 5, axonemal | *Crassostrea gigas* | INF |
|  | 405966381 | Dynein heavy chain 6, axonemal | *Crassostrea gigas* | INF |
|  | 405969117 | Dynein heavy chain 7, axonemal | *Crassostrea gigas* | INF |
|  | 405963852 | Dynein heavy chain 7, axonemal | *Crassostrea gigas* | INF |
|  | 405969825 | E3 ubiquitin-protein ligase HUWE1 | *Crassostrea gigas* | 8.8 |
| **c79422_g1** | **762115410** | **EF-hand calcium-binding domain-containing protein 10-like** | ***Crassostrea gigas*** | **INF** |
| **c101386_g1** | **762133698** | **EF-hand calcium-binding domain-containing protein 5-like isoform X1** | ***Crassostrea gigas*** | **INF** |
|  | **405963739** | **EF-hand calcium-binding domain-containing protein 6** | ***Crassostrea gigas*** | **INF** |
| c86615_g1 | 762130855 | EF-hand domain-containing family member C2-like | *Crassostrea gigas* | 6.3 |
| c82826_g1 | 762138085 | EF-hand domain-containing protein 1-like | *Crassostrea gigas* | 2 |
| c88602_g1 | 405975361 | eosinophil peroxidase-like isoform X2 | *Crassostrea gigas* | INF |
| c85729_g1 | 762142476 | epsin-2-like isoform X1 | *Crassostrea gigas* | 3.6 |
| c92454_g1 | 762156349 | F-box only protein 21-like | *Crassostrea gigas* | 4.6 |
| c87781_g1 | 762131945 | F-box only protein 36-like | *Crassostrea gigas* | 3.9 |
| c83242_g1 | 762141095 | F-box/LRR-repeat protein 3-like | *Crassostrea gigas* | 3.1 |
| c97708_g1 | 762072670 | GDP-L-fucose synthase-like | *Crassostrea gigas* | INF |
|  | 405959171 | Glucose-6-phosphate 1-dehydrogenase | *Crassostrea gigas* | INF |
| c99968_g1 | 762101323 | glucose-6-phosphate isomerase-like | *Crassostrea gigas* | INF |
| c90196_g1 | 762099264 | glucosidase 2 subunit beta-like isoform X2 | *Crassostrea gigas* | 3.1 |
| c102113_g1 | 762136488 | glycerol-3-phosphate dehydrogenase, mitochondrial-like isoform X2 | *Crassostrea gigas* | INF |
|  | 405952109 | Hemicentin-1 | *Crassostrea gigas* | 4.9 |
|  | 405969689 | Hydrocephalus-inducing-like protein | *Crassostrea gigas* | INF |
|  | 405950357 | Integrin alpha-6 | *Crassostrea gigas* | 2.2 |
|  | 405950471 | Integrin alpha-8 | *Crassostrea gigas* | 2.3 |
|  | 405963851 | IQ and ubiquitin-like domain-containing protein | *Crassostrea gigas* | INF |
|  | 405973588 | Kielin/chordin-like protein | *Crassostrea gigas* | 2.4 |
|  | 405967541 | LIM and SH3 domain protein Lasp | *Crassostrea gigas* | 2.2 |
| c95819_g1 | 405977265 | Long-chain specific acyl-CoA dehydrogenase, mitochondrial | *Crassostrea gigas* | 3.1 |
|  | 405972616 | Methylcrotonoyl-CoA carboxylase subunit alpha, mitochondrial | *Crassostrea gigas* | 3.5 |
| c95118_g1 | 762168992 | minus strand |  | 5.7 |
| **c97912_g1** | **762107124** | **mitogen-activated protein kinase 1-like** | ***Crassostrea gigas*** | **INF** |
|  | 405960381 | Monocarboxylate transporter 12 | *Crassostrea gigas* | 2.4 |
| **c86391_g2** | **762085926** | **mucin-17-like** | ***Crassostrea gigas*** | **2.3** |
| **c11357_g1** | **762080102** | **mucin-19-like** | ***Crassostrea gigas*** | **INF** |
| **c98108_g1** | **762085928** | **mucin-5AC-like** | ***Crassostrea gigas*** | **2.6** |
| **c91752_g1** | **762100460** | **mucin-like protein** | ***Crassostrea gigas*** | **INF** |
|  | 405965726 | NADP-dependent malic enzyme | *Crassostrea gigas* | 2.5 |
| c91009_g1 | 762070002 | NADPH--cytochrome P450 reductase-like | *Crassostrea gigas* | INF |
| **c101658_g2** | **762095530** | **neural-cadherin-like** | ***Crassostrea gigas*** | **2.1** |
|  | **405963373** | **Neurogenic locus Notch protein** | ***Crassostrea gigas*** | **2.9** |
| c88012_g1 | 762111301 | Neuronal acetylcholine receptor subunit alpha-10 | *Crassostrea gigas* | INF |
|  | 405951454 | Outer dense fiber protein 3 | *Crassostrea gigas* | 25 |
|  | 405952329 | PAB-dependent poly(A)-specific ribonuclease subunit 2 | *Crassostrea gigas* | 4 |
|  | 405960428 | PDZ and LIM domain protein 1 | *Crassostrea gigas* | 2 |
|  | 405962230 | Peroxidasin | *Crassostrea gigas* | INF |
|  | 405962229 | Peroxidasin-like protein | *Crassostrea gigas* | INF |
|  | **405974897** | **Peroxiredoxin-5, mitochondrial** | ***Crassostrea gigas*** | **2** |
|  | **405977917** | **Peroxisomal multifunctional enzyme type 2** | ***Crassostrea gigas*** | **INF** |
| **c93516_g1** | **762084138** | **peroxisomal multifunctional enzyme type 2-like** | ***Crassostrea gigas*** | **INF** |
| **c87836_g1** | **762127059** | **Peroxisomal NADH pyrophosphatase NUDT12** | ***Crassostrea gigas*** | **2** |
|  | 405950592 | Phosphoglucomutase-1 | *Crassostrea gigas* | 2.8 |
|  | 405958107 | Poly[ADP-ribose polymerase 1 | *Crassostrea gigas* | INF |
|  | 405975722 | Polyamine-modulated factor 1-binding protein 1 | *Crassostrea gigas* | 22 |
|  | 405972892 | Protein SPATIAL | *Crassostrea gigas* | 11 |
|  | 405951163 | Putative adenylate kinase-like protein C9orf98-like protein | *Crassostrea gigas* | INF |
| c95972_g1 | 762095264 | putative malate dehydrogenase 1B | *Crassostrea gigas* | INF |
| c96793_g1 | 405970391 | putative per-hexamer repeat protein 5 isoform X9 | *Crassostrea gigas* | 2.8 |
| **c75090_g1** | **762085934** | **radial spoke head 1 homolog** | ***Crassostrea gigas*** | **11** |
| **c91646_g1** | **762084810** | **radial spoke head protein 3 homolog B-like** | ***Crassostrea gigas*** | **2** |
| **c70729_g1** | **762119151** | **radial spoke head protein 4 homolog A-like** | ***Crassostrea gigas*** | **2.6** |
|  | 405972837 | Retinal dehydrogenase 1 | *Crassostrea gigas* | 2.9 |
|  | 405953430 | Ribosome-binding protein 1 | *Crassostrea gigas* | 2.3 |
| c103590_g1 | 762100293 | rootletin-like isoform X6 | *Crassostrea gigas* | 6.8 |
|  | 405962126 | rRNA 2'-O-methyltransferase fibrillarin | *Crassostrea gigas* | INF |
| c96209_g1 | 307197748 | Ryanodine receptor 44F | *Harpegnathos saltator* | INF |
|  | 405967048 | Scaffold attachment factor B1 | *Crassostrea gigas* | INF |
| **c86391_g1** | **405973590** | **SCO-spondin** | ***Crassostrea gigas*** | **4.8** |
|  | **405973589** | **SCO-spondin** | ***Crassostrea gigas*** | **2.5** |
| **c83004_g1** | **675373238** | **SCO-spondin, partial** | ***Stegodyphus mimosarum*** | **2.7** |
|  | 405964168 | Serine/threonine-protein phosphatase 2B catalytic subunit alpha isoform | *Crassostrea gigas* | 4.2 |
| **c86843_g1** | **762100962** | **soma ferritin-like** | ***Crassostrea gigas*** | **18** |
| c99017_g1 | 762122989 | spectrin alpha chain-like isoform X6 | *Crassostrea gigas* | 2 |
|  | 405961240 | Sperm surface protein Sp17 | *Crassostrea gigas* | 2.3 |
|  | 405968979 | Steroid 17-alpha-hydroxylase/17,20 lyase | *Crassostrea gigas* | INF |
| c85806_g1 | 762100869 | succinate dehydrogenase cytochrome b560 subunit, mitochondrial-like isoform X1 | *Crassostrea gigas* | INF |
| **c78713_g1** | **821595281** | **superoxide dismutase[Mn, mitochondrial-like** | ***Crassostrea gigas*** | **INF** |
| c99665_g2 | 405965400 | Synaptopodin-2 | *Crassostrea gigas* | 3.4 |
|  | 405953549 | T-complex protein 1 subunit zeta | *Crassostrea gigas* | 3.5 |
|  | 405952896 | Tectonic-3 | *Crassostrea gigas* | 2.8 |
| c80732_g1 | 762169708 | tektin-2-like | *Crassostrea gigas* | 7.3 |
|  | 405975469 | Tektin-3 | *Crassostrea gigas* | 7.2 |
| c89247_g1 | 762135468 | tektin-3-like isoform X2 | *Crassostrea gigas* | 4.5 |
|  | 405972180 | Tetratricopeptide repeat protein 25 | *Crassostrea gigas* | 5.4 |
|  | 405970417 | Titin | *Crassostrea gigas* | 2 |
|  | 405966231 | Transmembrane protein 2 | *Crassostrea gigas* | 2.4 |
|  | 405967637 | Tropomyosin | *Crassostrea gigas* | 6.5 |
|  | 219806594 | tropomyosin | *Crassostrea gigas* | 2.6 |
|  | 375073719 | tropomyosin 1, partial | *Ostrea edulis* | 3.5 |
|  | 405965308 | Troponin T, skeletal muscle | *Crassostrea gigas* | 2.8 |
|  | 405969356 | Tudor domain-containing protein 1 | *Crassostrea gigas* | INF |
|  | 405957445 | Tyrosine-protein phosphatase non-receptor type 6 | *Crassostrea gigas* | 3.1 |
| c89916_g1 | 762105018 | ubiquitin-conjugating enzyme E2-17 kDa-like | *Crassostrea gigas* | INF |
| **c89143_g1** | **762091586** | **universal stress protein A-like protein** | ***Crassostrea gigas*** | **INF** |
| c91906_g2 | 762098971 | UPF0573 protein C2orf70 homolog A-like | *Crassostrea gigas* | INF |
|  | 405970234 | Very long-chain specific acyl-CoA dehydrogenase, mitochondrial | *Crassostrea gigas* | INF |
|  | 405960135 | Voltage-dependent calcium channel subunit alpha-2/delta-2 | *Crassostrea gigas* | 3.5 |
|  | 405968838 | WD repeat-containing protein 63 | *Crassostrea gigas* | 3.2 |
|  | 405970751 | WD repeat-containing protein 65 | *Crassostrea gigas* | 2.1 |
|  | 405954463 | WD repeat-containing protein C10orf79 | *Crassostrea gigas* | INF |
|  | 405964277 | 26S protease regulatory subunit 4 | *Crassostrea gigas* | 0.5 |
|  | 405957859 | 26S protease regulatory subunit 6A | *Crassostrea gigas* | 0.5 |
|  | 405962781 | 26S proteasome non-ATPase regulatory subunit 3 | *Crassostrea gigas* | 0.2 |
|  | 405955617 | 3-hydroxyanthranilate 3,4-dioxygenase | *Crassostrea gigas* | 0.3 |
|  | 405963175 | 60 kDa neurofilament protein | *Crassostrea gigas* | 0.5 |
|  | 405958039 | Acidic leucine-rich nuclear phosphoprotein 32 family member A | *Crassostrea gigas* | 0.5 |
|  | 405974071 | Actin | *Crassostrea gigas* | 0.5 |
|  | 405961891 | Alpha-crystallin B chain | *Crassostrea gigas* | 0.5 |
| c100364_g1 | 762162806 | alpha-L-fucosidase-like | *Crassostrea gigas* | 0.4 |
| c92241_g2 | 762101727 | alpha-L-fucosidase-like isoform X2 | *Crassostrea gigas* | 0.5 |
|  | 405970312 | Alpha-N-acetylgalactosaminidase | *Crassostrea gigas* | 0.5 |
|  | 405954380 | Alpha-soluble NSF attachment protein | *Crassostrea gigas* | 0 |
|  | 405977952 | Aminopeptidase N | *Crassostrea gigas* | 0 |
| c80803_g1 | 405970526 | Annexin A7, partial | *Crassostrea gigas* | 0 |
| c88705_g1 | 762167760 | ATP synthase F(0) complex subunit B1, mitochondrial-like | *Crassostrea gigas* | 0.5 |
|  | 405965163 | Beta-hexosaminidase subunit beta | *Crassostrea gigas* | 0.4 |
|  | 533221120 | beta-mannosidase | *Stenotrophomonas maltophilia MF89* | 0 |
|  | 405967658 | Bifunctional aminoacyl-tRNA synthetase | *Crassostrea gigas* | 0 |
| **c100614_g1** | **762091278** | **calcium uniporter protein, mitochondrial-like** | ***Crassostrea gigas*** | **0** |
|  | **405969211** | **Calcium-binding mitochondrial carrier protein Aralar1** | ***Crassostrea gigas*** | **0.3** |
|  | **405968450** | **Calcium-transporting ATPase sarcoplasmic/endoplasmic reticulum type** | ***Crassostrea gigas*** | **0.3** |
| **c90479_g1** | **762086942** | **calcium-transporting ATPase sarcoplasmic/endoplasmic reticulum type-like** | ***Crassostrea gigas*** | **0.4** |
|  | **20137620** | **Calmodulin; Short=CaM** |  | **0.5** |
| **c55559_g1** | **762161385** | **calmodulin-like** | ***Crassostrea gigas*** | **0** |
| **c97263_g1** | **405967580** | **Calnexin** | ***Crassostrea gigas*** | **0.2** |
| **c98322_g1** | **762079826** | **calumenin-like isoform X1** | ***Crassostrea gigas*** | **0.2** |
|  | 405953236 | Carbonic anhydrase | *Crassostrea gigas* | 0.09 |
| **c90692_g1** | **762167480** | **cathepsin L1-like** | ***Crassostrea gigas*** | **0.5** |
| **c102687_g1** | **762092188** | **CD109 antigen-like** | ***Crassostrea gigas*** | **0.3** |
|  | 405963691 | Cofilin | *Crassostrea gigas* | 0.3 |
|  | **405954419** | **Collagen alpha-3(VI) chain** | ***Crassostrea gigas*** | **0.2** |
|  | **405961982** | **Collagen alpha-5(VI) chain** | ***Crassostrea gigas*** | **0** |
|  | 405974697 | C-terminal-binding protein | *Crassostrea gigas* | 0.3 |
| c101114_g1 | 762113070 | cystathionine beta-synthase-like isoform X6 | *Crassostrea gigas* | 0 |
| c77693_g1 | 765826145 | cytochrome b-c1 complex subunit Rieske, mitochondrial-like | *Crassostrea gigas* | 0 |
|  | 229324835 | cytochrome c oxidase subunit II | *Crassostrea angulata* | 0.2 |
|  | 405966262 | Deleted in malignant brain tumors 1 protein | *Crassostrea gigas* | 0 |
|  | 405965494 | DnaJ-like protein subfamily B member 11 | *Crassostrea gigas* | 0.09 |
| **c90254_g1** | **762155518** | **drebrin-like protein B isoform X2** | ***Crassostrea gigas*** | **0.5** |
|  | 405975234 | Dual oxidase 2, partial | *Crassostrea gigas* | 0.3 |
|  | 405968675 | EF-hand domain-containing protein D1 | *Crassostrea gigas* | 0.2 |
|  | 405971816 | Endoplasmic reticulum aminopeptidase 1 | *Crassostrea gigas* | 0 |
|  | 405970058 | Eukaryotic translation initiation factor 3 subunit A | *Crassostrea gigas* | 0 |
|  | 405951422 | Eukaryotic translation initiation factor 3 subunit G-A | *Crassostrea gigas* | 0.2 |
|  | 405968987 | Fatty acid-binding-like protein 5 | *Crassostrea gigas* | 0.3 |
| c100882_g1 | 762105167 | flotillin-1-like isoform X4 | *Crassostrea gigas* | 0.1 |
| c94435_g1 | 762097616 | flotillin-2a-like | *Crassostrea gigas* | 0.2 |
| c98882_g1 | 762080824 | galactokinase-like | *Crassostrea gigas* | 0.06 |
| c100029_g2 | 762099370 | gastric intrinsic factor-like | *Crassostrea gigas* | 0.5 |
| c82585_g2 | 762145041 | glia maturation factor beta-like | *Crassostrea gigas* | 0.07 |
|  | 405973024 | Glutaredoxin-3 | *Crassostrea gigas* | 0.5 |
| c98484_g1 | 405964519 | glycerol-3-phosphate dehydrogenase[NAD(+), cytoplasmic-like | *Crassostrea gigas* | 0.4 |
|  | 56718386 | glycogen synthase | *Crassostrea gigas* | 0 |
| **c101959_g1** | **405975684** | **HEAT repeat-containing protein 7A** | ***Crassostrea gigas*** | **0.2** |
|  | **405963608** | **Heat shock 70 kDa protein 12B** | ***Crassostrea gigas*** | **0** |
| **c89955_g1** | **762129389** | **heat shock 70 kDa protein 14-like** | ***Crassostrea gigas*** | **0.3** |
| **c82792_g1** | **762131241** | **heat shock protein 27-like** | ***Crassostrea gigas*** | **0.3** |
|  | 405952153 | Heterogeneous nuclear ribonucleoprotein H | *Crassostrea gigas* | 0.4 |
|  | 405957027 | Heterogeneous nuclear ribonucleoprotein K | *Crassostrea gigas* | 0.4 |
|  | 405959264 | Heterogeneous nuclear ribonucleoprotein U-like protein 1 | *Crassostrea gigas* | 0.4 |
|  | 405978261 | Hexokinase type 2 | *Crassostrea gigas* | 0.1 |
|  | 405962051 | Importin subunit alpha-3 | *Crassostrea gigas* | 0.3 |
|  | 405973144 | Importin subunit beta-1 | *Crassostrea gigas* | 0.2 |
|  | 405958012 | Importin-5 | *Crassostrea gigas* | 0.3 |
|  | 405969882 | Importin-7 | *Crassostrea gigas* | 0 |
| c87788_g2 | 762102409 | integrin alpha-6-like isoform X2 | *Crassostrea gigas* | 0 |
| c101066_g1 | 762162532 | integrin beta-1-B-like | *Crassostrea gigas* | 0.5 |
| c95812_g1 | 762147513 | interferon-induced protein 44-like | *Crassostrea gigas* | 0.3 |
| c96333_g1 | 762095272 | isocitrate dehydrogenase [NADP cytoplasmic-like | *Crassostrea gigas* | 0.4 |
|  | 405972492 | Kinesin-related protein 1 | *Crassostrea gigas* | 0 |
| c101403_g2 | 405958866 | Lachesin | *Crassostrea gigas* | 0 |
|  | **405969732** | **Laminin subunit alpha** | ***Crassostrea gigas*** | **0.09** |
| **c103776_g1** | **762109423** | **laminin subunit alpha-like** | ***Crassostrea gigas*** | **0.1** |
|  | **405963229** | **Laminin subunit gamma-1** | ***Crassostrea gigas*** | **0** |
|  | 405950801 | La-related protein 1 | *Crassostrea gigas* | 0.2 |
|  | 405952168 | La-related protein 4 | *Crassostrea gigas* | 0.5 |
| **c99318_g1** | **762141840** | **lethal(2) giant larvae protein homolog 2-like isoform X1** | ***Crassostrea gigas*** | **0.3** |
|  | **405952027** | **Lethal(2) giant larvae-like protein 1** | ***Crassostrea gigas*** | **0.4** |
| c96717_g1 | 762130693 | leucine-rich repeat and death domain-containing protein 1-like | *Crassostrea gigas* | 0.5 |
|  | 405952986 | Leucine-rich repeat-containing G-protein coupled receptor 6 | *Crassostrea gigas* | 0.3 |
|  | 405962612 | Leucine-rich repeats and immunoglobulin-like domains protein 3 | *Crassostrea gigas* | 0.4 |
|  | 405952151 | Long-chain-fatty-acid--CoA ligase 1 | *Crassostrea gigas* | 0.2 |
| c86491_g1 | 762157957 | mechanosensory protein 2-like isoform X3 | *Crassostrea gigas* | 0.3 |
|  | 405959230 | Membrane metallo-endopeptidase-like 1 | *Crassostrea gigas* | 0.1 |
|  | 405974809 | Metabotropic glutamate receptor 3 | *Crassostrea gigas* | 0.009 |
|  | 405968797 | Methenyltetrahydrofolate synthetase domain-containing protein | *Crassostrea gigas* | 0.09 |
| c93185_g1 | 405950468 | minus strand |  | 0.5 |
| c85400_g1 | 406817026 | minus strand |  | 0.4 |
| c95340_g1 | 762099550 | minus strand |  | 0.4 |
| c98275_g1 | 665815290 | minus strand |  | 0.3 |
| c78564_g1 | 405973457 | minus strand |  | 0 |
|  | 405970698 | Multidrug resistance protein 1 | *Crassostrea gigas* | 0 |
|  | 405956360 | Multidrug resistance protein 1, partial | *Crassostrea gigas* | 0.02 |
| c98973_g1 | 762109068 | multidrug resistance-associated protein 1-like isoform X1 | *Crassostrea gigas* | 0 |
|  | 405975835 | NAD(P) transhydrogenase, mitochondrial | *Crassostrea gigas* | 0.2 |
|  | **405964679** | **Neurexin-4** | ***Crassostrea gigas*** | **0.5** |
|  | **405960111** | **Neuroglian** | ***Crassostrea gigas*** | **0** |
| **c102881_g1** | **762129353** | **neuroglian-like isoform X1** | ***Crassostrea gigas*** | **0** |
| **c96718_g1** | **762143197** | **neutral ceramidase-like** | ***Crassostrea gigas*** | **0.5** |
| c94770_g1 | 762089386 | nidogen-1-like isoform X1 | *Crassostrea gigas* | 0.1 |
|  | 405966986 | Paramyosin | *Crassostrea gigas* | 0.3 |
| c94503_g2 | 762070443 | pathogen-related protein-like | *Crassostrea gigas* | 0.1 |
|  | 405970466 | Periostin | *Crassostrea gigas* | 0.3 |
|  | 405969430 | Plasma alpha-L-fucosidase | *Crassostrea gigas* | 0 |
|  | 405978156 | Plasma membrane calcium-transporting ATPase 3 | *Crassostrea gigas* | 0.2 |
| c83148_g1 | 762111593 | plasminogen activator inhibitor 1 RNA-binding protein-like isoform X1 | *Crassostrea gigas* | 0.3 |
|  | 405965891 | Prenylcysteine oxidase | *Crassostrea gigas* | 0 |
|  | 405965826 | Programmed cell death protein 4 | *Crassostrea gigas* | 0.2 |
|  | 405968717 | Programmed cell death protein 6 | *Crassostrea gigas* | 0 |
| c90524_g1 | 762125425 | proliferating cell nuclear antigen-like | *Crassostrea gigas* | 0.5 |
| c94819_g1 | 762104009 | prostaglandin reductase 1-like | *Crassostrea gigas* | 0.5 |
|  | 405960660 | Protein disulfide-isomerase A5 | *Crassostrea gigas* | 0.07 |
| c91045_g2 | 762163372 | protein disulfide-isomerase A5-like | *Crassostrea gigas* | 0.05 |
|  | 405965843 | Protein ERGIC-53 | *Crassostrea gigas* | 0 |
|  | 405962525 | Protein FAM63B | *Crassostrea gigas* | 0.4 |
|  | 405969398 | Protein jagged-2 | *Crassostrea gigas* | 0 |
|  | 405972778 | Protein phosphatase 1B | *Crassostrea gigas* | 0.4 |
|  | 405962160 | Protocadherin Fat 4 | *Crassostrea gigas* | 0 |
|  | 405974135 | Putative chitinase 3 | *Crassostrea gigas* | 0 |
|  | 405978194 | Putative sulfite oxidase, mitochondrial | *Crassostrea gigas* | 0.2 |
|  | 405957461 | Putative thiopurine S-methyltransferase | *Crassostrea gigas* | 0.5 |
|  | 405964920 | Pyridoxal-dependent decarboxylase domain-containing protein 1 | *Crassostrea gigas* | 0.3 |
|  | **333449487** | **Ras-like GTP-binding protein RHO** | ***Crassostrea ariakensis*** | **0.4** |
| **c92890_g1** | **762144541** | **ras-like protein 3 isoform X2** | ***Crassostrea gigas*** | **0** |
| **c95272_g1** | **762070124** | **ras-related protein Rab-7a** | ***Crassostrea gigas*** | **0.4** |
|  | 405970353 | Replication protein A 70 kDa DNA-binding subunit | *Crassostrea gigas* | 0.4 |
|  | **405978849** | **Rho GTPase-activating protein 17** | ***Crassostrea gigas*** | **0** |
| c80501_g1 | 405963560 | Sarcoplasmic calcium-binding protein | *Crassostrea gigas* | 0.3 |
|  | 405972994 | SEC13-like protein | *Crassostrea gigas* | 0.3 |
|  | 405973087 | SH3 domain-binding glutamic acid-rich protein | *Crassostrea gigas* | 0.1 |
| c81197_g1 | 762116864 | sodium/potassium-transporting ATPase subunit alpha-like | *Crassostrea gigas* | 0.4 |
|  | 405965139 | Sorting nexin-2 | *Crassostrea gigas* | 0.4 |
|  | 405970223 | Splicing factor 3B subunit 3 | *Crassostrea gigas* | 0.2 |
|  | 405976087 | Splicing factor U2AF 50 kDa subunit | *Crassostrea gigas* | 0 |
|  | 405955028 | Steroid 17-alpha-hydroxylase/17,20 lyase | *Crassostrea gigas* | 0 |
|  | 405963331 | Succinyl-CoA ligase[ADP-forming subunit beta, mitochondrial | *Crassostrea gigas* | 0.4 |
|  | 405971538 | Synaptophysin | *Crassostrea gigas* | 0.4 |
|  | 405950905 | Syntaxin-5 | *Crassostrea gigas* | 0.5 |
|  | **338815381** | **thioredoxin** | ***Crassostrea ariakensis*** | **0.1** |
|  | **405970435** | **Thioredoxin domain-containing protein 5** | ***Crassostrea gigas*** | **0.5** |
| **c98785_g1** | **762080959** | **thioredoxin reductase 1, cytoplasmic-like** | ***Crassostrea gigas*** | **0.4** |
| **c87811_g1** | **762139008** | **thioredoxin-1-like** | ***Crassostrea gigas*** | **0.3** |
| c85190_g1 | 762162716 | THO complex subunit 4-A | *Crassostrea gigas* | 0.2 |
| c90531_g1 | 762118303 | transforming growth factor-beta-induced protein ig-h3-like | *Crassostrea gigas* | 0.4 |
|  | 405976865 | Translocon-associated protein subunit alpha | *Crassostrea gigas* | 0.5 |
|  | 405966687 | Transmembrane emp24 domain-containing protein 1 | *Crassostrea gigas* | 0.4 |
|  | 405950001 | Transmembrane emp24 domain-containing protein 2 | *Crassostrea gigas* | 0 |
|  | 405973883 | transport protein Sec24C | *Crassostrea gigas* | 0.09 |
| c81034_g1 | 405970776 | Troponin C | *Crassostrea gigas* | 0 |
| c83385_g1 | 762145981 | trypsin-1-like | *Crassostrea gigas* | 0 |
|  | 405976987 | Tyrosine-protein phosphatase Lar | *Crassostrea gigas* | 0.05 |
| c102381_g1 | 762129367 | ubiquitin carboxyl-terminal hydrolase 15-like | *Crassostrea gigas* | 0.5 |
| c83003_g1 | 762109320 | ubiquitin-conjugating enzyme E2 N | *Crassostrea gigas* | 0.5 |
|  | 405973130 | UDP-glucose:glycoprotein glucosyltransferase 1 | *Crassostrea gigas* | 0.5 |
| c97935_g1 | 762091582 | universal stress protein A-like protein | *Crassostrea gigas* | 0.3 |
| c83738_g1 | 762091580 | universal stress protein A-like protein | *Crassostrea gigas* | 0 |
|  | 405963822 | UPF0663 transmembrane protein C17orf28 | *Crassostrea gigas* | 0.2 |
| **c102546_g1** | **762087607** | **vinculin-like isoform X7** | ***Crassostrea gigas*** | **0.3** |
| **c91637_g1** | **762151428** | **V-type proton ATPase subunit H-like isoform X3** | ***Crassostrea gigas*** | **0.2** |
|  | 405961750 | Xanthine dehydrogenase | *Crassostrea gigas* | 0.5 |
| c93514_g1 | 762076444 | xylose isomerase-like | *Crassostrea gigas* | 0.3 |
|  | 405966050 | Zinc finger RNA-binding protein | *Crassostrea gigas* | 0 |

Note: INF showed that protein amount in MET was 0.
