# Supplementary material for: Proteome of larval metamorphosis induced by epinephrine in the Fujian oyster Crassostrea angulata
Source: BMC Genomics. 2020 Sep 29;21:675. doi: 10.1186/s12864-020-07066-z (PMC7525975; doi:10.1186/s12864-020-07066-z)
Supplement: Supplementary file 6 — Additional file 6: Supplementary Table 5. The differentially expressed proteins among six groups with protein volumes calculated [file 12864_2020_7066_MOESM6_ESM.doc]

**Supplementary Table 5 The differentially expressed proteins among six groups with protein volumes calculated**

| **Accession Number** | **NR GI** | **Identified Proteins** | **einSEN** | **eMET** | **eSEN** | **MET** | **PA** | **PL** |
| --- | --- | --- | --- | --- | --- | --- | --- | --- |
| c98764_g1 | 405950429 | 14-3-3 protein zeta [*Crassostrea gigas*] | 2.8606 | 10.25713 | 2.6784 | 3.462433 | 5.730767 | 9.3633 |
|  | 405950098 | 14-3-3 protein zeta [*Crassostrea gigas*] | 41.641 | 81.71367 | 47.832 | 57.61467 | 61.046 | 49.6 |
| c86391_g1 | 405973590 | SCO-spondin [*Crassostrea gigas*] | 91.87367 | 463.1067 | 89.38133 | 96.06 | 165.1067 | 89.225 |
|  | 405973589 | SCO-spondin [*Crassostrea gigas*] | 68.67967 | 195.9333 | 84.96533 | 79.183 | 100.258 | 91.828 |
|  | 405966926 | SCO-spondin [*Crassostrea gigas*] | 7.000167 | 36.062 | 5.7061 | 19.80933 | 26.187 | 3.33822 |
| c83004_g1 | 675373238 | SCO-spondin, partial [*Stegodyphus mimosarum*] | 0 | 8.266067 | 1.320667 | 3.042033 | 2.028333 | 0.320187 |
|  | 405953044 | Dynein beta chain, ciliary [*Crassostrea gigas*] | 144.04 | 74.74067 | 146.57 | 19.983 | 116.4367 | 175.3233 |
|  | 405976574 | Dynein beta chain, ciliary [*Crassostrea gigas*] | 0 | 0.331877 | 1.997633 | 0.3382 | 2.458033 | 1.643053 |
|  | 405977373 | Dynein heavy chain 1, axonemal [*Crassostrea gigas*] | 10.80873 | 2.993733 | 20.08433 | 0 | 15.21267 | 31.06233 |
|  | 405950869 | Dynein heavy chain 3, axonemal [*Crassostrea gigas*] | 10.18857 | 3.315733 | 12.052 | 0.3382 | 10.34127 | 12.2 |
|  | 405971451 | Dynein heavy chain 5, axonemal [*Crassostrea gigas*] | 81.37033 | 41.64133 | 79.314 | 0 | 74.49633 | 116.1767 |
|  | 405966381 | Dynein heavy chain 6, axonemal [*Crassostrea gigas*] | 3.188433 | 3.979467 | 3.018133 | 0 | 0.717467 | 8.618133 |
|  | 405969117 | Dynein heavy chain 7, axonemal [*Crassostrea gigas*] | 7.946633 | 1.32549 | 13.106 | 0 | 8.312733 | 29.10933 |
|  | 405963852 | Dynein heavy chain 7, axonemal [*Crassostrea gigas*] | 10.80993 | 2.30032 | 11.74363 | 0 | 4.871733 | 13.60563 |
| c96730_g1 | 762108713 | ubiquilin-1-like [*Crassostrea gigas*] | 2.861633 | 14.24547 | 4.016933 | 11.75497 | 8.9684 | 3.981967 |
| c94432_g1 | 762123240 | ubiquitin carboxyl-terminal hydrolase 14-like [*Crassostrea gigas*] | 12.716 | 18.52433 | 10.7315 | 20.625 | 12.613 | 9.3002 |
| c102381_g1 | 762129367 | ubiquitin carboxyl-terminal hydrolase 15-like [*Crassostrea gigas*] | 2.222333 | 2.974943 | 1.320667 | 5.539167 | 3.436767 | 2.012033 |
| c89055_g1 | 762115659 | ubiquitin carboxyl-terminal hydrolase-like [*Crassostrea gigas*] | 5.404367 | 21.18933 | 8.713267 | 13.70733 | 13.41483 | 8.635967 |
|  | 405969345 | Ubiquitin-associated protein 2 [*Crassostrea gigas*] | 1.588867 | 17.85867 | 1.002967 | 10.5756 | 6.829133 | 0 |
| c83003_g1 | 762109320 | ubiquitin-conjugating enzyme E2 N [*Crassostrea gigas*] | 2.22496 | 3.9696 | 7.703733 | 7.255733 | 5.8105 | 4.9808 |
| c77339_g1 | 762140796 | ubiquitin-conjugating enzyme E2 variant 2-like isoform X1 [*Crassostrea gigas*] | 0.953333 | 5.304967 | 1.000067 | 4 | 5.124033 | 0 |
| c89916_g1 | 762105018 | ubiquitin-conjugating enzyme E2-17 kDa-like [*Crassostrea gigas*] | 1.2669 | 0.327913 | 0 | 0 | 1.076233 | 1.643053 |
|  | 405969825 | E3 ubiquitin-protein ligase HUWE1 [*Crassostrea gigas*] | 0 | 5.946933 | 5.324333 | 0.676433 | 5.832667 | 0.330717 |
|  | 405963851 | IQ and ubiquitin-like domain-containing protein [*Crassostrea gigas*] | 3.499833 | 0.332853 | 6.685767 | 0 | 6.5413 | 5.259333 |
|  | 405964277 | 26S protease regulatory subunit 4 [*Crassostrea gigas*] | 0 | 3.298867 | 0 | 6.904767 | 5.8105 | 3.675633 |
|  | 405957859 | 26S protease regulatory subunit 6A [*Crassostrea gigas*] | 7.9391 | 13.87137 | 17.419 | 26.49467 | 19.29633 | 13.30667 |
|  | 405962781 | 26S proteasome non-ATPase regulatory subunit 3 [*Crassostrea gigas*] | 7.629367 | 1.655413 | 7.039233 | 7.8572 | 3.463367 | 5.966267 |
| c88705_g1 | 762167760 | ATP synthase F(0) complex subunit B1, mitochondrial-like [*Crassostrea gigas*] | 102.3697 | 35.06967 | 95.06833 | 76.09167 | 77.12233 | 101.082 |
|  | 163311503 | ATP synthase F0 subunit 6 [*Crassostrea hongkongensis*] | 6.041533 | 1.32648 | 4.007367 | 1.745833 | 2.692667 | 7.9567 |
| c85397_g1 | 762138531 | ATP synthase mitochondrial F1 complex assembly factor 2-like [*Crassostrea gigas*] | 0 | 4.298467 | 0 | 0.676433 | 6.505833 | 2.0675 |
| c84804_g1 | 762131596 | ATP synthase subunit delta, mitochondrial-like [*Crassostrea gigas*] | 44.22133 | 77.44967 | 38.78333 | 55.366 | 62.49433 | 29.499 |
| c89199_g1 | 762165801 | ATP synthase subunit O, mitochondrial-like [*Crassostrea gigas*] | 13.35967 | 12.912 | 27.79367 | 22.95767 | 32.88367 | 33.68333 |
|  | 405969358 | ATPase family AAA domain-containing protein 2B [*Crassostrea gigas*] | 34.984 | 37.39467 | 42.167 | 17.90633 | 34.27 | 51.79033 |
| c103615_g1 | 762111269 | ATP-citrate synthase-like isoform X1 [*Crassostrea gigas*] | 7.312167 | 3.311767 | 6.373 | 0 | 6.474833 | 8.7434 |
| c12411_g1 | 333449442 | mitochondrial ATP synthase-like protein, partial [Crassostrea ariakensis] | 8.9083 | 55.61767 | 21.412 | 43.39567 | 33.91133 | 23.554 |
|  | 405976318 | 6-phosphogluconate dehydrogenase, decarboxylating [*Crassostrea gigas*] | 8.266967 | 4.949367 | 16.10633 | 6.559233 | 9.295867 | 22.12033 |
| c99129_g1 | 762115224 | D-3-phosphoglycerate dehydrogenase-like, partial [*Crassostrea gigas*] | 4.765133 | 4.632333 | 4.0003 | 5.111467 | 4.8052 | 7.285267 |
| c96381_g1 | 762107263 | enolase-like isoform X2 [*Crassostrea gigas*] | 99.84867 | 82.39333 | 106.05 | 58.73767 | 93.26133 | 110.035 |
| c70507_g1 | 762092826 | enolase-phosphatase E1-like [*Crassostrea gigas*] | 20.66433 | 59.57333 | 29.79433 | 77.347 | 47.55633 | 45.06133 |
|  | 405959171 | Glucose-6-phosphate 1-dehydrogenase [*Crassostrea gigas*] | 0.638733 | 0.988687 | 2.349467 | 0 | 6.1028 | 9.948167 |
| c99968_g1 | 762101323 | glucose-6-phosphate isomerase-like [*Crassostrea gigas*] | 0 | 2.641133 | 0 | 0 | 0 | 1.921133 |
| c89484_g1 | 762103501 | malate dehydrogenase, mitochondrial-like [*Crassostrea gigas*] | 109.68 | 92.97367 | 117.9 | 67.67833 | 96.006 | 130.06 |
| c95972_g1 | 762095264 | putative malate dehydrogenase 1B [*Crassostrea gigas*] | 1.5889 | 0 | 2.330333 | 0 | 2.117 | 1.033733 |
| c100365_g1 | 762114367 | succinate dehydrogenase [ubiquinone] flavoprotein subunit, mitochondrial-like [*Crassostrea gigas*] | 41.96933 | 37.05 | 35.46633 | 22.03233 | 38.87533 | 43.331 |
| c86457_g1 | 762076524 | succinate dehydrogenase [ubiquinone] iron-sulfur subunit, mitochondrial-like [*Crassostrea gigas*] | 3.8155 | 0.655833 | 5.0183 | 1.0968 | 6.846867 | 10.60913 |
| c85806_g1 | 762100869 | succinate dehydrogenase cytochrome b560 subunit, mitochondrial-like isoform X1 [*Crassostrea gigas*] | 3.178367 | 0 | 2.0222 | 0 | 0.983133 | 1.952733 |
|  | 405964920 | Pyridoxal-dependent decarboxylase domain-containing protein 1 [*Crassostrea gigas*] | 0.638733 | 1.639567 | 2.0193 | 4.842667 | 3.126747 | 2.603633 |
|  | 405953655 | Pyruvate dehydrogenase E1 component subunit alpha type II, mitochondrial [*Crassostrea gigas*] | 17.48167 | 7.9392 | 16.73233 | 5.153467 | 13.2642 | 18.221 |
| c87967_g1 | 762115439 | mannose-6-phosphate isomerase-like [*Crassostrea gigas*] | 1.910933 | 1.987233 | 2.337367 | 1.0968 | 1.3729 | 3.35212 |
|  | 405968311 | Isocitrate dehydrogenase [NAD] subunit alpha, mitochondrial [*Crassostrea gigas*] | 3.819733 | 8.622867 | 7.694167 | 14.89167 | 17.04633 | 12.266 |
| c96333_g1 | 762095272 | isocitrate dehydrogenase [NADP] cytoplasmic-like [*Crassostrea gigas*] | 25.43767 | 3.968633 | 14.06767 | 10.7144 | 10.98337 | 19.80167 |
| c82901_g1 | 762091603 | citrate synthase, mitochondrial-like isoform X1 [*Crassostrea gigas*] | 28.62 | 32.09233 | 33.81367 | 15.57533 | 25.72233 | 40.404 |
|  | 405960423 | Cadherin-23 [*Crassostrea gigas*] | 2.5486 | 22.811 | 6.340567 | 17.652 | 8.166733 | 1.670787 |
| c100397_g2 | 762110068 | cadherin-23-like isoform X3 [*Crassostrea gigas*] | 6.038867 | 21.84033 | 10.36163 | 18.01033 | 13.97267 | 5.5762 |
| c101186_g1 | 762110074 | cadherin-87A-like isoform X3 [*Crassostrea gigas*] | 10.81183 | 52.24333 | 13.72367 | 34.87133 | 21.93133 | 3.317167 |
| c100614_g1 | 762091278 | calcium uniporter protein, mitochondrial-like [*Crassostrea gigas*] | 5.732767 | 0 | 5.360933 | 5.484367 | 6.8557 | 5.308133 |
|  | 405964165 | Calcium/calmodulin-dependent protein kinase type II delta chain [*Crassostrea gigas*] | 1.592067 | 0 | 0.9905 | 0 | 1.3729 | 10.60913 |
|  | 405969211 | Calcium-binding mitochondrial carrier protein Aralar1 [*Crassostrea gigas*] | 0.638733 | 0.327913 | 1.009667 | 1.0968 | 0 | 3.059667 |
|  | 405968450 | Calcium-transporting ATPase sarcoplasmic/endoplasmic reticulum type [*Crassostrea gigas*] | 36.56667 | 2.326067 | 9.7152 | 7.552067 | 2.661633 | 13.02113 |
| c90479_g1 | 762086942 | calcium-transporting ATPase sarcoplasmic/endoplasmic reticulum type-like [*Crassostrea gigas*] | 49.6 | 6.952433 | 15.742 | 15.51333 | 4.0035 | 20.99233 |
|  | 405952420 | Calmodulin [*Crassostrea gigas*] | 8.2644 | 24.493 | 12.06033 | 26.19867 | 17.467 | 27.56733 |
|  | 20137620 | RecName: Full=Calmodulin; Short=CaM | 7.630967 | 16.23567 | 7.703733 | 29.53667 | 11.69213 | 24.51467 |
| c55559_g1 | 762161385 | calmodulin-like [*Crassostrea gigas*] | 0 | 0 | 0 | 9.063767 | 0 | 2.655733 |
| c97263_g1 | 405967580 | Calnexin [*Crassostrea gigas*] | 12.71967 | 3.305867 | 10.0412 | 20.963 | 6.1471 | 11.19733 |
|  | 405972360 | Calpain-7-like protein [*Crassostrea gigas*] | 2.861633 | 0.332853 | 2.0222 | 0 | 3.441167 | 3.059667 |
|  | 405964355 | Calpain-A [*Crassostrea gigas*] | 88.70167 | 49.28533 | 87.64967 | 66.15367 | 79.94833 | 90.776 |
|  | 150404776 | calreticulin [Pinctada fucata] | 28.628 | 41.71067 | 27.79267 | 35.067 | 26.92233 | 12.81667 |
| c93407_g1 | 762076798 | caltractin-like [*Crassostrea gigas*] | 0.955453 | 1.311667 | 0 | 1.338233 | 0.655433 | 0.344567 |
| c98322_g1 | 762079826 | calumenin-like isoform X1 [*Crassostrea gigas*] | 0 | 1.986277 | 3.0073 | 12.35617 | 4.805233 | 5.687667 |
| c88952_g1 | 762104881 | calumenin-like isoform X1 [*Crassostrea gigas*] | 0 | 3.981433 | 0 | 3.449633 | 0 | 0 |
| c79422_g1 | 762115410 | EF-hand calcium-binding domain-containing protein 10-like [*Crassostrea gigas*] | 0 | 3.971567 | 0 | 0 | 4.822967 | 2.384333 |
| c101386_g1 | 762133698 | EF-hand calcium-binding domain-containing protein 5-like isoform X1 [*Crassostrea gigas*] | 4.449967 | 4.6303 | 0.334333 | 0 | 0 | 0 |
|  | 405963739 | EF-hand calcium-binding domain-containing protein 6 [*Crassostrea gigas*] | 5.720567 | 3.621867 | 6.009167 | 0 | 5.372 | 6.3175 |
| c81034_g1 | 405970776 | Troponin C [*Crassostrea gigas*] | 0 | 0 | 0 | 2.007333 | 0 | 1.649733 |
| c86784_g5 | 871236028 | troponin I-like [*Aplysia californica*] | 4.1306 | 32.079 | 12.74 | 23.01233 | 27.07733 | 18.93133 |
|  | 405965308 | Troponin T, skeletal muscle [*Crassostrea gigas*] | 41.341 | 113.1667 | 34.43267 | 40.94067 | 50.36833 | 35.10667 |
|  | 405966500 | Cathepsin L [*Crassostrea gigas*] | 14.63233 | 29.44367 | 6.3652 | 36.643 | 19.80967 | 9.017933 |
| c90692_g1 | 762167480 | cathepsin L1-like [*Crassostrea gigas*] | 6.043667 | 6.2838 | 2.6784 | 13.67277 | 9.703467 | 4.2672 |
| c88094_g1 | 762099884 | cathepsin L1-like [*Crassostrea gigas*] | 15.59033 | 32.41167 | 7.368167 | 44.451 | 22.25 | 12.6448 |
| c94508_g1 | 762107740 | cathepsin L1-like [*Crassostrea gigas*] | 6.675467 | 44.03267 | 7.0105 | 39.51133 | 29.57967 | 3.873867 |
| c89919_g1 | 762086974 | cathepsin Z-like [*Crassostrea gigas*] | 26.07833 | 18.85067 | 15.743 | 33.59167 | 22.582 | 14.3999 |
|  | 405966599 | 60 kDa heat shock protein, mitochondrial [*Crassostrea gigas*] | 116.05 | 265.7967 | 161.28 | 348.2067 | 240.6433 | 189.77 |
| c99145_g1 | 762146289 | 60 kDa heat shock protein, mitochondrial-like [*Crassostrea gigas*] | 69.94133 | 185.73 | 96.36533 | 229.28 | 142.7667 | 114.7033 |
| c100704_g2 | 762079683 | heat shock 70 kDa protein 12A-like [*Crassostrea gigas*] | 0.31672 | 0.998567 | 2.338667 | 1.7312 | 3.113467 | 4.3127 |
|  | 405961245 | Heat shock 70 kDa protein 12B [*Crassostrea gigas*] | 0.636093 | 0.998567 | 8.0339 | 1.7312 | 2.037233 | 7.594933 |
|  | 405963608 | Heat shock 70 kDa protein 12B [*Crassostrea gigas*] | 0.638733 | 0 | 0.334333 | 0.676433 | 1.076233 | 1.622 |
| c89955_g1 | 762129389 | heat shock 70 kDa protein 14-like [*Crassostrea gigas*] | 1.90614 | 1.979367 | 1.659167 | 6.575767 | 2.710367 | 1.36445 |
| c101109_g1 | 762084413 | heat shock 70 kDa protein 4-like [*Crassostrea gigas*] | 44.51533 | 61.13267 | 35.08533 | 77.36033 | 60.38167 | 35.911 |
| c82792_g1 | 762131241 | heat shock protein 27-like [*Crassostrea gigas*] | 0 | 2.32412 | 0.330167 | 7.5885 | 1.310867 | 0 |
| c89945_g1 | 762156109 | hsc70-interacting protein-like isoform X1 [*Crassostrea gigas*] | 9.858533 | 29.47833 | 12.71833 | 29.65567 | 18.477 | 15.01233 |
| c95664_g1 | 762130073 | Galectin-6 [*Crassostrea gigas*] | 37.84333 | 18.53933 | 35.80267 | 24.025 | 28.33533 | 25.50333 |
| c89917_g1 | 762163284 | galectin-9-like isoform X2 [*Crassostrea gigas*] | 5.400133 | 2.977933 | 5.007433 | 2.138833 | 4.827367 | 6.940667 |
| c67896_g1 | 762109657 | hepatic lectin-like [*Crassostrea gigas*] | 1.906677 | 4.6353 | 1.320667 | 3.062133 | 1.74498 | 3.303333 |
| c94262_g1 | 762101734 | IgGFc-binding protein-like [*Crassostrea gigas*] | 2.5444 | 20.817 | 6.015867 | 21.65767 | 7.551067 | 5.6178 |
| c97935_g1 | 762091582 | universal stress protein A-like protein [*Crassostrea gigas*] | 26.07667 | 3.305867 | 27.80633 | 12.5005 | 19.5 | 20.90833 |
| c83738_g1 | 762091580 | universal stress protein A-like protein [*Crassostrea gigas*] | 0 | 0 | 4.360867 | 1.3309 | 0.717467 | 2.5615 |
| c89143_g1 | 762091586 | universal stress protein A-like protein [*Crassostrea gigas*] | 9.2153 | 0 | 6.683967 | 0 | 3.737867 | 15.58667 |
| c86426_g1 | 762094049 | universal stress protein A-like protein [*Crassostrea gigas*] | 9.536133 | 4.298467 | 9.7136 | 4.146133 | 4.065567 | 11.31953 |
| c92715_g1 | 762091592 | universal stress protein A-like protein isoform X2 [*Crassostrea gigas*] | 3.8171 | 4.630333 | 6.672033 | 4.497167 | 4.504133 | 14.89733 |
| c95884_g1 | 762089183 | superoxide dismutase [Cu-Zn]-like isoform X1 [*Crassostrea gigas*] | 26.707 | 60.57067 | 28.799 | 50.32967 | 30.86367 | 22.80567 |
| c78713_g1 | 821595281 | superoxide dismutase [Mn], mitochondrial-like [*Crassostrea gigas*] | 6.364633 | 0 | 8.0285 | 0 | 0.983133 | 4.6467 |
| c83840_g1 | 762070756 | glutathione S-transferase A-like [*Crassostrea gigas*] | 3.497733 | 2.983867 | 3.3512 | 3.773233 | 2.054933 | 8.569433 |
| c85813_g1 | 762128365 | glutathione S-transferase omega-1-like [*Crassostrea gigas*] | 4.446267 | 5.42 | 5.375933 | 5.822567 | 7.196733 | 6.6205 |
| c87390_g1 | 762156004 | glutathione S-transferase P 1-like [*Crassostrea gigas*] | 3.8139 | 6.9515 | 11.0605 | 9.603067 | 12.00667 | 13.26167 |
|  | 405959835 | Catalase [*Crassostrea gigas*] | 27.65867 | 22.155 | 30.45133 | 8.5884 | 20.824 | 24.633 |
| c88602_g1 | 405975361 | eosinophil peroxidase-like isoform X2 [*Crassostrea gigas*] | 53.12967 | 44.97633 | 51.56133 | 0 | 6.111667 | 38.65333 |
|  | 405962230 | Peroxidasin [*Crassostrea gigas*] | 4.461667 | 8.2718 | 12.47867 | 0 | 0.717467 | 13.32767 |
|  | 405962229 | Peroxidasin-like protein [*Crassostrea gigas*] | 39.43533 | 85.049 | 65.655 | 0 | 21.58133 | 75.323 |
|  | 405972926 | Peroxiredoxin-4 [*Crassostrea gigas*] | 25.75533 | 41.35633 | 25.132 | 46.89267 | 37.401 | 23.551 |
|  | 405974897 | Peroxiredoxin-5, mitochondrial [*Crassostrea gigas*] | 41.971 | 86.059 | 33.849 | 42.43467 | 48.96867 | 29.13333 |
| c86938_g1 | 405953294 | Kyphoscoliosis peptidase [*Crassostrea gigas*] | 2.228187 | 5.622 | 3.334933 | 3.131533 | 6.846867 | 4.844867 |
|  | 405972462 | Kyphoscoliosis peptidase [*Crassostrea gigas*] | 4.1322 | 4.6353 | 3.347033 | 6.5465 | 3.7999 | 0.992167 |
| c75090_g1 | 762085934 | radial spoke head 1 homolog [*Crassostrea gigas*] | 24.47333 | 46.666 | 16.046 | 4.082167 | 23.065 | 9.961633 |
| c91646_g1 | 762084810 | radial spoke head protein 3 homolog B-like [*Crassostrea gigas*] | 22.257 | 46.668 | 26.77433 | 23.453 | 34.044 | 21.59833 |
| c70729_g1 | 762119151 | radial spoke head protein 4 homolog A-like [*Crassostrea gigas*] | 27.343 | 82.07767 | 29.79533 | 31.952 | 62.58733 | 46.79433 |
| c76439_g1 | 762133006 | radial spoke head protein 9 homolog [*Crassostrea gigas*] | 20.02567 | 10.24943 | 16.72533 | 9.305067 | 17.396 | 20.79 |
| c100749_g1 | 762156704 | MAP kinase-activated protein kinase 2-like [*Crassostrea gigas*] | 4.7667 | 3.294967 | 4.677267 | 3.021967 | 2.152433 | 4.9913 |
| **c97912_g1** | **762107124** | **mitogen-activated protein kinase 1-like [*Crassostrea gigas*]** | **1.5889** | **0** | **2.682533** | **0** | **3.082433** | **3.3765** |
| c86391_g2 | 762085926 | mucin-17-like [*Crassostrea gigas*] | 35.93033 | 104.2333 | 34.129 | 45.49267 | 47.71067 | 34.10067 |
| c11357_g1 | 762080102 | mucin-19-like [*Crassostrea gigas*] | 2.861633 | 4.950333 | 0.660333 | 0 | 0.686447 | 0 |
| c134536_g1 | 762077014 | mucin-2-like [*Crassostrea gigas*] | 0 | 3.295953 | 0 | 1.773233 | 1.966267 | 0 |
| c7692_g1 | 871278596 | mucin-5AC-like [Aplysia californica] | 0 | 8.262167 | 1.019233 | 4.800633 | 4.1099 | 0 |
| c98108_g1 | 762085928 | mucin-5AC-like [*Crassostrea gigas*] | 27.98 | 87.03967 | 31.105 | 33.19867 | 40.02633 | 22.064 |
| c102473_g1 | 762076941 | mucin-5AC-like [*Crassostrea gigas*] | 0 | 5.946933 | 0 | 4.698367 | 1.324167 | 0 |
| c95895_g1 | 762099838 | mucin-5AC-like [*Crassostrea gigas*] | 0 | 7.2764 | 0 | 5.531867 | 1.023067 | 0 |
| c91752_g1 | 762100460 | mucin-like protein [*Crassostrea gigas*] | 1.588867 | 0.998567 | 0.660333 | 0 | 16.577 | 1.9843 |
| c100846_g1 | 762160275 | V-type proton ATPase catalytic subunit A isoform X1 [*Crassostrea gigas*] | 38.48533 | 28.458 | 25.07267 | 20.35767 | 26.329 | 28.20767 |
| c93981_g1 | 405950693 | V-type proton ATPase subunit E [*Crassostrea gigas*] | 0.958133 | 7.9431 | 4.007367 | 4.504467 | 3.751167 | 2.986467 |
| c91637_g1 | 762151428 | V-type proton ATPase subunit H-like isoform X3 [*Crassostrea gigas*] | 15.2443 | 1.975433 | 14.707 | 8.869967 | 9.659233 | 14.626 |
| c96390_g1 | 762145704 | V-type proton ATPase subunit S1-like [*Crassostrea gigas*] | 0.953333 | 5.2921 | 0.668667 | 5.126067 | 2.785747 | 0.330717 |
|  | 405976660 | Ran GTPase-activating protein 1 [*Crassostrea gigas*] | 0 | 7.951 | 0 | 8.981467 | 5.128533 | 0 |
| c95388_g1 | 762105818 | ran-specific GTPase-activating protein-like [*Crassostrea gigas*] | 5.721633 | 12.9021 | 6.006267 | 18.23733 | 8.556533 | 5.6666 |
|  | 333449487 | Ras-like GTP-binding protein RHO [Crassostrea ariakensis] | 9.851667 | 3.291033 | 10.3696 | 8.866 | 2.75028 | 5.927467 |
| c92890_g1 | 762144541 | ras-like protein 3 isoform X2 [*Crassostrea gigas*] | 3.498767 | 0 | 4.0157 | 3.435033 | 2.427013 | 5.273233 |
|  | 405976260 | Ras-related protein Rab-10 [*Crassostrea gigas*] | 7.306267 | 4.624433 | 7.038 | 5.870067 | 6.775933 | 13.91567 |
| c97767_g2 | 762121318 | ras-related protein Rab-14 [*Crassostrea gigas*] | 4.450533 | 1.643543 | 5.6761 | 1.380233 | 5.119667 | 10.2784 |
| c83633_g1 | 762141921 | ras-related protein Rab-35-like [*Crassostrea gigas*] | 6.677633 | 4.296533 | 5.689833 | 3.765933 | 2.652733 | 13.57133 |
| c95272_g1 | 762070124 | ras-related protein Rab-7a [*Crassostrea gigas*] | 13.033 | 5.614067 | 8.7068 | 13.33443 | 9.858433 | 12.31153 |
|  | 405978849 | Rho GTPase-activating protein 17 [*Crassostrea gigas*] | 0 | 0 | 2.311167 | 3.802467 | 0 | 1.378333 |
| c86843_g1 | 762100962 | soma ferritin-like [*Crassostrea gigas*] | 3.180433 | 5.950933 | 0.334333 | 0.330897 | 0.717467 | 0 |
| c93420_g1 | 762126464 | cold shock domain-containing protein 3-like [*Crassostrea gigas*] | 66.75567 | 60.86733 | 46.18567 | 29.94467 | 26.10767 | 10.49767 |
| c99017_g1 | 762122989 | spectrin alpha chain-like isoform X6 [*Crassostrea gigas*] | 7.629367 | 371.9067 | 27.445 | 182.64 | 161.2267 | 76.495 |
|  | 405961963 | Spectrin beta chain [*Crassostrea gigas*] | 23.53367 | 48.28 | 50.14467 | 50.445 | 59.558 | 58.96733 |
| c75378_g1 | 762102752 | cilia- and flagella-associated protein 20 [*Crassostrea gigas*] | 9.2184 | 5.965767 | 14.37033 | 3.442333 | 8.915267 | 21.91433 |
| c99303_g1 | 762156177 | cilia- and flagella-associated protein 61-like [*Crassostrea gigas*] | 5.7195 | 4.622467 | 5.360933 | 0 | 5.1817 | 2.996967 |
|  | 405966858 | 3-hydroxyacyl-CoA dehydrogenase type-2 [*Crassostrea gigas*] | 13.35133 | 10.57447 | 13.69067 | 5.331033 | 13.645 | 12.27667 |
|  | 405955617 | 3-hydroxyanthranilate 3,4-dioxygenase [*Crassostrea gigas*] | 4.1306 | 6.615667 | 5.688567 | 20.99033 | 9.956 | 6.672633 |
|  | 405964878 | 40S ribosomal protein S11 [*Crassostrea gigas*] | 4.135367 | 6.3006 | 6.0446 | 3.413067 | 5.1507 | 17.856 |
|  | 405964154 | 40S ribosomal protein S9 [*Crassostrea gigas*] | 4.772 | 12.90733 | 8.368233 | 7.146133 | 8.7867 | 10.84223 |
|  | 405963175 | 60 kDa neurofilament protein [*Crassostrea gigas*] | 46.42667 | 27.44833 | 37.84133 | 50.98233 | 34.16333 | 23.49433 |
|  | 405965820 | 60S ribosomal protein L26 [*Crassostrea gigas*] | 1.914643 | 0 | 0.9905 | 0 | 0.717467 | 0.320187 |
|  | 405965901 | 60S ribosomal protein L27a [*Crassostrea gigas*] | 5.723233 | 8.606833 | 1.002967 | 0 | 1.6785 | 1.93165 |
|  | 405959119 | 60S ribosomal protein L3, partial [*Crassostrea gigas*] | 10.8028 | 9.577933 | 8.387267 | 3.007333 | 14.243 | 21.159 |
|  | 405977927 | 60S ribosomal protein L9 [*Crassostrea gigas*] | 1.904533 | 7.281367 | 5.369233 | 3.360167 | 4.752033 | 4.6046 |
|  | 405960426 | 4-aminobutyrate aminotransferase, mitochondrial [*Crassostrea gigas*] | 3.181 | 0.998567 | 4.359233 | 0.3656 | 3.410167 | 4.671133 |
| c85361_g1 | 762086880 | 4-hydroxyphenylpyruvate dioxygenase [*Crassostrea gigas*] | 2.86372 | 0.995633 | 7.012233 | 0.6618 | 0 | 5.8925 |
|  | 405974270 | 5'-AMP-activated protein kinase subunit beta-2 [*Crassostrea gigas*] | 14.625 | 24.15833 | 7.029667 | 17.12933 | 20.83733 | 6.303667 |
| c98089_g1 | 762110625 | A disintegrin and metalloproteinase with thrombospondin motifs 6-like [*Crassostrea gigas*] | 18.44767 | 34.409 | 13.36333 | 28.99767 | 12.55993 | 15.77433 |
|  | 405958039 | Acidic leucine-rich nuclear phosphoprotein 32 family member A [*Crassostrea gigas*] | 11.77923 | 22.49333 | 14.709 | 43.86 | 21.001 | 24.86633 |
|  | 405968537 | Actin [*Crassostrea gigas*] | 9.219667 | 15.9278 | 1.67 | 2.131497 | 2.444713 | 0 |
|  | 405974071 | Actin [*Crassostrea gigas*] | 14.25233 | 11.477 | 28.75533 | 23.10733 | 0 | 15.735 |
|  | 405969755 | Actin-3 [*Crassostrea gigas*] | 15.547 | 23.83533 | 23.76333 | 16.782 | 3.946 | 10.03233 |
| c93725_g3 | 527271971 | acyl-CoA-binding protein [Melopsittacus undulatus] | 0.317777 | 6.624533 | 0 | 0 | 0.358733 | 1.695167 |
|  | 405972978 | Adenylosuccinate synthetase [*Crassostrea gigas*] | 1.5889 | 0 | 1.3373 | 0 | 1.417267 | 4.618967 |
|  | 405961802 | ADP-ribosylation factor [*Crassostrea gigas*] | 7.954467 | 10.2543 | 9.038133 | 8.7406 | 8.968167 | 17.20533 |
| c101293_g1 | 762096787 | alanine aminotransferase 1-like [*Crassostrea gigas*] | 2.219167 | 4.298567 | 4.3567 | 0 | 3.140067 | 4.030733 |
| c95371_g1 | 762112709 | alcohol dehydrogenase class-3-like [*Crassostrea gigas*] | 16.85767 | 17.54367 | 25.08833 | 14.36733 | 15.68667 | 28.21167 |
| c99122_g1 | 762095292 | aldehyde dehydrogenase family 3 member B1-like [*Crassostrea gigas*] | 0.317777 | 0 | 0.668667 | 0 | 0 | 1.952733 |
|  | 405962156 | Alpha-aminoadipic semialdehyde synthase, mitochondrial [*Crassostrea gigas*] | 1.58782 | 1.652453 | 1.677033 | 1.3236 | 5.7928 | 7.041467 |
| c90043_g1 | 762146639 | alpha-amylase-like [*Crassostrea gigas*] | 2.5444 | 1.661333 | 0 | 0 | 0 | 0 |
|  | 405961891 | Alpha-crystallin B chain [*Crassostrea gigas*] | 3.49294 | 0.660767 | 2.0139 | 1.34553 | 2.3871 | 4.671133 |
| c100364_g1 | 762162806 | alpha-L-fucosidase-like [*Crassostrea gigas*] | 2.864267 | 1.980353 | 2.348233 | 4.497167 | 5.837133 | 5.6666 |
| c92241_g2 | 762101727 | alpha-L-fucosidase-like isoform X2 [*Crassostrea gigas*] | 4.766167 | 2.979953 | 3.362033 | 5.807933 | 2.382633 | 6.000667 |
|  | 405970312 | Alpha-N-acetylgalactosaminidase [*Crassostrea gigas*] | 52.446 | 42.66 | 50.52267 | 87.15767 | 47.37933 | 43.80533 |
| c102224_g1 | 762151951 | alpha-N-acetylglucosaminidase-like isoform X2 [*Crassostrea gigas*] | 8.897867 | 14.206 | 6.695367 | 19.49867 | 15.03567 | 11.33303 |
|  | 405954380 | Alpha-soluble NSF attachment protein [*Crassostrea gigas*] | 0 | 0 | 0 | 2.367467 | 0 | 1.033733 |
|  | 405977952 | Aminopeptidase N [*Crassostrea gigas*] | 10.17943 | 0 | 7.687567 | 16.762 | 3.228667 | 7.9468 |
|  | 405976514 | Amyloid protein-binding protein 2 [*Crassostrea gigas*] | 0.31672 | 0 | 0 | 0 | 0.668747 | 1.006 |
|  | 405971772 | Angiotensin-converting enzyme [*Crassostrea gigas*] | 13.032 | 25.482 | 18.76533 | 31.16567 | 23.304 | 16.27567 |
|  | 405976099 | Annexin A7 [*Crassostrea gigas*] | 5.0834 | 7.285367 | 9.391833 | 8.3251 | 8.2198 | 8.691333 |
|  | 405956161 | Annexin A7 [*Crassostrea gigas*] | 4.447867 | 5.630867 | 8.373633 | 6.917533 | 6.164833 | 5.704867 |
| c80803_g1 | 405970526 | Annexin A7, partial [*Crassostrea gigas*] | 5.723767 | 0 | 1.682467 | 1.014633 | 1.740533 | 2.7012 |
|  | 405961595 | Anosmin-1 [*Crassostrea gigas*] | 0 | 1.661333 | 0 | 3.007333 | 1.434967 | 0 |
|  | 405962570 | AP-2 complex subunit alpha-2 [*Crassostrea gigas*] | 8.908333 | 0.65979 | 3.6735 | 0.3382 | 1.076233 | 10.92913 |
|  | 405971077 | AP-2 complex subunit beta [*Crassostrea gigas*] | 52.791 | 41.02533 | 64.90167 | 69.982 | 80.01733 | 63.04667 |
| c89235_g1 | 405951507 | AP-2 complex subunit mu-1 [*Crassostrea gigas*] | 4.7694 | 0.983733 | 2.005967 | 0 | 0 | 3.939833 |
| c93079_g1 | 762165574 | apoptosis-inducing factor 3-like isoform X1 [*Crassostrea gigas*] | 2.222873 | 1.31561 | 0 | 0.330897 | 1.731667 | 1.632533 |
| c80501_g2 | 762073926 | arcoplasmic calcium-binding protein-like isoform X2 [*Crassostrea gigas*] | 1.27166 | 16.562 | 8.7231 | 21.45133 | 14.327 | 37.33033 |
|  | 405975706 | Band 4.1-like protein 3 [*Crassostrea gigas*] | 2.858967 | 13.575 | 3.022267 | 2.422233 | 6.842433 | 3.574733 |
| c90671_g1 | 762136815 | beta-catenin-like protein 1 [*Crassostrea gigas*] | 1.2775 | 0 | 3.3566 | 0 | 0 | 3.675633 |
|  | 405965163 | Beta-hexosaminidase subunit beta [*Crassostrea gigas*] | 25.103 | 11.59973 | 21.119 | 32.11633 | 15.36767 | 20.90533 |
|  | 533221120 | beta-mannosidase [Stenotrophomonas maltophilia MF89] | 1.269 | 0 | 1.0084 | 1.0694 | 2.710367 | 2.311167 |
|  | 56603670 | beta-tubulin [*Crassostrea gigas*] | 1831.933 | 841.5033 | 1609.467 | 710.2 | 1198.167 | 1463.367 |
|  | 405967658 | Bifunctional aminoacyl-tRNA synthetase [*Crassostrea gigas*] | 7.959 | 0 | 5.384267 | 4.787867 | 2.816767 | 6.675967 |
| c102644_g1 | 762138581 | C-1-tetrahydrofolate synthase, cytoplasmic-like [*Crassostrea gigas*] | 6.996467 | 7.9491 | 5.350067 | 8.670633 | 12.37833 | 13.30667 |
|  | 405972424 | cAMP-dependent protein kinase type II regulatory subunit [*Crassostrea gigas*] | 13.04263 | 7.274467 | 10.02863 | 13.153 | 14.32267 | 8.9594 |
|  | 405953236 | Carbonic anhydrase [*Crassostrea gigas*] | 1.911467 | 0.6657 | 0.339733 | 7.7402 | 0 | 0.640367 |
| c97264_g1 | 762104782 | carbonic anhydrase 2-like [*Crassostrea gigas*] | 9.540167 | 4.292533 | 12.37267 | 0 | 0 | 10.1674 |
|  | 405974400 | Carbonyl reductase [NADPH] 1 [*Crassostrea gigas*] | 3.182033 | 3.293967 | 6.045833 | 4.7403 | 0.717467 | 15.20733 |
| c102687_g1 | 762092188 | CD109 antigen-like [*Crassostrea gigas*] | 9.222 | 5.302967 | 6.725467 | 20.01067 | 11.2182 | 4.302133 |
| c89963_g1 | 762146607 | CD109 antigen-like [*Crassostrea gigas*] | 19.71567 | 32.43433 | 17.71067 | 36.33367 | 21.30233 | 13.95067 |
| c91048_g1 | 762121724 | cell migration-inducing and hyaluronan-binding protein-like [*Crassostrea gigas*] | 0 | 2.640153 | 0 | 3.308967 | 0 | 0.664753 |
| c91059_g3 | 762099416 | chitinase-3-like protein 1 isoform X3 [*Crassostrea gigas*] | 26.077 | 28.12667 | 21.81233 | 7.884667 | 28.55667 | 26.44633 |
|  | 405974135 | Putative chitinase 3 [*Crassostrea gigas*] | 3.178367 | 0 | 0.668667 | 2.80793 | 2.723667 | 2.00152 |
| c75751_g1 | 762149469 | chondroitin proteoglycan 2-like isoform X1 [*Crassostrea gigas*] | 1.904533 | 7.281367 | 2.0222 | 2.000033 | 4.1188 | 1.9943 |
| c93437_g1 | 762122492 | clathrin heavy chain 1 isoform X3 [*Crassostrea gigas*] | 144.6667 | 79.685 | 152.5367 | 117.0667 | 117.2233 | 161.5667 |
|  | 405963691 | Cofilin [*Crassostrea gigas*] | 14.93667 | 7.9372 | 21.11367 | 23.13667 | 16.99767 | 21.41 |
|  | 405959610 | Coiled-coil domain-containing protein 81 [*Crassostrea gigas*] | 0 | 0 | 0.9905 | 0 | 2.152433 | 2.039767 |
|  | 405954419 | Collagen alpha-3(VI) chain [*Crassostrea gigas*] | 3.8091 | 5.272333 | 3.347033 | 32.118 | 6.784833 | 2.603633 |
|  | 405975735 | Collagen alpha-5(VI) chain [*Crassostrea gigas*] | 55.33 | 47.98967 | 31.422 | 49.09067 | 38.58767 | 24.71633 |
|  | 405961982 | Collagen alpha-5(VI) chain [*Crassostrea gigas*] | 37.50733 | 0 | 10.07117 | 31.51133 | 1.7228 | 12.57233 |
|  | 405954309 | Constitutive coactivator of PPAR-gamma-like protein 1-like protein [*Crassostrea gigas*] | 4.1338 | 0.327913 | 5.0295 | 0.330897 | 1.6829 | 6.9651 |
|  | 405975071 | Coronin-1B [*Crassostrea gigas*] | 28.93933 | 25.14267 | 37.82967 | 26.99967 | 36.88267 | 46.63833 |
|  | 405974697 | C-terminal-binding protein [*Crassostrea gigas*] | 5.4065 | 0.993613 | 4.016933 | 3.1242 | 4.127633 | 2.311167 |
|  | 405975170 | CUB and sushi domain-containing protein 1 [*Crassostrea gigas*] | 0.953333 | 9.899767 | 0.668667 | 5.042033 | 4.814067 | 0.640367 |
| c28847_g1 | 762072289 | cubilin-like [*Crassostrea gigas*] | 1.906677 | 12.24467 | 2.009733 | 10.27037 | 9.198667 | 1.649733 |
| c101114_g1 | 762113070 | cystathionine beta-synthase-like isoform X6 [*Crassostrea gigas*] | 2.2255 | 0 | 0.339733 | 1.71113 | 2.037233 | 0.992167 |
|  | 405950302 | Cystatin-A [*Crassostrea gigas*] | 45.15033 | 34.071 | 49.235 | 21.99767 | 41.077 | 49.73967 |
|  | 405959611 | Cystatin-B [*Crassostrea gigas*] | 9.543867 | 8.939633 | 5.008733 | 10.16623 | 5.3853 | 5.913633 |
|  | 229324834 | cytochrome b [Crassostrea angulata] | 2.549153 | 0 | 2.0139 | 0 | 0 | 3.2961 |
| c77693_g1 | 765826145 | cytochrome b-c1 complex subunit Rieske, mitochondrial-like [*Crassostrea gigas*] | 6.0427 | 0 | 10.0825 | 2.014633 | 12.684 | 13.28567 |
|  | 187762792 | cytochrome c oxidase subunit 1 [*Crassostrea gigas*] | 13.36283 | 1.6544 | 7.3309 | 1.34553 | 0.327713 | 6.609933 |
|  | 229324835 | cytochrome c oxidase subunit II [Crassostrea angulata] | 26.70033 | 1.31561 | 23.41767 | 7.484333 | 8.662767 | 21.56967 |
|  | 405951857 | Cytochrome c1, heme protein, mitochondrial [*Crassostrea gigas*] | 20.03167 | 11.25613 | 19.40133 | 9.188367 | 18.53 | 22.371 |
|  | 405976185 | Deleted in malignant brain tumors 1 protein [*Crassostrea gigas*] | 19.72433 | 88.41133 | 22.07533 | 48.50733 | 46.64833 | 16.65117 |
|  | 405972975 | Deleted in malignant brain tumors 1 protein [*Crassostrea gigas*] | 0 | 11.5848 | 0 | 1.3656 | 2.063833 | 0 |
|  | 405966262 | Deleted in malignant brain tumors 1 protein [*Crassostrea gigas*] | 7.632567 | 0 | 1.3373 | 8.9193 | 2.333933 | 0.689167 |
|  | 405966631 | Deleted in malignant brain tumors 1 protein [*Crassostrea gigas*] | 0 | 1.975433 | 0 | 1.676433 | 2.754733 | 0 |
| c88531_g1 | 405965483 | Dihydrolipoyl dehydrogenase, mitochondrial [*Crassostrea gigas*] | 22.892 | 13.22433 | 16.723 | 9.195667 | 16.67867 | 29.59333 |
|  | 405965494 | DnaJ-like protein subfamily B member 11 [*Crassostrea gigas*] | 8.8959 | 1.664267 | 9.3763 | 17.91367 | 3.370267 | 2.979267 |
| c90254_g1 | 762155518 | drebrin-like protein B isoform X2 [*Crassostrea gigas*] | 1.2669 | 3.3137 | 0.668667 | 6.877367 | 0.358733 | 0.320187 |
|  | 405975234 | Dual oxidase 2, partial [*Crassostrea gigas*] | 4.131167 | 1.316587 | 1.3373 | 4.939467 | 4.791967 | 0.960567 |
| c86615_g1 | 762130855 | EF-hand domain-containing family member C2-like [*Crassostrea gigas*] | 35.29433 | 2.310233 | 27.073 | 0.3656 | 13.44137 | 39.785 |
| c82826_g1 | 762138085 | EF-hand domain-containing protein 1-like [*Crassostrea gigas*] | 32.75333 | 2.6599 | 39.85967 | 1.352833 | 18.45433 | 41.726 |
|  | 405968675 | EF-hand domain-containing protein D1 [*Crassostrea gigas*] | 12.71733 | 5.619087 | 11.02737 | 23.93 | 10.83277 | 7.330033 |
|  | 405965462 | Elongation factor Tu, mitochondrial [*Crassostrea gigas*] | 17.80267 | 10.58513 | 15.39233 | 5.780533 | 11.99317 | 20.477 |
|  | 405971816 | Endoplasmic reticulum aminopeptidase 1 [*Crassostrea gigas*] | 3.1794 | 0 | 1.330233 | 6.8573 | 1.076233 | 1.9943 |
| c85729_g1 | 762142476 | epsin-2-like isoform X1 [*Crassostrea gigas*] | 0 | 3.971587 | 0 | 1.0968 | 1.023067 | 0 |
| c92449_g2 | 762129765 | ER membrane protein complex subunit 10-like isoform X1 [*Crassostrea gigas*] | 0 | 3.312743 | 2.3578 | 3.718467 | 4.468633 | 1.695167 |
|  | 405970058 | Eukaryotic translation initiation factor 3 subunit A [*Crassostrea gigas*] | 2.225033 | 0 | 4.016933 | 2.815233 | 3.423433 | 5.026233 |
|  | 405951422 | Eukaryotic translation initiation factor 3 subunit G-A [*Crassostrea gigas*] | 3.497733 | 1.977367 | 4.016933 | 8.6231 | 6.5103 | 6.623833 |
|  | 405966738 | Eukaryotic translation initiation factor 4 gamma 3 [*Crassostrea gigas*] | 6.042633 | 8.6029 | 4.6952 | 6.1315 | 5.549267 | 2.012033 |
|  | 405975002 | Eukaryotic translation initiation factor 6 [*Crassostrea gigas*] | 11.44733 | 21.188 | 8.6984 | 19.321 | 17.73267 | 7.681833 |
|  | 405968987 | Fatty acid-binding-like protein 5 [*Crassostrea gigas*] | 7.311067 | 4.6422 | 6.3735 | 13.3051 | 10.35033 | 4.291633 |
| c92454_g1 | 762156349 | F-box only protein 21-like [*Crassostrea gigas*] | 2.856307 | 29.09967 | 10.73567 | 6.305067 | 17.569 | 12.24483 |
| c87781_g1 | 762131945 | F-box only protein 36-like [*Crassostrea gigas*] | 13.359 | 2.650033 | 13.055 | 0.676433 | 6.4261 | 6.637667 |
| c83242_g1 | 762141095 | F-box/LRR-repeat protein 3-like [*Crassostrea gigas*] | 10.193 | 14.517 | 9.426333 | 4.632667 | 18.02067 | 13.56767 |
|  | 405963678 | Fibropellin-1 [*Crassostrea gigas*] | 11.76077 | 58.9 | 8.3299 | 39.621 | 36.555 | 9.8712 |
| c100882_g1 | 762105167 | flotillin-1-like isoform X4 [*Crassostrea gigas*] | 17.804 | 0.988687 | 8.9889 | 7.539167 | 3.764467 | 10.94687 |
| c94435_g1 | 762097616 | flotillin-2a-like [*Crassostrea gigas*] | 34.34167 | 2.3152 | 17.74067 | 13.74733 | 5.093033 | 21.267 |
|  | 405960295 | Fumarylacetoacetase [*Crassostrea gigas*] | 9.535 | 4.962233 | 10.73677 | 4.084067 | 13.00733 | 20.32767 |
| c98882_g1 | 762080824 | galactokinase-like [*Crassostrea gigas*] | 17.79733 | 0.663767 | 18.434 | 10.645 | 7.5156 | 17.877 |
|  | 405970485 | Ganglioside GM2 activator [*Crassostrea gigas*] | 19.71067 | 30.10233 | 18.757 | 37.904 | 24.38467 | 14.92167 |
| c100029_g2 | 762099370 | gastric intrinsic factor-like [*Crassostrea gigas*] | 24.164 | 31.097 | 29.768 | 62.55967 | 30.129 | 22.89633 |
| c97708_g1 | 762072670 | GDP-L-fucose synthase-like [*Crassostrea gigas*] | 0.952287 | 0 | 3.344133 | 0 | 0 | 3.045767 |
| c92723_g1 | 762074236 | gelsolin-like protein 2 [*Crassostrea gigas*] | 27.02233 | 19.50433 | 31.41767 | 15.6305 | 31.43533 | 26.77 |
| c95005_g1 | 762089402 | gelsolin-like protein 2 [*Crassostrea gigas*] | 122.1067 | 269.74 | 118.5967 | 164.5 | 144.9067 | 95.671 |
| c82585_g2 | 762145041 | glia maturation factor beta-like [*Crassostrea gigas*] | 3.812833 | 0.327913 | 3.6772 | 4.7933 | 4.809667 | 3.651267 |
|  | 405973352 | Glucose-repressible alcohol dehydrogenase transcriptional effector [*Crassostrea gigas*] | 4.1381 | 3.637733 | 0 | 6.332433 | 1.403947 | 0.689167 |
| c90196_g1 | 762099264 | glucosidase 2 subunit beta-like isoform X2 [*Crassostrea gigas*] | 0 | 7.267533 | 0.668667 | 2.345533 | 3.839833 | 1.622 |
| c96690_g1 | 762147159 | glutamate dehydrogenase, mitochondrial-like [*Crassostrea gigas*] | 55.33467 | 53.95633 | 61.91867 | 32.30667 | 46.099 | 79.869 |
|  | 405969003 | Glutamate synthase [NADH], amyloplastic [*Crassostrea gigas*] | 7.001233 | 10.58533 | 6.354333 | 5.815267 | 15.88167 | 10.99583 |
| c99891_g3 | 762127166 | glutamine--fructose-6-phosphate aminotransferase [isomerizing] 1-like isoform X3 [*Crassostrea gigas*] | 25.75367 | 15.87433 | 29.10833 | 21.81167 | 21.701 | 31.183 |
|  | 405973024 | Glutaredoxin-3 [*Crassostrea gigas*] | 5.4097 | 5.616067 | 6.366433 | 11.29763 | 8.543233 | 7.2886 |
| c96570_g1 | 762134867 | glutathione reductase, mitochondrial-like [*Crassostrea gigas*] | 1.2743 | 1.65738 | 1.678333 | 1.393 | 4.4465 | 0.640367 |
| c98484_g1 | 405964519 | glycerol-3-phosphate dehydrogenase [NAD(+)], cytoplasmic-like [*Crassostrea gigas*] | 13.34773 | 6.926767 | 14.02667 | 18.99767 | 14.12367 | 16.78733 |
| c102113_g1 | 762136488 | glycerol-3-phosphate dehydrogenase, mitochondrial-like isoform X2 [*Crassostrea gigas*] | 0.317777 | 0 | 3.3579 | 0 | 3.117867 | 4.692167 |
|  | 405965040 | Glycogen phosphorylase, muscle form [*Crassostrea gigas*] | 34.34633 | 28.121 | 51.87333 | 23.076 | 44.60633 | 68.72033 |
| c102976_g1 | 762100833 | glycogen phosphorylase, muscle form-like isoform X1 [*Crassostrea gigas*] | 32.43833 | 25.47767 | 46.85233 | 20.318 | 44.62 | 71.801 |
|  | 56718386 | glycogen synthase [*Crassostrea gigas*] | 3.496667 | 0 | 5.670667 | 2.193633 | 2.807867 | 7.247 |
| c98962_g1 | 762106909 | glycogenin-1-like isoform X3 [*Crassostrea gigas*] | 11.76643 | 41.38333 | 13.02767 | 33.758 | 26.28933 | 13.902 |
| c86623_g1 | 762093198 | growth factor receptor-bound protein 2-like isoform X1 [*Crassostrea gigas*] | 0.953333 | 1.9823 | 1.989333 | 1.3583 | 3.45008 | 3.995833 |
| c101959_g1 | 405975684 | HEAT repeat-containing protein 7A [*Crassostrea gigas*] | 1.900333 | 0.327913 | 0.6645 | 1.718433 | 0.655433 | 2.398217 |
|  | 405952109 | Hemicentin-1 [*Crassostrea gigas*] | 6.0374 | 3.284087 | 3.999067 | 0.669097 | 7.7725 | 9.2586 |
|  | 405977312 | Heterogeneous nuclear ribonucleoprotein 27C [*Crassostrea gigas*] | 11.1331 | 11.25 | 5.354233 | 13.07867 | 9.282567 | 5.0157 |
|  | 405952153 | Heterogeneous nuclear ribonucleoprotein H [*Crassostrea gigas*] | 25.42167 | 9.247033 | 24.44533 | 24.124 | 19.491 | 19.294 |
|  | 405957027 | Heterogeneous nuclear ribonucleoprotein K [*Crassostrea gigas*] | 35.30633 | 13.88933 | 36.46633 | 38.33233 | 23.853 | 20.324 |
|  | 405959264 | Heterogeneous nuclear ribonucleoprotein U-like protein 1 [*Crassostrea gigas*] | 9.5399 | 3.971567 | 12.7375 | 8.966867 | 9.384867 | 16.20967 |
|  | 405978261 | Hexokinase type 2 [*Crassostrea gigas*] | 4.764033 | 2.31222 | 5.007433 | 15.67243 | 7.205633 | 5.642233 |
|  | 405962319 | Histone H3 [*Crassostrea gigas*] | 157.4067 | 113.4633 | 223.6467 | 121.6633 | 108.0853 | 306.9767 |
|  | 405969689 | Hydrocephalus-inducing-like protein [*Crassostrea gigas*] | 8.914767 | 4.620467 | 1.0084 | 0 | 6.4394 | 8.5587 |
|  | 405951837 | Hydroxyacyl-coenzyme A dehydrogenase, mitochondrial [*Crassostrea gigas*] | 35.60333 | 18.86033 | 24.41833 | 19.135 | 28.73833 | 34.66433 |
|  | 405963114 | Hydroxysteroid dehydrogenase-like protein 2 [*Crassostrea gigas*] | 1.9035 | 3.631787 | 2.361933 | 2.4551 | 0.358733 | 1.600933 |
|  | 405962051 | Importin subunit alpha-3 [*Crassostrea gigas*] | 5.4017 | 4.953333 | 9.411933 | 19.42 | 12.04643 | 14.664 |
|  | 405973144 | Importin subunit beta-1 [*Crassostrea gigas*] | 18.75633 | 4.627367 | 16.047 | 20.72007 | 20.957 | 30.063 |
|  | 405958012 | Importin-5 [*Crassostrea gigas*] | 3.177833 | 2.639167 | 4.3621 | 7.939467 | 7.196733 | 6.686467 |
|  | 405969882 | Importin-7 [*Crassostrea gigas*] | 1.272167 | 0 | 1.659167 | 1.7239 | 0.668747 | 6.355767 |
|  | 405950357 | Integrin alpha-6 [*Crassostrea gigas*] | 0.958133 | 15.21267 | 1.353567 | 6.897433 | 4.486367 | 2.346087 |
| c87788_g2 | 762102409 | integrin alpha-6-like isoform X2 [*Crassostrea gigas*] | 0 | 0 | 0 | 3.436867 | 0 | 0.320187 |
|  | 405950471 | Integrin alpha-8 [*Crassostrea gigas*] | 0 | 2.316187 | 0 | 0.9927 | 0.983133 | 0 |
|  | 15004986 | integrin beta cgh [*Crassostrea gigas*] | 18.43867 | 27.13133 | 19.07933 | 46.242 | 25.28367 | 12.5548 |
| c100530_g1 | 762106758 | integrin beta pat-3-like [*Crassostrea gigas*] | 0 | 7.9224 | 0 | 5.7586 | 6.833567 | 0.320187 |
| c101066_g1 | 762162532 | integrin beta-1-B-like [*Crassostrea gigas*] | 9.8555 | 13.88333 | 11.0263 | 28.663 | 13.012 | 3.971433 |
| c95812_g1 | 762147513 | interferon-induced protein 44-like [*Crassostrea gigas*] | 2.226553 | 1.64452 | 3.686767 | 5.1882 | 2.369367 | 2.039767 |
|  | 405973588 | Kielin/chordin-like protein [*Crassostrea gigas*] | 34.97767 | 89.671 | 35.48533 | 36.87133 | 47.78167 | 34.156 |
|  | 405975838 | kinase C and casein kinase substrate in neurons protein 2 [*Crassostrea gigas*] | 6.993233 | 10.58047 | 9.039267 | 14.921 | 8.224233 | 5.322033 |
|  | 405977054 | Kinesin heavy chain [*Crassostrea gigas*] | 4.131167 | 9.595467 | 4.0211 | 9.719933 | 9.995867 | 4.9181 |
|  | 405972492 | Kinesin-related protein 1 [*Crassostrea gigas*] | 10.16833 | 0 | 8.078 | 2.014633 | 2.032813 | 4.598567 |
| c103140_g1 | 762101975 | laccase-4-like [*Crassostrea gigas*] | 2.538 | 4.2896 | 0 | 2.367467 | 2.869933 | 1.378333 |
| c101403_g2 | 405958866 | Lachesin [*Crassostrea gigas*] | 5.0887 | 0 | 2.9923 | 1.773233 | 0 | 1.921133 |
| c156519_g1 | 762121736 | lactadherin-like isoform X2 [*Crassostrea gigas*] | 0.950167 | 4.6363 | 2.0222 | 2.422233 | 2.364933 | 0 |
|  | 405969732 | Laminin subunit alpha [*Crassostrea gigas*] | 40.983 | 6.2719 | 24.09867 | 70.76833 | 27.80833 | 8.635967 |
| c103776_g1 | 762109423 | laminin subunit alpha-like [*Crassostrea gigas*] | 36.22367 | 6.2719 | 19.75567 | 57.09367 | 27.44067 | 7.003333 |
|  | 405963229 | Laminin subunit gamma-1 [*Crassostrea gigas*] | 12.398 | 0 | 1.674167 | 19.97567 | 0.699767 | 2.053617 |
|  | 405950801 | La-related protein 1 [*Crassostrea gigas*] | 5.4065 | 3.306833 | 7.702567 | 13.424 | 9.654867 | 4.622333 |
|  | 405952168 | La-related protein 4 [*Crassostrea gigas*] | 0.953333 | 1.979367 | 0 | 3.765933 | 2.799033 | 0 |
| c99318_g1 | 762141840 | lethal(2) giant larvae protein homolog 2-like isoform X1 [*Crassostrea gigas*] | 12.73017 | 8.277 | 16.80067 | 24.93733 | 21.99333 | 14.64 |
|  | 405952027 | Lethal(2) giant larvae-like protein 1 [*Crassostrea gigas*] | 12.09483 | 8.277 | 16.80067 | 22.199 | 20.29267 | 13.99967 |
| c96717_g1 | 762130693 | leucine-rich repeat and death domain-containing protein 1-like [*Crassostrea gigas*] | 11.43803 | 8.256233 | 12.3647 | 15.21733 | 14.41567 | 12.252 |
|  | 405952986 | Leucine-rich repeat-containing G-protein coupled receptor 6 [*Crassostrea gigas*] | 12.7141 | 3.633767 | 8.7038 | 13.10033 | 9.508767 | 6.9512 |
|  | 405962612 | Leucine-rich repeats and immunoglobulin-like domains protein 3 [*Crassostrea gigas*] | 13.983 | 8.5891 | 8.672633 | 21.75667 | 17.12167 | 11.14523 |
|  | 405959794 | Leucine-zipper-like transcriptional regulator 1 [*Crassostrea gigas*] | 10.8194 | 19.523 | 9.3683 | 18.89367 | 13.30383 | 6.627167 |
| c99032_g1 | 405971834 | Leukocyte elastase inhibitor [*Crassostrea gigas*] | 5.724833 | 10.59033 | 3.686767 | 10.63047 | 6.869033 | 8.962567 |
|  | 405967541 | LIM and SH3 domain protein Lasp [*Crassostrea gigas*] | 11.12743 | 15.19967 | 5.003267 | 6.8298 | 9.8187 | 5.224467 |
| c95819_g1 | 405977265 | Long-chain specific acyl-CoA dehydrogenase, mitochondrial [*Crassostrea gigas*] | 3.8176 | 11.5788 | 4.001967 | 3.676433 | 2.378233 | 6.000667 |
|  | 405952151 | Long-chain-fatty-acid--CoA ligase 1 [*Crassostrea gigas*] | 13.34633 | 2.643067 | 12.04 | 16.038 | 19.55767 | 10.96783 |
|  | 405952731 | Lupus La-like protein [*Crassostrea gigas*] | 2.861633 | 12.89357 | 1.004233 | 17.20433 | 9.583767 | 1.009353 |
| c102518_g1 | 762090156 | lysosomal alpha-glucosidase-like [*Crassostrea gigas*] | 4.765133 | 2.314177 | 3.0252 | 4.138833 | 4.845133 | 4.977433 |
| c99479_g1 | 762118717 | lysosome-associated membrane glycoprotein 1-like isoform X2 [*Crassostrea gigas*] | 2.543267 | 11.91353 | 2.687967 | 10.54833 | 5.119667 | 2.6524 |
|  | 405965903 | Major egg antigen [*Crassostrea gigas*] | 0 | 1.979367 | 0 | 3.524533 | 0.983133 | 1.305133 |
|  | 405965621 | Mammalian ependymin-related protein 1, partial [*Crassostrea gigas*] | 21.62867 | 40.70233 | 14.36733 | 37.23367 | 28.54767 | 29.51633 |
| c86491_g1 | 762157957 | mechanosensory protein 2-like isoform X3 [*Crassostrea gigas*] | 22.88867 | 5.297033 | 14.72933 | 15.21733 | 11.01903 | 15.21733 |
|  | 405959230 | Membrane metallo-endopeptidase-like 1 [*Crassostrea gigas*] | 5.7207 | 3.291033 | 11.70393 | 30.31033 | 7.542167 | 12.58267 |
|  | 405953527 | Mesenchyme-specific cell surface glycoprotein [*Crassostrea gigas*] | 14.316 | 5.959833 | 9.7098 | 5.835333 | 9.2208 | 7.647033 |
|  | 405974809 | Metabotropic glutamate receptor 3 [*Crassostrea gigas*] | 32.75933 | 0.327913 | 18.07467 | 36.67733 | 3.330333 | 15.855 |
|  | 405968797 | Methenyltetrahydrofolate synthetase domain-containing protein [*Crassostrea gigas*] | 3.816533 | 0.327913 | 4.345867 | 3.773233 | 1.740533 | 2.33222 |
|  | 405972616 | Methylcrotonoyl-CoA carboxylase subunit alpha, mitochondrial [*Crassostrea gigas*] | 1.27591 | 11.90987 | 5.013333 | 3.436867 | 5.757367 | 4.667767 |
| c93709_g2 | 762164091 | MICOS complex subunit Mic60-like isoform X1 [*Crassostrea gigas*] | 5.085 | 3.305867 | 11.38533 | 5.422267 | 2.364933 | 6.6133 |
|  | 405954434 | Microtubule-associated protein RP/EB family member 3 [*Crassostrea gigas*] | 3.181 | 5.614067 | 4.016933 | 9.5411 | 6.7805 | 3.626833 |
| c78564_g1 | 405973457 | minus strand | 0.319373 | 0 | 0.339733 | 0.3382 | 3.759967 | 2.6419 |
| c85400_g1 | 406817026 | minus strand | 0.952807 | 2.644087 | 3.6922 | 6.5465 | 3.0514 | 1.326187 |
| c93465_g1 | 405971145 | minus strand | 104.2957 | 35.39533 | 100.094 | 59.642 | 62.91067 | 98.683 |
| c95340_g1 | 762099550 | minus strand | 46.755 | 10.24747 | 35.43567 | 26.80367 | 20.45667 | 43.704 |
| c98275_g1 | 665815290 | minus strand | 7.306267 | 1.3275 | 7.712133 | 4.104133 | 4.158633 | 11.7233 |
| c93185_g1 | 405950468 | minus strand | 0 | 5.279267 | 0 | 11.5683 | 4.1099 | 0 |
| c94932_g1 | 676458648 | minus strand | 10.80503 | 31.101 | 11.72383 | 22.41267 | 20.585 | 11.66387 |
| c95118_g1 | 762168992 | minus strand | 0.95651 | 7.6142 | 0.674067 | 1.3309 | 5.3853 | 0.320187 |
|  | 405960381 | Monocarboxylate transporter 12 [*Crassostrea gigas*] | 6.680267 | 12.23643 | 3.0127 | 5.153467 | 7.878767 | 6.296433 |
|  | 405970698 | Multidrug resistance protein 1 [*Crassostrea gigas*] | 0 | 0 | 0 | 12.4313 | 2.3871 | 2.283433 |
|  | 405956360 | Multidrug resistance protein 1, partial [*Crassostrea gigas*] | 4.459043 | 0.6657 | 1.698733 | 29.67567 | 2.745833 | 2.645733 |
| c98973_g1 | 762109068 | multidrug resistance-associated protein 1-like isoform X1 [*Crassostrea gigas*] | 4.1354 | 0 | 1.674167 | 1.0694 | 0.341033 | 2.25185 |
|  | 405975739 | Murinoglobulin-2 [*Crassostrea gigas*] | 8.268667 | 17.57047 | 6.350167 | 16.678 | 19.934 | 7.302467 |
|  | **405972818** | **Myosin heavy chain, striated muscle [*Crassostrea gigas*]** | **1812.367** | **795.0433** | **1831.2** | **1014.637** | **1211.033** | **1783.167** |
|  | **405975056** | **Myosin heavy chain, striated muscle [*Crassostrea gigas*]** | **455.6767** | **178.03** | **477.1067** | **309.3033** | **295.3333** | **441.7133** |
|  | 405961702 | Myosin regulatory light chain A, smooth adductor muscle [*Crassostrea gigas*] | 43.256 | 114.1467 | 56.89133 | 116.6567 | 81.746 | 97.11733 |
|  | 405974721 | Na(+)/H(+) exchange regulatory cofactor NHE-RF1 [*Crassostrea gigas*] | 62.33467 | 122.7333 | 70.90033 | 99.11167 | 75.89133 | 55.48567 |
|  | 405975835 | NAD(P) transhydrogenase, mitochondrial [*Crassostrea gigas*] | 87.434 | 9.934367 | 44.18067 | 45.085 | 21.89133 | 47.247 |
|  | 405969458 | NADH dehydrogenase [ubiquinone] flavoprotein 2, mitochondrial, partial [*Crassostrea gigas*] | 1.26954 | 6.946467 | 7.033833 | 11.8716 | 8.9372 | 11.53867 |
|  | 405965726 | NADP-dependent malic enzyme [*Crassostrea gigas*] | 31.792 | 44.675 | 45.83433 | 17.69967 | 46.356 | 64.98533 |
| c91009_g1 | 762070002 | NADPH--cytochrome P450 reductase-like [*Crassostrea gigas*] | 2.5518 | 0 | 5.671933 | 0 | 1.023067 | 4.003 |
|  | 405967527 | Nesprin-1 [*Crassostrea gigas*] | 0.63715 | 3.944833 | 0.6795 | 4.795167 | 11.32437 | 3.4181 |
| c101304_g1 | 762085332 | neural cell adhesion molecule 2-like isoform X7 [*Crassostrea gigas*] | 66.12567 | 152.24 | 69.87033 | 119.3233 | 109.6767 | 48.66367 |
| c101658_g2 | 762095530 | neural-cadherin-like [*Crassostrea gigas*] | 3.498243 | 14.87967 | 0 | 6.9742 | 2.781333 | 0 |
|  | 405964679 | Neurexin-4 [*Crassostrea gigas*] | 1.2775 | 2.327033 | 0 | 5.146167 | 0 | 1.9843 |
|  | 405963373 | Neurogenic locus Notch protein [*Crassostrea gigas*] | 1.9083 | 13.894 | 0 | 4.7659 | 4.2118 | 0 |
|  | 405958312 | Neuroglian [*Crassostrea gigas*] | 6.3609 | 22.50267 | 4.0307 | 26.316 | 10.55817 | 3.947033 |
|  | 405960111 | Neuroglian [*Crassostrea gigas*] | 0 | 0 | 2.0289 | 0.3382 | 0 | 1.336733 |
| c102881_g1 | 762129353 | neuroglian-like isoform X1 [*Crassostrea gigas*] | 4.776267 | 0 | 4.008633 | 2.765933 | 2.4137 | 5.994 |
| c88012_g1 | 762111301 | Neuronal acetylcholine receptor subunit alpha-10 [*Crassostrea gigas*] | 1.59263 | 0 | 1.6879 | 0 | 0.358733 | 0.344567 |
| c96718_g1 | 762143197 | neutral ceramidase-like [*Crassostrea gigas*] | 1.590473 | 3.304867 | 0 | 6.111433 | 3.423433 | 1.35392 |
| c94770_g1 | 762089386 | nidogen-1-like isoform X1 [*Crassostrea gigas*] | 3.816533 | 0.983733 | 2.344067 | 8.546467 | 3.835367 | 1.70905 |
| c58989_g1 | 762081924 | nidogen-2-like [*Crassostrea gigas*] | 42.92233 | 61.855 | 42.85733 | 44.518 | 56.72367 | 23.64867 |
| c99574_g1 | 762071868 | ornithine aminotransferase, mitochondrial-like [*Crassostrea gigas*] | 26.38533 | 34.43133 | 28.77633 | 18.55 | 32.308 | 37.11133 |
|  | 405951454 | Outer dense fiber protein 3 [*Crassostrea gigas*] | 37.833 | 42.37733 | 30.72567 | 1.718433 | 38.455 | 33.52633 |
|  | 405952329 | PAB-dependent poly(A)-specific ribonuclease subunit 2 [*Crassostrea gigas*] | 1.9083 | 3.9696 | 1.698733 | 0.9927 | 3.3924 | 0 |
|  | 405958470 | Papilin [*Crassostrea gigas*] | 51.50867 | 16.545 | 15.37867 | 23.39667 | 10.92113 | 4.622333 |
|  | 405966986 | Paramyosin [*Crassostrea gigas*] | 738.1867 | 92.45267 | 355.5433 | 320.72 | 100.324 | 266.8033 |
| c94503_g2 | 762070443 | pathogen-related protein-like [*Crassostrea gigas*] | 0 | 0.6657 | 0 | 4.919397 | 0 | 0.320187 |
|  | 405960428 | PDZ and LIM domain protein 1 [*Crassostrea gigas*] | 8.2713 | 16.22 | 6.0099 | 8.076733 | 2.75028 | 3.275033 |
|  | 405970466 | Periostin [*Crassostrea gigas*] | 54.67467 | 58.879 | 64.96167 | 178.8067 | 88.63733 | 47.84267 |
|  | 405977917 | Peroxisomal multifunctional enzyme type 2 [*Crassostrea gigas*] | 0 | 0 | 0 | 0 | 0.655433 | 2.0187 |
| c93516_g1 | 762084138 | peroxisomal multifunctional enzyme type 2-like [*Crassostrea gigas*] | 0 | 0 | 0 | 0 | 0.655433 | 2.732253 |
| c87836_g1 | 762127059 | Peroxisomal NADH pyrophosphatase NUDT12 [*Crassostrea gigas*] | 0 | 7.583433 | 3.353733 | 3.800633 | 7.768133 | 4.987967 |
| c90267_g1 | 762070867 | phosphoenolpyruvate carboxykinase, cytosolic [GTP]-like isoform X1 [*Crassostrea gigas*] | 1.5937 | 1.651467 | 2.009733 | 2.111433 | 1.434967 | 5.335867 |
| c80872_g1 | 762086921 | phosphoenolpyruvate phosphomutase [*Crassostrea gigas*] | 5.4012 | 4.960233 | 7.3843 | 3.435033 | 6.917667 | 6.0356 |
|  | 405950592 | Phosphoglucomutase-1 [*Crassostrea gigas*] | 10.17217 | 11.5888 | 4.007367 | 4.118767 | 5.7972 | 6.8774 |
| c93257_g1 | 762136319 | phosphomannomutase-like isoform X1 [*Crassostrea gigas*] | 1.913067 | 3.302933 | 2.003033 | 4.138833 | 3.6802 | 1.600933 |
| c97751_g1 | 762070302 | piwi-like protein 1 isoform X1 [*Crassostrea gigas*] | 14.632 | 15.85957 | 19.04233 | 8.284967 | 17.12167 | 24.13133 |
|  | 405969430 | Plasma alpha-L-fucosidase [*Crassostrea gigas*] | 0.958133 | 0 | 3.003133 | 4.787867 | 2.054933 | 2.996967 |
|  | 405978156 | Plasma membrane calcium-transporting ATPase 3 [*Crassostrea gigas*] | 43.23833 | 5.9292 | 36.17033 | 31.22233 | 25.99233 | 24.233 |
| c83148_g1 | 762111593 | plasminogen activator inhibitor 1 RNA-binding protein-like isoform X1 [*Crassostrea gigas*] | 7.307867 | 7.9303 | 16.03367 | 27.553 | 18.831 | 8.245833 |
|  | 405958107 | Poly [ADP-ribose] polymerase 1 [*Crassostrea gigas*] | 2.5444 | 0 | 1.671633 | 0 | 1.023067 | 0.971083 |
|  | 405975722 | Polyamine-modulated factor 1-binding protein 1 [*Crassostrea gigas*] | 0 | 28.46267 | 0 | 1.3236 | 5.221567 | 1.009353 |
| c95528_g1 | 762121240 | PREDICTED: acetyl-CoA acetyltransferase, mitochondrial-like [*Crassostrea gigas*] | 6.994867 | 5.287167 | 6.677433 | 3.442333 | 5.757367 | 11.33003 |
| c101643_g2 | 762115946 | pregnancy zone protein-like [*Crassostrea gigas*] | 12.0885 | 28.15567 | 10.69197 | 23.76367 | 21.08533 | 19.21667 |
|  | 405952227 | Pre-mRNA-processing factor 39 [*Crassostrea gigas*] | 2.861633 | 7.2764 | 5.354233 | 12.62317 | 6.877933 | 6.021733 |
|  | 405965891 | Prenylcysteine oxidase [*Crassostrea gigas*] | 0.958133 | 0 | 0.9905 | 6.9742 | 0 | 0.640367 |
| c81481_g1 | 762145153 | probable ATP synthase subunit g 1, mitochondrial [*Crassostrea gigas*] | 12.082 | 7.9421 | 5.6911 | 8.153367 | 7.825733 | 8.914033 |
|  | 405965826 | Programmed cell death protein 4 [*Crassostrea gigas*] | 0 | 0.983733 | 3.6772 | 5.160767 | 6.1427 | 6.641033 |
|  | 405968717 | Programmed cell death protein 6 [*Crassostrea gigas*] | 7.305233 | 0 | 7.394133 | 3.802467 | 2.333933 | 5.959067 |
| c79816_g1 | 762091059 | prohibitin-2-like [*Crassostrea gigas*] | 14.943 | 9.2774 | 22.09167 | 13.74033 | 16.165 | 18.88233 |
| c90524_g1 | 762125425 | proliferating cell nuclear antigen-like [*Crassostrea gigas*] | 6.036233 | 4.630333 | 5.007433 | 8.615867 | 6.815867 | 3.0735 |
|  | 405978690 | Propionyl-CoA carboxylase beta chain, mitochondrial [*Crassostrea gigas*] | 12.08633 | 10.25437 | 16.709 | 16.59 | 22.206 | 22.83367 |
| c94819_g1 | 762104009 | prostaglandin reductase 1-like [*Crassostrea gigas*] | 3.496133 | 1.979367 | 3.6826 | 4.131533 | 6.169267 | 6.905767 |
|  | 405960660 | Protein disulfide-isomerase A5 [*Crassostrea gigas*] | 3.490267 | 0.332853 | 0.668667 | 4.904767 | 2.4137 | 3.250667 |
| c91045_g2 | 762163372 | protein disulfide-isomerase A5-like [*Crassostrea gigas*] | 0.633433 | 0.332853 | 0.668667 | 6.5958 | 1.337467 | 2.6103 |
| c93720_g1 | 762125371 | protein disulfide-isomerase-like [*Crassostrea gigas*] | 112.5467 | 216.0467 | 127.8567 | 280.79 | 180.4767 | 144.61 |
|  | 405965843 | Protein ERGIC-53 [*Crassostrea gigas*] | 7.623667 | 0 | 4.022367 | 7.5412 | 1.364067 | 2.6419 |
|  | 405962525 | Protein FAM63B [*Crassostrea gigas*] | 0 | 1.967467 | 0 | 5.4624 | 2.0904 | 0 |
|  | 405969398 | Protein jagged-2 [*Crassostrea gigas*] | 0 | 0 | 0 | 0.3656 | 0.983133 | 0.650903 |
| c78719_g1 | 762131783 | protein kinase C and casein kinase substrate in neurons protein 1-like isoform X1 [*Crassostrea gigas*] | 6.993233 | 12.2198 | 9.039267 | 20.05233 | 9.207467 | 5.322033 |
|  | 405965662 | Protein lap4 [*Crassostrea gigas*] | 0 | 6.2838 | 2.6921 | 5.263067 | 0 | 0.344567 |
|  | 405972778 | Protein phosphatase 1B [*Crassostrea gigas*] | 15.57767 | 5.289133 | 9.682133 | 11.91923 | 14.33167 | 10.56037 |
|  | 405972892 | Protein SPATIAL [*Crassostrea gigas*] | 39.416 | 34.76633 | 34.83133 | 3.056667 | 38.94233 | 33.14633 |
|  | 405975243 | Protein unc-87 [*Crassostrea gigas*] | 12.085 | 16.21933 | 5.340133 | 10.8004 | 9.858433 | 7.9534 |
|  | 405963677 | Protein VPRBP [*Crassostrea gigas*] | 9.217133 | 55.599 | 13.71967 | 35.747 | 29.08833 | 13.49457 |
|  | 405950809 | Protocadherin Fat 4 [*Crassostrea gigas*] | 99.811 | 435.7333 | 136.2233 | 384.35 | 287.4667 | 91.67867 |
|  | 405962160 | Protocadherin Fat 4 [*Crassostrea gigas*] | 4.452633 | 0 | 3.696367 | 1.014633 | 0.717467 | 4.3648 |
| c50441_g1 | 405953857 | protocadherin Fat 4-like [*Crassostrea gigas*] | 2.861633 | 6.6107 | 4.016933 | 7.559233 | 4.778633 | 3.651267 |
| c48957_g1 | 405951929 | protocadherin Fat 4-like [*Crassostrea gigas*] | 1.914643 | 19.858 | 2.038467 | 13.6431 | 7.382767 | 5.934667 |
| c115430_g1 | 762134719 | protocadherin Fat 4-like [*Crassostrea gigas*] | 2.542233 | 9.590533 | 6.680333 | 12.698 | 9.605933 | 3.317167 |
| c101457_g1 | 762080804 | protocadherin Fat 4-like isoform X3 [*Crassostrea gigas*] | 70.58267 | 211.75 | 75.62167 | 195.6033 | 138.6533 | 57.02433 |
|  | 405951930 | Protocadherin-like wing polarity protein stan [*Crassostrea gigas*] | 0.319373 | 4.302433 | 0 | 4.7312 | 3.033667 | 1.35392 |
| c100837_g1 | 762145751 | puromycin-sensitive aminopeptidase-like [*Crassostrea gigas*] | 16.219 | 15.53267 | 20.74367 | 8.976 | 15.678 | 22.645 |
|  | 405951163 | Putative adenylate kinase-like protein C9orf98-like protein [*Crassostrea gigas*] | 5.726967 | 5.2921 | 10.0681 | 0 | 10.3456 | 10.96417 |
|  | 405968899 | Putative beta-D-xylosidase 5 [*Crassostrea gigas*] | 8.2681 | 26.81533 | 12.76333 | 21.672 | 16.78067 | 10.63717 |
| c96793_g1 | 405970391 | putative per-hexamer repeat protein 5 isoform X9 [*Crassostrea gigas*] | 6.993833 | 16.87 | 3.0098 | 6.029167 | 8.011667 | 4.9076 |
|  | 405961471 | Putative serine carboxypeptidase CPVL [*Crassostrea gigas*] | 22.26633 | 11.90267 | 19.41633 | 16.61033 | 19.159 | 21.093 |
|  | 405978194 | Putative sulfite oxidase, mitochondrial [*Crassostrea gigas*] | 3.4998 | 2.648053 | 5.355533 | 12.25023 | 3.46778 | 1.6536 |
|  | 405957461 | Putative thiopurine S-methyltransferase [*Crassostrea gigas*] | 0.31672 | 1.6475 | 0.330167 | 3.442333 | 1.3818 | 0.984933 |
|  | 405970353 | Replication protein A 70 kDa DNA-binding subunit [*Crassostrea gigas*] | 1.272167 | 1.995167 | 0.334333 | 4.511767 | 2.816767 | 0 |
|  | 405963940 | Reticulon-4 [*Crassostrea gigas*] | 3.810733 | 2.644087 | 4.012767 | 4.469733 | 4.171933 | 5.001867 |
|  | 405972837 | Retinal dehydrogenase 1 [*Crassostrea gigas*] | 9.850567 | 17.21567 | 13.403 | 5.8847 | 20.46533 | 15.684 |
|  | 405953430 | Ribosome-binding protein 1 [*Crassostrea gigas*] | 4.136467 | 32.75233 | 3.6772 | 14.18967 | 13.07833 | 9.2827 |
|  | 405957186 | RNA-binding protein Nova-1 [*Crassostrea gigas*] | 8.900567 | 12.56267 | 6.6995 | 14.78233 | 10.2615 | 7.622667 |
| c103590_g1 | 762100293 | rootletin-like isoform X6 [*Crassostrea gigas*] | 35.61967 | 290.88 | 51.55667 | 42.475 | 141.2233 | 73.693 |
|  | 405962126 | rRNA 2'-O-methyltransferase fibrillarin [*Crassostrea gigas*] | 0 | 0.331877 | 0 | 0 | 0 | 3.0247 |
| **c96209_g1** | **307197748** | **Ryanodine receptor 44F [Harpegnathos saltator]** | **1.905633** | **0** | **2.027633** | **0** | **1.023067** | **4.2256** |
| c80501_g1 | 405963560 | Sarcoplasmic calcium-binding protein [*Crassostrea gigas*] | 0 | 0.997577 | 3.996533 | 3.104133 | 2.0904 | 7.201467 |
|  | 405967048 | Scaffold attachment factor B1 [*Crassostrea gigas*] | 0 | 0.995633 | 0 | 0 | 1.04518 | 0 |
| c146090_g1 | 443687279 | scavenger receptor cysteine-rich domain-containing protein SCART1-like [Sarcophilus harrisii] | 12.39 | 34.72367 | 8.6776 | 20.66667 | 12.98513 | 10.27177 |
| c100128_g1 | 762155471 | scavenger receptor cysteine-rich type 1 protein M130-like [*Crassostrea gigas*] | 0 | 7.932233 | 0 | 4.462433 | 3.410167 | 0 |
|  | 405972994 | SEC13-like protein [*Crassostrea gigas*] | 0 | 0.99662 | 0.339733 | 3.084033 | 1.3818 | 0.98162 |
|  | 405951043 | Serine protease inhibitor dipetalogastin [*Crassostrea gigas*] | 8.5911 | 10.25833 | 9.687567 | 8.5393 | 10.09757 | 0.992167 |
|  | 405964168 | Serine/threonine-protein phosphatase 2B catalytic subunit alpha isoform [*Crassostrea gigas*] | 4.4505 | 11.5375 | 8.7091 | 2.7166 | 11.65217 | 10.66163 |
|  | 405954824 | Severin [*Crassostrea gigas*] | 113.52 | 262.48 | 111.9033 | 151.8633 | 137.34 | 90.32467 |
|  | 405973087 | SH3 domain-binding glutamic acid-rich protein [*Crassostrea gigas*] | 4.140667 | 0.663767 | 0 | 6.5739 | 0.358733 | 10.5426 |
|  | 405953442 | Signal peptide, CUB and EGF-like domain-containing protein 1 [*Crassostrea gigas*] | 35.60533 | 79.73367 | 32.75767 | 79.19933 | 47.50767 | 23.43633 |
| **c81197_g1** | **762116864** | **sodium/potassium-transporting ATPase subunit alpha-like [*Crassostrea gigas*]** | **242.91** | **114.1433** | **218.14** | **289.0433** | **169.3633** | **184.1167** |
|  | 405965139 | Sorting nexin-2 [*Crassostrea gigas*] | 0 | 1.3196 | 1.3373 | 3.111433 | 3.095733 | 2.2901 |
|  | 405961240 | Sperm surface protein Sp17 [*Crassostrea gigas*] | 4.773633 | 11.247 | 5.003267 | 4.862767 | 8.596167 | 7.291967 |
|  | 405969074 | Spermatogenesis-associated protein 18-like protein [*Crassostrea gigas*] | 56.26833 | 40.04767 | 65.281 | 32.09067 | 59.26567 | 80.17167 |
|  | 405970223 | Splicing factor 3B subunit 3 [*Crassostrea gigas*] | 3.180433 | 3.313767 | 6.0553 | 13.824 | 9.131967 | 4.730453 |
|  | 405976087 | Splicing factor U2AF 50 kDa subunit [*Crassostrea gigas*] | 2.227667 | 0 | 2.667533 | 4.753 | 0.983133 | 4.027387 |
|  | 405955028 | Steroid 17-alpha-hydroxylase/17,20 lyase [*Crassostrea gigas*] | 3.181 | 0 | 7.039733 | 0.3382 | 1.731667 | 9.645167 |
|  | 405968979 | Steroid 17-alpha-hydroxylase/17,20 lyase [*Crassostrea gigas*] | 0 | 0 | 2.662133 | 0 | 1.076233 | 3.661767 |
| c94466_g1 | 762104107 | stress-induced-phosphoprotein 1-like [*Crassostrea gigas*] | 1.27591 | 5.309933 | 3.0127 | 7.926633 | 6.4881 | 7.6193 |
|  | 405959519 | Succinate-semialdehyde dehydrogenase, mitochondrial [*Crassostrea gigas*] | 17.49 | 11.24987 | 23.097 | 8.601267 | 16.98867 | 20.33733 |
|  | 405963331 | Succinyl-CoA ligase [ADP-forming] subunit beta, mitochondrial [*Crassostrea gigas*] | 13.67833 | 3.970567 | 21.767 | 11.17503 | 23.54767 | 24.104 |
|  | 405971538 | Synaptophysin [*Crassostrea gigas*] | 3.812833 | 2.64211 | 3.342867 | 6.559233 | 4.774133 | 2.33222 |
| c99665_g2 | 405965400 | Synaptopodin-2 [*Crassostrea gigas*] | 8.274867 | 7.947067 | 5.307633 | 2.3583 | 7.745767 | 6.992833 |
|  | 405950905 | Syntaxin-5 [*Crassostrea gigas*] | 10.80737 | 6.953433 | 6.3813 | 13.2775 | 6.696267 | 10.6547 |
| c97171_g1 | 762108542 | T-complex protein 1 subunit beta-like [*Crassostrea gigas*] | 8.896767 | 19.842 | 17.77067 | 34.43067 | 27.39167 | 22.566 |
| c91251_g1 | 405977961 | T-complex protein 1 subunit delta, partial [*Crassostrea gigas*] | 41.33667 | 21.861 | 43.856 | 26.22067 | 35.842 | 53.31033 |
|  | 405953549 | T-complex protein 1 subunit zeta [*Crassostrea gigas*] | 5.396 | 7.275433 | 5.024067 | 2.0895 | 8.7999 | 7.264233 |
|  | 405952896 | Tectonic-3 [*Crassostrea gigas*] | 0.952287 | 6.9326 | 0.660333 | 2.449633 | 0.996467 | 0 |
| c80732_g1 | 762169708 | tektin-2-like [*Crassostrea gigas*] | 4.135967 | 50.63967 | 15.04467 | 6.966867 | 41.47933 | 32.97933 |
|  | 405975469 | Tektin-3 [*Crassostrea gigas*] | 110.6833 | 106.18 | 119.4733 | 14.68913 | 124.0267 | 142.0067 |
| c89247_g1 | 762135468 | tektin-3-like isoform X2 [*Crassostrea gigas*] | 198.7167 | 107.8767 | 161.0767 | 23.738 | 149.4733 | 176.4467 |
|  | 405960104 | Tenascin-X [*Crassostrea gigas*] | 0.317777 | 1.652453 | 0.668667 | 2.52453 | 3.113467 | 0.344567 |
|  | 405972180 | Tetratricopeptide repeat protein 25 [*Crassostrea gigas*] | 0.955453 | 5.611153 | 0 | 1.042033 | 3.698 | 1.35392 |
|  | 338815381 | thioredoxin [Crassostrea ariakensis] | 1.2669 | 1.649433 | 7.694167 | 13.41667 | 10.2391 | 8.847833 |
|  | 405969114 | Thioredoxin domain-containing protein 3-like protein [*Crassostrea gigas*] | 11.12867 | 39.71967 | 22.14667 | 24.773 | 35.253 | 31.327 |
|  | 405970435 | Thioredoxin domain-containing protein 5 [*Crassostrea gigas*] | 2.5444 | 2.9789 | 2.329067 | 5.870067 | 0 | 5.297633 |
| c98785_g1 | 762080959 | thioredoxin reductase 1, cytoplasmic-like [*Crassostrea gigas*] | 0.635567 | 1.321533 | 6.9937 | 3.400333 | 6.479433 | 6.436067 |
| c87811_g1 | 762139008 | thioredoxin-1-like [*Crassostrea gigas*] | 3.17304 | 4.298467 | 6.6995 | 12.65043 | 10.55817 | 8.875567 |
| c96033_g1 | 762085768 | thioredoxin-like protein 1 [*Crassostrea gigas*] | 2.856833 | 8.9397 | 3.018133 | 15.12033 | 5.124033 | 2.9238 |
| c85190_g1 | 762162716 | THO complex subunit 4-A [*Crassostrea gigas*] | 14.948 | 2.976933 | 14.04133 | 16.47333 | 9.362467 | 11.30203 |
|  | 405974168 | Titin [*Crassostrea gigas*] | 60.74233 | 12.53413 | 63.33167 | 17.70533 | 38.99533 | 92.00567 |
|  | 405970417 | Titin [*Crassostrea gigas*] | 2.861633 | 16.22 | 3.6826 | 7.946767 | 11.71433 | 0.330717 |
|  | 405952729 | Titin [*Crassostrea gigas*] | 3.1794 | 6.275867 | 3.0127 | 3.793333 | 6.784833 | 4.340433 |
|  | 405970995 | Transcription factor BTF3-like protein 4 [*Crassostrea gigas*] | 27.02233 | 48.63833 | 26.78267 | 61.198 | 46.87 | 37.853 |
|  | 405960569 | Transforming growth factor-beta-induced protein ig-h3 [*Crassostrea gigas*] | 15.26433 | 36.394 | 29.79433 | 43.465 | 39.04367 | 42.472 |
| c90531_g1 | 762118303 | transforming growth factor-beta-induced protein ig-h3-like [*Crassostrea gigas*] | 17.16377 | 17.87167 | 15.383 | 44.60633 | 19.566 | 7.323533 |
| c89492_g1 | 762092088 | transforming growth factor-beta-induced protein ig-h3-like [*Crassostrea gigas*] | 20.982 | 43.01767 | 35.126 | 57.931 | 52.99033 | 43.14333 |
|  | 405967947 | Transgelin-2 [*Crassostrea gigas*] | 17.172 | 23.48167 | 11.7136 | 26.39067 | 21.08533 | 14.12767 |
|  | 405976865 | Translocon-associated protein subunit alpha [*Crassostrea gigas*] | 10.48667 | 7.2764 | 7.699567 | 15.56833 | 6.483867 | 1.312353 |
|  | 405966687 | Transmembrane emp24 domain-containing protein 1 [*Crassostrea gigas*] | 7.634667 | 3.303833 | 5.359667 | 7.677967 | 7.0861 | 4.3127 |
|  | 405950001 | Transmembrane emp24 domain-containing protein 2 [*Crassostrea gigas*] | 3.8118 | 0 | 4.351267 | 2.738533 | 3.441167 | 4.632867 |
|  | 405966231 | Transmembrane protein 2 [*Crassostrea gigas*] | 3.8155 | 4.965167 | 6.347267 | 2.10413 | 8.888533 | 6.683167 |
|  | 405957915 | Transmembrane protein 2 [*Crassostrea gigas*] | 0.953333 | 4.622433 | 3.026433 | 5.166267 | 0.655433 | 4.6467 |
|  | 405973883 | transport protein Sec24C [*Crassostrea gigas*] | 1.5889 | 0.988687 | 4.360867 | 11.10027 | 6.4128 | 5.0717 |
|  | 219806594 | tropomyosin [*Crassostrea gigas*] | 43.25 | 388.4833 | 105.8223 | 151.7867 | 218.4967 | 48.9 |
|  | 405967637 | Tropomyosin [*Crassostrea gigas*] | 21.62333 | 330.3767 | 41.18367 | 50.981 | 96.12467 | 15.88967 |
|  | 375073719 | tropomyosin 1, partial [Ostrea edulis] | 0 | 23.81633 | 5.696533 | 6.862733 | 16.515 | 3.661767 |
| c83385_g1 | 762145981 | trypsin-1-like [*Crassostrea gigas*] | 2.222333 | 0 | 2.988133 | 1.4624 | 2.0239 | 2.7012 |
|  | 405964567 | Tubulin beta chain [*Crassostrea gigas*] | 2732.133 | 1453.167 | 2455.233 | 1142.667 | 1949.933 | 2211.967 |
|  | 333449475 | tubulin beta-2B chain-like protein, partial [Crassostrea ariakensis] | 256.0033 | 195.5667 | 199.08 | 131.91 | 193.9167 | 168.6567 |
|  | 405969356 | Tudor domain-containing protein 1 [*Crassostrea gigas*] | 0 | 7.9214 | 0 | 0 | 2.063833 | 0 |
|  | 405976987 | Tyrosine-protein phosphatase Lar [*Crassostrea gigas*] | 0.958133 | 0.998567 | 0 | 18.914 | 2.723667 | 0 |
|  | 405957445 | Tyrosine-protein phosphatase non-receptor type 6 [*Crassostrea gigas*] | 0 | 6.293733 | 0 | 2.000033 | 1.6696 | 0 |
|  | 405973130 | UDP-glucose:glycoprotein glucosyltransferase 1 [*Crassostrea gigas*] | 17.49533 | 16.20933 | 15.069 | 31.56967 | 20.7 | 17.585 |
|  | 405952219 | Uncharacterized protein CXorf22 [*Crassostrea gigas*] | 6.997567 | 0 | 4.689733 | 0 | 1.758267 | 3.021387 |
|  | 405971256 | Uncharacterized protein y4xO [*Crassostrea gigas*] | 4.447867 | 4.316313 | 4.369167 | 2.787833 | 5.137367 | 6.922967 |
| c91906_g2 | 762098971 | UPF0573 protein C2orf70 homolog A-like [*Crassostrea gigas*] | 0.635567 | 0 | 0 | 0 | 0.358733 | 0.960567 |
|  | 405963822 | UPF0663 transmembrane protein C17orf28 [*Crassostrea gigas*] | 1.2711 | 2.3132 | 3.013967 | 10.20083 | 3.033667 | 5.2943 |
|  | 405963809 | Vacuolar protein sorting-associated protein 4B [*Crassostrea gigas*] | 6.0442 | 9.277467 | 7.3694 | 10.74017 | 13.62733 | 8.628667 |
|  | 405970234 | Very long-chain specific acyl-CoA dehydrogenase, mitochondrial [*Crassostrea gigas*] | 2.536933 | 0.998567 | 0 | 0 | 3.525367 | 3.717233 |
| c101861_g1 | 762144091 | vesicle-fusing ATPase 1-like [*Crassostrea gigas*] | 1.2775 | 1.6475 | 1.682467 | 1.338233 | 2.037233 | 7.942833 |
| c101543_g1 | 762168651 | vigilin-like isoform X1 [*Crassostrea gigas*] Vigilin [*Crassostrea gigas*] | 18.43267 | 23.463 | 14.42767 | 37.43433 | 15.13333 | 18.14133 |
| c91074_g1 | 762158557 | villin-1-like isoform X2 [*Crassostrea gigas*] | 19.70867 | 13.22733 | 24.14133 | 23.296 | 25.142 | 24.51833 |
| c102546_g1 | 762087607 | vinculin-like isoform X7 [*Crassostrea gigas*] | 0 | 4.2886 | 0 | 13.73127 | 6.178133 | 6.5822 |
|  | 405960135 | Voltage-dependent calcium channel subunit alpha-2/delta-2 [*Crassostrea gigas*] | 0.958133 | 16.88167 | 1.333133 | 4.842667 | 5.527167 | 2.272933 |
|  | 405972713 | von Willebrand factor D and EGF domain-containing protein [*Crassostrea gigas*] | 2.22604 | 29.809 | 0.668667 | 20.07073 | 10.244 | 1.6536 |
|  | 405969928 | von Willebrand factor D and EGF domain-containing protein [*Crassostrea gigas*] | 0.633433 | 5.293067 | 0.339733 | 5.524533 | 3.7201 | 0.640367 |
|  | 405968088 | WD repeat-containing protein 19 [*Crassostrea gigas*] | 0.955453 | 0.983733 | 1.683733 | 1.3583 | 2.0993 | 8.896667 |
|  | 405968838 | WD repeat-containing protein 63 [*Crassostrea gigas*] | 4.460067 | 5.3069 | 5.714433 | 1.6545 | 5.035433 | 6.48745 |
|  | 405970751 | WD repeat-containing protein 65 [*Crassostrea gigas*] | 6.672833 | 4.3015 | 6.0134 | 2.069433 | 7.148 | 9.9093 |
|  | 405954463 | WD repeat-containing protein C10orf79 [*Crassostrea gigas*] | 0.638733 | 1.32648 | 1.981 | 0 | 6.559 | 0.344567 |
|  | 405961750 | Xanthine dehydrogenase [*Crassostrea gigas*] | 8.901167 | 7.269467 | 10.0219 | 13.69233 | 10.66477 | 12.59667 |
| c93514_g1 | 762076444 | xylose isomerase-like [*Crassostrea gigas*] | 1.2711 | 2.3132 | 3.013967 | 8.981467 | 3.033667 | 5.2943 |
|  | 405966050 | Zinc finger RNA-binding protein [*Crassostrea gigas*] | 0.31672 | 0 | 2.348233 | 1.407633 | 2.0904 | 0.320187 |
| **c89544_g1** | **762113166** | **cAMP-dependent protein kinase catalytic subunit-like isoform X4 [*Crassostrea gigas*]** | **1.2669** | **0.9837** | **3.3483** | **0.0000** | **0.9831** | **3.9575** |
| **c100493_g1** | **762129333** | **guanine nucleotide-binding protein G(i) subunit alpha isoform X2 [*Crassostrea gigas*]** | **5.4081** | **2.6451** | **4.3663** | **4.1114** | **7.5023** | **7.2714** |
| **c43405_g1** | **762109239** | **guanine nucleotide-binding protein G(q) subunit alpha [*Crassostrea gigas*]** | **17.8213** | **9.5867** | **23.7657** | **16.5427** | **21.5413** | **26.8570** |
| **c82523_g2** | **762149532** | **guanine nucleotide-binding protein G(s) subunit alpha-like [*Crassostrea gigas*]** | **5.7259** | **2.6451** | **5.7036** | **4.1114** | **6.1915** | **6.2792** |
| **c86852_g1** | **762119229** | **myosin heavy chain, striated muscle-like isoform X2 [*Crassostrea gigas*]** | **1874.9667** | **832.0533** | **1881.7333** | **1053.8000** | **1250.1667** | **1835.9667** |
| **c81990_g2** | **762093595** | **paramyosin-like isoform X3 [*Crassostrea gigas*]** | **926.4467** | **121.5713** | **442.9200** | **391.3033** | **134.2067** | **344.8567** |
| **c98073_g1** | **871237491** | **RAC-gamma serine/threonine-protein kinase-like [*Aplysia californica*]** | **2.8600** | **0.3279** | **1.3590** | **2.4076** | **4.0789** | **1.0337** |
| **c89155_g1** | **762132875** | **serine/threonine-protein phosphatase 2A 65 kDa regulatory subunit A alpha isoform-like isoform X1 [*Crassostrea gigas*]** | **44.5110** | **35.7560** | **57.2440** | **37.5823** | **66.6267** | **74.9017** |
| **c84614_g1** | **762125802** | **serine/threonine-protein phosphatase 2A catalytic subunit beta isoform isoform X1 [*Crassostrea gigas*]** | **3.4998** | **5.6368** | **9.3354** | **1.6892** | **5.7751** | **7.8906** |
| **c100521_g2** | **762116649** | **serine/threonine-protein phosphatase PP1-beta catalytic subunit [*Crassostrea gigas*]** | **7.9530** | **9.6034** | **11.7357** | **9.3196** | **14.6990** | **13.5750** |
| **c53999_g1** | **762074186** | **tropomyosin isoform X2 [*Crassostrea gigas*]** | **43.2500** | **526.6200** | **97.4563** | **122.6140** | **217.3667** | **45.9137** |
| **c53999_g1** | **762074186** | **tropomyosin isoform X2 [*Crassostrea gigas*]** | **0.0000** | **0.0000** | **0.0000** | **0.3382** | **0.6820** | **0.0000** |
