# Supplementary material for: Proteome of larval metamorphosis induced by epinephrine in the Fujian oyster Crassostrea angulata
Source: BMC Genomics. 2020 Sep 29;21:675. doi: 10.1186/s12864-020-07066-z (PMC7525975; doi:10.1186/s12864-020-07066-z)
Supplement: Supplementary file 7 — Additional file 7: Supplementary Table 6. The differentially expressed proteins with the same name were merged among six groups. Supplementary Table 7. Extremely significant enrichment GO term both in PL-PA and PA-MET groups. Supplementary Table 8. Extremely significant enrichment GO term in PL-eSEN -einSEN groups. Supplementary Table 9. Extremely significant enrichment GO term in PL-MET -eMET groups. Supplementary Table 10. Extremely significant enrichment KEGG pathway of PL-MET group. Supplementary Table 11. Extremely significant enrichment pathway only in eSEN-eMET group against PL-eSEN group. Supplementary Table 12. Extremely significant enrichment pathway only in PL-einSEN group against PL-eSEN group. Supplementary Table 13. Protein abbreviations and corresponding full name [file 12864_2020_7066_MOESM7_ESM.doc]

**Supplementary table 6** The differentially expressed proteins with the same name were merged among six groups

| **Identified Proteins** | **Protein volumes** | | | | | |
| --- | --- | --- | --- | --- | --- | --- |
|  | **PL** | **PA** | **MET** | **einSEN** | **eSEN** | **eMET** |
| 14-3-3 protein[*Crassostrea gigas*] | 29.48 | 33.39 | 30.54 | 22.25 | 25.26 | 45.99 |
| SCO-spondin [*Crassostrea gigas*] | 46.18 | 73.40 | 49.52 | 41.89 | 45.34 | 175.84 |
| Dynein [*Crassostrea gigas*] | 48.47 | 29.11 | 2.58 | 33.54 | 35.99 | 16.33 |
| ubiquilin [*Crassostrea gigas*] | 4.22 | 8.23 | 8.98 | 4.04 | 5.40 | 11.58 |
| ubiquitin-conjugating enzyme E2 [*Crassostrea gigas*] | 2.21 | 4.00 | 3.75 | 1.48 | 2.90 | 3.20 |
| 26S protease regulatory subunit [*Crassostrea gigas*] | 7.65 | 9.52 | 13.75 | 5.19 | 8.15 | 6.28 |
| ATP synthase[Crassostrea ariakensis] | 32.30 | 32.04 | 27.27 | 27.15 | 29.45 | 28.42 |
| D-3-phosphoglycerate dehydrogenase-like [*Crassostrea gigas*] | 7.29 | 4.81 | 5.11 | 4.77 | 4.00 | 4.63 |
| enolase [*Crassostrea gigas*] | 77.55 | 70.41 | 68.04 | 60.26 | 67.92 | 70.98 |
| malate dehydrogenase[*Crassostrea gigas*] | 65.55 | 49.06 | 33.84 | 55.63 | 60.12 | 46.49 |
| 6-phosphogluconate dehydrogenase, decarboxylating [*Crassostrea gigas*] | 22.12 | 9.30 | 6.56 | 8.27 | 16.11 | 4.95 |
| Glucose-6-phosphate 1-dehydrogenase [*Crassostrea gigas*] | 9.95 | 6.10 | 0.00 | 0.64 | 2.35 | 0.99 |
| glucose-6-phosphate isomerase-like [*Crassostrea gigas*] | 1.92 | 0.00 | 0.00 | 0.00 | 0.00 | 2.64 |
| succinate dehydrogenase [*Crassostrea gigas*] | 18.63 | 15.57 | 7.71 | 16.32 | 14.17 | 12.57 |
| Pyruvate dehydrogenase E1 component subunit alpha type II [*Crassostrea gigas*] | 18.22 | 13.26 | 5.15 | 17.48 | 16.73 | 7.94 |
| mannose-6-phosphate isomerase-like [*Crassostrea gigas*] | 3.35 | 1.37 | 1.10 | 1.91 | 2.34 | 1.99 |
| citrate synthase, mitochondrial-like isoform X1 [*Crassostrea gigas*] | 40.40 | 25.72 | 15.58 | 28.62 | 33.81 | 32.09 |
| Pyridoxal-dependent decarboxylase domain-containing protein 1 [*Crassostrea gigas*] | 2.60 | 3.13 | 4.84 | 0.64 | 2.02 | 1.64 |
| isocitrate dehydrogenase [*Crassostrea gigas*] | 16.03 | 14.01 | 12.80 | 14.63 | 10.88 | 6.30 |
| cadherin [*Crassostrea gigas*] | 3.52 | 14.69 | 23.51 | 6.47 | 10.14 | 32.30 |
| Calmodulin [*Crassostrea gigas*] | 27.57 | 17.47 | 26.20 | 8.26 | 12.06 | 24.49 |
| RecName: Full=Calmodulin; Short=CaM | 24.51 | 11.69 | 29.54 | 7.63 | 7.70 | 16.24 |
| calmodulin-like [*Crassostrea gigas*] | 2.66 | 0.00 | 9.06 | 0.00 | 0.00 | 0.00 |
| calreticulin [Pinctada fucata] | 12.82 | 26.92 | 35.07 | 28.63 | 27.79 | 41.71 |
| caltractin-like [*Crassostrea gigas*] | 0.34 | 0.66 | 1.34 | 0.96 | 0.00 | 1.31 |
| calumenin-like isoform X2 [*Crassostrea gigas*] | 2.84 | 2.40 | 7.90 | 0.00 | 1.50 | 2.98 |
| calcium uniporter protein, mitochondrial-like [*Crassostrea gigas*] | 5.31 | 6.86 | 5.48 | 5.73 | 5.36 | 0.00 |
| Calcium/calmodulin-dependent protein kinase type II delta chain [*Crassostrea gigas*] | 10.61 | 1.37 | 0.00 | 1.59 | 0.99 | 0.00 |
| Calcium-binding mitochondrial carrier protein Aralar1 [*Crassostrea gigas*] | 3.06 | 0.00 | 1.10 | 0.64 | 1.01 | 0.33 |
| calcium-transporting ATPase sarcoplasmic/endoplasmic reticulum type-like [*Crassostrea gigas*] | 17.01 | 3.33 | 11.53 | 43.08 | 12.73 | 4.64 |
| Calnexin [*Crassostrea gigas*] | 11.20 | 6.15 | 20.96 | 12.72 | 10.04 | 3.31 |
| EF-hand calcium-binding domain-containing protein [*Crassostrea gigas*] | 2.90 | 3.40 | 0.00 | 3.39 | 2.11 | 4.07 |
| Troponin T [*Crassostrea gigas*] | 18.56 | 25.82 | 21.99 | 15.16 | 15.72 | 48.42 |
| Calpain [*Crassostrea gigas*] | 46.92 | 41.69 | 33.08 | 45.78 | 44.84 | 24.81 |
| cathepsin Z-like [*Crassostrea gigas*] | 14.40 | 22.58 | 33.59 | 26.08 | 15.74 | 18.85 |
| cathepsin L [*Crassostrea gigas*] | 7.45 | 20.34 | 33.57 | 10.74 | 5.86 | 28.04 |
| 60 kDa heat shock protein [*Crassostrea gigas*] | 152.24 | 191.71 | 288.74 | 93.00 | 128.82 | 225.76 |
| heat shock 70 kDa protein [*Crassostrea gigas*] | 10.97 | 14.63 | 19.62 | 9.65 | 10.03 | 15.76 |
| heat shock protein 27-like [*Crassostrea gigas*] | 0.00 | 1.31 | 7.59 | 0.00 | 0.33 | 2.32 |
| IgGFc-binding protein-like [*Crassostrea gigas*] | 5.62 | 7.55 | 21.66 | 2.54 | 6.02 | 20.82 |
| hepatic lectin-like [*Crassostrea gigas*] | 3.30 | 1.74 | 3.06 | 1.91 | 1.32 | 4.64 |
| superoxide dismutase [Cu-Zn]-like isoform X1 [*Crassostrea gigas*] | 22.81 | 30.86 | 50.33 | 26.71 | 28.80 | 60.57 |
| Galectin-6 [*Crassostrea gigas*] | 25.50 | 28.34 | 24.03 | 37.84 | 35.80 | 18.54 |
| glutathione S-transferase A-like [*Crassostrea gigas*] | 8.57 | 2.05 | 3.77 | 3.50 | 3.35 | 2.98 |
| glutathione S-transferase omega-1-like [*Crassostrea gigas*] | 6.62 | 7.20 | 5.82 | 4.45 | 5.38 | 5.42 |
| glutathione S-transferase P 1-like [*Crassostrea gigas*] | 13.26 | 12.01 | 9.60 | 3.81 | 11.06 | 6.95 |
| universal stress protein A-like protein [*Crassostrea gigas*] | 13.05 | 6.51 | 4.49 | 9.73 | 11.05 | 2.45 |
| galectin-9-like isoform X2 [*Crassostrea gigas*] | 6.94 | 4.83 | 2.14 | 5.40 | 5.01 | 2.98 |
| superoxide dismutase [Mn][*Crassostrea gigas*] | 4.65 | 0.98 | 0.00 | 6.36 | 8.03 | 0.00 |
| Catalase [*Crassostrea gigas*] | 24.63 | 20.82 | 8.59 | 27.66 | 30.45 | 22.16 |
| eosinophil peroxidase-like isoform X2 [*Crassostrea gigas*] | 38.65 | 6.11 | 0.00 | 53.13 | 51.56 | 44.98 |
| Peroxidasin [*Crassostrea gigas*] | 44.33 | 11.15 | 0.00 | 21.95 | 39.07 | 46.66 |
| Peroxiredoxin [*Crassostrea gigas*] | 26.34 | 43.18 | 44.66 | 33.86 | 29.49 | 63.71 |
| Kyphoscoliosis peptidase [*Crassostrea gigas*] | 2.92 | 5.32 | 4.84 | 3.18 | 3.34 | 5.13 |
| radial spoke head protein [*Crassostrea gigas*] | 24.79 | 34.27 | 17.20 | 23.52 | 22.34 | 46.42 |
| mitogen-activated protein kinase [*Crassostrea gigas*] | 4.18 | 2.62 | 1.51 | 3.18 | 3.68 | 1.65 |
| mucin-17-like [*Crassostrea gigas*] | 34.10 | 47.71 | 45.49 | 35.93 | 34.13 | 104.23 |
| mucin-19-like [*Crassostrea gigas*] | 0.00 | 0.69 | 0.00 | 2.86 | 0.66 | 4.95 |
| mucin-2-like [*Crassostrea gigas*] | 0.00 | 1.97 | 1.77 | 0.00 | 0.00 | 3.30 |
| mucin-like protein [*Crassostrea gigas*] | 1.98 | 16.58 | 0.00 | 1.59 | 0.66 | 1.00 |
| mucin-5AC-like [*Crassostrea gigas*] | 5.52 | 11.62 | 12.06 | 7.00 | 8.03 | 27.13 |
| V-type proton ATPase subunit [*Crassostrea gigas*] | 11.54 | 10.63 | 9.71 | 13.91 | 11.11 | 10.92 |
| ran-specific GTPase-activating protein-like [*Crassostrea gigas*] | 2.83 | 6.84 | 13.61 | 2.86 | 3.00 | 10.43 |
| Ras-like GTP-binding protein RHO [Crassostrea ariakensis] | 5.93 | 2.75 | 8.87 | 9.85 | 10.37 | 3.29 |
| ras-like protein 3 isoform X2 [*Crassostrea gigas*] | 5.27 | 2.43 | 3.44 | 3.50 | 4.02 | 0.00 |
| Ras-related protein Rab-10 [*Crassostrea gigas*] | 13.92 | 6.78 | 5.87 | 7.31 | 7.04 | 4.62 |
| ras-related protein Rab-14 [*Crassostrea gigas*] | 10.28 | 5.12 | 1.38 | 4.45 | 5.68 | 1.64 |
| ras-related protein Rab-35-like [*Crassostrea gigas*] | 13.57 | 2.65 | 3.77 | 6.68 | 5.69 | 4.30 |
| ras-related protein Rab-7a [*Crassostrea gigas*] | 12.31 | 9.86 | 13.33 | 13.03 | 8.71 | 5.61 |
| Rho GTPase-activating protein 17 [*Crassostrea gigas*] | 1.38 | 0.00 | 3.80 | 0.00 | 2.31 | 0.00 |
| soma ferritin-like [*Crassostrea gigas*] | 0.00 | 0.72 | 0.33 | 3.18 | 0.33 | 5.95 |
| cold shock domain-containing protein 3-like [*Crassostrea gigas*] | 10.50 | 26.11 | 29.94 | 66.76 | 46.19 | 60.87 |
| Spectrin [*Crassostrea gigas*] | 67.73 | 110.39 | 116.54 | 15.58 | 38.79 | 210.09 |
| cilia- and flagella-associated protein [*Crassostrea gigas*] | 12.46 | 7.05 | 1.72 | 7.47 | 9.87 | 5.29 |
| Neurogenic locus Notch protein [*Crassostrea gigas*] | 0 | 4.21 | 4.77 | 0 | 1.91 | 13.89 |

**Supplementary Table 7** Extremely significant enrichment GO term both in PL-PA and PA-MET groups

| **GO term** | **Description** |
| --- | --- |
| **Biological process** |  |
| GO:0006850 | mitochondrial pyruvate transport |
| GO:0006913 | nucleocytoplasmic transport |
| GO:0019722 | calcium-mediated signaling |
| GO:0034314 | Arp2/3 complex-mediated actin nucleation |
| GO:0051169 | nuclear transport |
| GO:0051050 | positive regulation of transport |
| GO:0007160 | cell-matrix adhesion |
| GO:0006165 | nucleoside diphosphate phosphorylation |
| GO:0030155 | regulation of cell adhesion |
| GO:0043484 | regulation of RNA splicing |
| GO:0046497 | nicotinate nucleotide metabolic process |
| GO:0048024 | regulation of mRNA splicing, via spliceosome |
| GO:0032239 | regulation of nucleobase-containing compound transport |
| GO:0032241 | positive regulation of nucleobase-containing compound transport |
| **Cellular component** |  |
| GO:0030532 | small nuclear ribonucleoprotein complex |
| GO:0030904 | retromer complex |
| GO:0097525 | spliceosomal snRNP complex |
| GO:0005635 | nuclear envelope |
| **Molecular function** |  |
| GO:0005509 | calcium ion binding |
| GO:0043115 | precorrin-2 dehydrogenase activity |
| GO:0004418 | hydroxymethylbilane synthase activity |

**Supplementary Table 8** Extremely significant enrichment GO term in PL-eSEN -einSEN groups

| **only in PL-eSEN and eSEN-einSEN groups** | | **only in PL-einSEN and eSEN-einSEN groups** | |
| --- | --- | --- | --- |
| **GO term** | **Description** | **GO term** | **Description** |
| **biological_process** |  | **biological_process** |  |
| GO:0030155 | regulation of cell adhesion | GO:0016117 | carotenoid biosynthetic process |
| GO:0034968 | histone lysine methylation | GO:0019321 | pentose metabolic process |
|  |  | GO:0019872 | streptomycin biosynthetic process |
|  |  | GO:0007157 | heterophilic cell-cell adhesion via plasma membrane cell adhesion molecules |
| **cellular_component** |  | **cellular_component** |  |
| GO:0008290 | F-actin capping protein complex | GO:0030127 | COPII vesicle coat |
| **molecular_function** |  | **molecular function** |  |
| GO:0005184 | neuropeptide hormone activity | GO:0004797 | thymidine kinase activity |
| GO:0004161 | dimethylallyltranstransferase activity | GO:0016805 | dipeptidase activity |
| GO:0051087 | chaperone binding | GO:0030354 | melanin-concentrating hormone activity |
| GO:0033743 | peptide-methionine (R)-S-oxide reductase activity | GO:0005509 | calcium ion binding |
|  |  | GO:0015288 | porin activity |

**Supplementary Table 9 Extremely significant enrichment GO term in PL-MET -eMET groups**

| **only in PL-MET and MET-eMET groups** | | **only in PL-eMET and MET-eMET groups** | | **only in PL-MET and PL-eMET groups** | |
| --- | --- | --- | --- | --- | --- |
| **GO term** | **Description** | **GO term** | **Description** | **GO term** | **Description** |
| **Biological process** |  | **Biological process** |  | **Biological process** |  |
| GO:0007157 | heterophilic cell-cell adhesion via plasma membrane cell adhesion molecules | GO:0043934 | sporulation | GO:0006222 | UMP biosynthetic process |
| GO:0009435 | NAD biosynthetic process | GO:0019521 | D-gluconate metabolic process | GO:0006850 | mitochondrial pyruvate transport |
| GO:0019674 | NAD metabolic process |  |  | GO:0009877 | nodulation |
| GO:0034627 | 'de novo' NAD biosynthetic process |  |  | GO:0018101 | protein citrullination |
| GO:0043650 | dicarboxylic acid biosynthetic process |  |  | GO:0030153 | bacteriocin immunity |
|  |  |  |  | GO:0030155 | regulation of cell adhesion |
|  |  |  |  | GO:0034314 | Arp 2/3 complex-mediated actin nucleation |
|  |  |  |  | GO:0042619 | poly-hydroxybutyrate biosynthetic process |
|  |  |  |  | GO:0007156 | homophilic cell adhesion via plasma membrane adhesion molecules |
|  |  |  |  | GO:0006511 | ubiquitin-dependent protein catabolic process |
|  |  |  |  | GO:0018874 | benzoate metabolic process |
| **Cellular component** |  | **Cellular component** |  | **Cellular component** |  |
| GO:0016533 | cyclin-dependent protein kinase 5 holoenzyme complex | GO:0005833 | hemoglobin complex | GO:0036128 | CatSper complex |
| GO:0036125 | fatty acid beta-oxidation multienzyme complex | GO:0000221 | vacuolar proton-transporting V-type ATPase, V1 domain |  |  |
| **Molecular function** |  | **Molecular function** |  | **Molecular function** |  |
| GO:0004797 | thymidine kinase activity | GO:0004367 | glycerol-3-phosphate dehydrogenase [NAD^+^] activity | GO:0000772 | mating pheromone activity |
| GO:0008948 | oxaloacetate decarboxylase activity | GO:0004560 | alpha-L-fucosidase activity | GO:0004336 | galactosylceramidase activity |
| GO:0016534 | cyclin-dependent protein kinase 5 activator activity |  |  | GO:0004615 | phosphomannomutase activity |
| GO:0016805 | dipeptidase activity |  |  | GO:0008761 | UDP-N-acetylglucosamine 2-epimerase activity |
| GO:0051539 | 4 iron, 4 sulfur cluster binding |  |  | GO:0016822 | hydrolase activity, acting on acid carbon-carbon bonds |
| GO:0030170 | pyridoxal phosphate binding |  |  | GO:0016823 | hydrolase activity, acting on acid carbon-carbon bonds, in ketonic substances |
|  |  |  |  | GO:0033743 | peptide-methionine (R)-S-oxide reductase activity |
|  |  |  |  | GO:0005509 | calcium ion binding |
|  |  |  |  | GO:0043115 | precorrin-2 dehydrogenase activity |

**Supplementary Table 10** Extremely significant enrichment KEGG pathway of PL-MET group

| Term | Pathway ID | Gene Number | *P* value |
| --- | --- | --- | --- |
| Propanoate metabolism | ko00640 | 8 | 0.000409 |
| Alanine, aspartate and glutamate metabolism | ko00250 | 8 | 0.000925 |
| Other glycan degradation | ko00511 | 5 | 0.001493 |
| Butanoate metabolism | ko00650 | 6 | 0.001506 |
| Biosynthesis of antibiotics | ko01130 | 27 | 0.003638 |
| Valine, leucine and isoleucine degradation | ko00280 | 10 | 0.004381 |
| Glycosphingolipid biosynthesis - ganglio series | ko00604 | 2 | 0.005243 |
| Butirosin and neomycin biosynthesis | ko00524 | 1 | 0.008116 |
| Phosphonate and phosphinate metabolism | ko00440 | 1 | 0.008116 |
| Fatty acid elongation | ko00062 | 3 | 0.009963 |
| Carbon metabolism | ko01200 | 19 | 0.010021 |
| Microbial metabolism in diverse environments | ko01120 | 23 | 0.010313 |
| Biosynthesis of secondary metabolites | ko01110 | 30 | 0.012829 |
| Fatty acid degradation | ko00071 | 6 | 0.015795 |
| Caprolactam degradation | ko00930 | 2 | 0.017299 |
| Nicotinate and nicotinamide metabolism | ko00760 | 3 | 0.017562 |
| beta-Alanine metabolism | ko00410 | 5 | 0.018184 |
| Biosynthesis of unsaturated fatty acids | ko01040 | 2 | 0.02071 |
| Tryptophan metabolism | ko00380 | 6 | 0.020916 |
| Styrene degradation | ko00643 | 1 | 0.026351 |
| Citrate cycle (TCA cycle) | ko00020 | 7 | 0.027852 |
| Glycosphingolipid biosynthesis | ko00603 | 2 | 0.028557 |
| Aminobenzoate degradation | ko00627 | 2 | 0.028557 |
| Pentose and glucuronate interconversions | ko00040 | 3 | 0.031111 |
| Fatty acid metabolism | ko01212 | 5 | 0.03986 |
| Primary bile acid biosynthesis | ko00120 | 1 | 0.042986 |
| Limonene and pinene degradation | ko00903 | 1 | 0.042986 |
| Lysine degradation | ko00310 | 6 | 0.045277 |
| Sphingolipid metabolism | ko00600 | 2 | 0.048297 |

**Supplementary Table 11** Extremely significant enrichment pathway only in eSEN-eMET group against PL-eSEN group

| Term | Pathway ID | Gene Number | P value |
| --- | --- | --- | --- |
| **Organismal Systems** |  |  |  |
| Mineral absorption | ko04978 | 2 | 0.004686 |
| Glucagon signaling pathway | ko04922 | 11 | 0.00883 |
| Salivary secretion | ko04970 | 8 | 0.008905 |
| Adrenergic signaling in cardiomyocytes | ko04261 | 11 | 0.009419 |
| Adipocytokine signaling pathway | ko04920 | 6 | 0.014297 |
| Thyroid hormone synthesis | ko04918 | 6 | 0.015674 |
| Bile secretion | ko04976 | 5 | 0.01693 |
| Circadian rhythm | ko04710 | 2 | 0.02014 |
| Renin-angiotensin system | ko04614 | 2 | 0.029922 |
| Insulin secretion | ko04911 | 4 | 0.029969 |
| Fc gamma R-mediated phagocytosis | ko04666 | 6 | 0.030231 |
| Ovarian steroidogenesis | ko04913 | 3 | 0.04356 |
| **Metabolism** |  |  |  |
| Glycosphingolipid biosynthesis - ganglio series | ko00604 | 2 | 0.009369 |
| Butirosin and neomycin biosynthesis | ko00524 | 1 | 0.01217 |
| Alanine, aspartate and glutamate metabolism | ko00250 | 7 | 0.012886 |
| Glycosaminoglycan degradation | ko00531 | 3 | 0.020157 |
| Tyrosine metabolism | ko00350 | 4 | 0.03312 |
| Various types of N-glycan biosynthesis | ko00513 | 3 | 0.034581 |
| Tropane, piperidine and pyridine alkaloid biosynthesis | ko00960 | 1 | 0.038825 |

**Supplementary Table 12** Extremely significant enrichment pathway only in PL-einSEN group against PL-eSEN group

| Term | Pathway ID | Gene Number | P value |
| --- | --- | --- | --- |
| Glucagon signaling pathway | ko04922 | 11 | 001328 |
| Insulin signaling pathway | ko04910 | 13 | 0.001394 |
| Adrenergic signaling in cardiomyocytes | ko04261 | 10 | 0.004452 |
| B cell receptor signaling pathway | ko04662 | 5 | 0.007108 |
| Cholinergic synapse | ko04725 | 6 | 0.007361 |
| Long-term potentiation | ko04720 | 7 | 0.020752 |
| Natural killer cell mediated cytotoxicity | ko04650 | 4 | 0.021985 |
| Chemokine signaling pathway | ko04062 | 6 | 0.025761 |
| Melanogenesis | ko04916 | 7 | 0.026023 |
| Fc epsilon RI signaling pathway | ko04664 | 4 | 0.026167 |
| T cell receptor signaling pathway | ko04660 | 5 | 0.027755 |
| Long-term depression | ko04730 | 4 | 0.028436 |
| Prolactin signaling pathway | ko04917 | 4 | 0.030826 |
| GnRH signaling pathway | ko04912 | 7 | 0.032167 |
| Dorso-ventral axis formation | ko04320 | 4 | 0.033339 |
| Osteoclast differentiation | ko04380 | 5 | 0.033925 |
| Axon guidance | ko04360 | 5 | 0.033925 |
| Glutamatergic synapse | ko04724 | 5 | 0.033925 |
| Aldosterone-regulated sodium reabsorption | ko04960 | 2 | 0.040417 |
| Estrogen signaling pathway | ko04915 | 8 | 0.040526 |
| Taste transduction | ko04742 | 1 | 0.041185 |
| Vasopressin-regulated water reabsorption | ko04962 | 3 | 0.042228 |
| Retrograde endocannabinoid signaling | ko04723 | 4 | 0.047788 |
| Serotonergic synapse | ko04726 | 4 | 0.047788 |
| Dopaminergic synapse | ko04728 | 8 | 0.049974 |

**Supplementary table 13** Protein abbreviations and corresponding full name

**Nodes:**

Network nodes represent proteins

*splice isoforms or post-translational modifications are collapsed, i.e. each node represents all the proteins produced by a single, protein-coding gene locus.*

**Edges:**

Edges represent protein-protein associations

*associations are meant to be specific and meaningful, i.e. proteins jointly contribute to a shared function; this does not necessarily mean they are physically binding each other.*

| **Input:** | |  |
| --- | --- | --- |
| **Abbreviations** | **Proteins name** | **Function** |
| metap1d | Methionine aminopeptidase 1D, mitochondrial (338 aa) | Aminopeptidase, Hydrolase, Protease |
| eif6 | Eukaryotic translation initiation factor 6 (245 aa) | Protein biosynthesis, Ribosome biogenesis |
| eif3i | Eukaryotic translation initiation factor 3 subunit I (325 aa) | Protein biosynthesis, Ribosome biogenesis |
| eif4g3a | Eukaryotic translation initiation factor 4 gamma, 3 (1561 aa) | mRNA binding, translation |
| rsph9 | Radial spoke head protein 9 homolog (277 aa) | Cilium movement; |
|  |  | Axoneme assembly |
| RSPH1 | Radial spoke head 1 homolog (Chlamydomonas) (232 aa) | Axoneme assembly |
| ttll3 | Tubulin monoglycylase TTLL3 (789 aa) | Axoneme assembly, cilium assembly |
| ttc25 | Tetratricopeptide repeat protein 25 (486 aa) | Motor protein |
| DNAH3 | Dynein, axonemal, heavy chain 3 (3868 aa) | Motor protein |
| dnah5 | Dynein, axonemal, heavy chain 5 (1974 aa) | Motor protein |
| dnal1 | Dynein light chain 1, axonemal (192 aa) | Activator, Motor protein |
| dnah7 | Dynein, axonemal, heavy chain 7 (4001 aa) | Motor protein |
| PARM1 | Prostate androgen-regulated mucin-like protein 1 (319 aa) | Apoptotic process |
| postna | Periostin, osteoblast specific factor a (1016 aa) | Cell adhesion; extracellular matrix organization |
| calm1a | Calmodulin 1a (149 aa) | Response to calcium ion |
| TPM1 | Tropomyosin alpha-1 chain (327 aa) | Actin-binding, Muscle protein |
| cnn1a | Calponin (287 aa) | Actin-binding, Calmodulin-binding |
| ttnb | Titin, tandem duplicate 1; Titin b (27263 aa) | Muscle contraction |
| calml4a | Calmodulin-like 4a (153 aa) | Calcium-mediated signaling |
| parp1 | Poly (ADP-ribose) polymerase family, member 1 (1013 aa) | DNA-binding, Glycosyltransferase, Transferase |
| rpa1 | Replication protein A 70 kDa DNA-binding subunit (601 aa) | DNA-binding |
| kdm5bb | Lysine-specific demethylase 5B-B (1522 aa) | Oxidoreductase, Transcription regulation |
| uggt1 | UDP-glucose glycoprotein glucosyltransferase 1 (1554 aa) | Glycosyltransferase, Transferase |
| cbr4 | Carbonyl reductase family member 4 (237 aa) | Oxidoreductase, Fatty acid biosynthesis |
| cat | Catalase (526 aa) | Mitogen, Oxidoreductase, Peroxidase |
| cycsb | Cytochrome c; Electron carrier protein. (104 aa) | Oxidoreductase |
| hsdl2 | Hydroxysteroid dehydrogenase-like protein 2 (415 aa) | Oxidoreductase |
| xdh | Xanthine dehydrogenase (1351 aa) | Oxidoreductase |
| hpda | 4-hydroxyphenylpyruvate dioxygenase (388 aa) | Oxidoreductase, Tyrosine catabolism |
| ppp1r3cb | Protein phosphatase 1 regulatory subunit 3C-B (317 aa) | Carbohydrate metabolism, Glycogen metabolism |
| h6pd | Hexose-6-phosphate dehydrogenase (glucose 1-dehydrogenase) (791 aa) | Carbohydrate metabolism, Glucose metabolism |
| sucla2 | Succinate--CoA ligase [ADP-forming] subunit beta, mitochondrial (466 aa) | Tricarboxylic acid cycle |
| idh3a | Isocitrate dehydrogenase [NAD] subunit, mitochondrial (365 aa) | Tricarboxylic acid cycle |
| notch1a | Neurogenic locus notch homolog protein 1 (1655 aa) | Activator, Developmental protein, Receptor; Angiogenesis, Differentiation, Notch signaling pathway |
| tdrd1 | Tudor domain-containing protein 1 (1176 aa) | Developmental protein |
| arf3b | Novel protein similar to human ADP-ribosylation factor 1 (ARF1) (181 aa) | GTP binding |
| canx | Calnexin (600 aa) | Chaperone |
| COX1 | Cytochrome c oxidase subunit 1 (516 aa) | Chaperone |
| haao | 3-hydroxyanthranilate 3,4-dioxygenase (287 aa) | Pyridine nucleotide biosynthesis |
| fah | Fumarylacetoacetate hydrolase (fumarylacetoacetase) (185 aa) | Tyrosine catabolism, Hydrolase |
| ctdnep1b | CTD nuclear envelope phosphatase 1B; (245 aa) | Hydrolase, Protein phosphatase |
| rtn4ip1 | Reticulon-4-interacting protein 1 homolog, mitochondrial (387 aa) | Neurogenesis |
| HNRNPK | Heterogeneous nuclear ribonucleoprotein K (72 aa) | Activator, DNA-binding; Transcription regulation |
| hnrnph1 | Heterogeneous nuclear ribonucleoprotein H1 (403 aa) | mRNA processing |
| sf3b3 | Splicing factor 3B subunit 3 (1217 aa) | mRNA processing |
| prpf39 | Pre-mRNA-processing factor 39 (752 aa) | mRNA processing |
| cobl | Protein cordon-bleu (708 aa) | Actin-binding |
| ap2a1 | AP-2 complex subunit alpha; Component of the adaptor protein complex 2 (AP-2). (959 aa) | Endocytosis, Protein transport, Transport |
| pacsin2 | Protein kinase C and casein kinase substrate in neurons 2 (490 aa) | Endocytosis |

**Your Current Organism:**

Danio rerio

*NCBI taxonomy Id:* [*7955*](https://www.ncbi.nlm.nih.gov/Taxonomy/Browser/wwwtax.cgi?id=7955) *Other names: Brachydanio rerio, Brachydanio rerio frankei, Cyprinus rerio, D. rerio, Danio frankei, Danio rerio, Danio rerio frankei, leopard danio, zebra danio, zebra fish, zebrafish*
