# Supplementary material for: Proteome of larval metamorphosis induced by epinephrine in the Fujian oyster Crassostrea angulata
Source: BMC Genomics. 2020 Sep 29;21:675. doi: 10.1186/s12864-020-07066-z (PMC7525975; doi:10.1186/s12864-020-07066-z)
Supplement: Supplementary file 8 — Additional file 8: Supplementary Table 14. Primer of qPCR [file 12864_2020_7066_MOESM8_ESM.doc]

**Supplementary Table 14** Primer of qPCR

| **Abbreviation** | **Protein** | **Primer** |
| --- | --- | --- |
| EF1 | Elongation factor 1 | ACCACCCTGGTGAGATCAAG |
|  |  | ACGACGATCGCATTTCTCTT |
| UBQ | Ubiquitin | ATAGAGGCGTTGCATGAGC  ACGGAGGACCAAGTGAAGG |
| Calm | calmodulin | CAACATAACGAGGAACAAG |
|  |  | CACAACACAGGTACAATAC |
| GST | glutathione S-transferase | TGGAGACATCGTGGTTAA |
|  |  | GTCAGTAGGAATGAGTTGTG |
| HYOU | hypoxia up-regulated 1 | CACTAATCATCACCGACCAT |
|  |  | CCACAGAACCACCATAGAG |
| PTPN11 | tyrosine-protein phosphatase non-receptor type 11 | GAAGATACCAGGCAGATAT  GGTCAGTGTCAGTATATGT |
| PLCPI  PSMB | Leukocyte cysteine proteinase inhibitor 1  proteasome subunit beta | CTCCTCACTTGTCTCCTC  GGTCAGAAGATGCGAAAG  CCTTGCCAACAGACGACAAA  CACGGCCATAACATCCACTG |
